# Deep mRNA Sequencing of the *Tritonia diomedea* Brain Transcriptome Provides Access to Gene Homologues for Neuronal Excitability, Synaptic Transmission and Peptidergic Signalling

**DOI:** 10.1371/journal.pone.0118321

**Published:** 2015-02-26

**Authors:** Adriano Senatore, Neranjan Edirisinghe, Paul S. Katz

**Affiliations:** 1 Neuroscience Institute, Georgia State University, Atlanta, Georgia, United States of America; 2 IS&T, Georgia State University, Atlanta, Georgia, United States of America; Queen’s University Belfast, UNITED KINGDOM

## Abstract

**Background:**

The sea slug *Tritonia diomedea* (Mollusca, Gastropoda, Nudibranchia), has a simple and highly accessible nervous system, making it useful for studying neuronal and synaptic mechanisms underlying behavior. Although many important contributions have been made using *Tritonia*, until now, a lack of genetic information has impeded exploration at the molecular level.

**Results:**

We performed Illumina sequencing of central nervous system mRNAs from *Tritonia*, generating 133.1 million 100 base pair, paired-end reads. *De novo* reconstruction of the RNA-Seq data yielded a total of 185,546 contigs, which partitioned into 123,154 non-redundant gene clusters (unigenes). BLAST comparison with RefSeq and Swiss-Prot protein databases, as well as mRNA data from other invertebrates (gastropod molluscs: *Aplysia californica*, *Lymnaea stagnalis* and *Biomphalaria glabrata*; cnidarian: *Nematostella vectensis*) revealed that up to 76,292 unigenes in the *Tritonia* transcriptome have putative homologues in other databases, 18,246 of which are below a more stringent E-value cut-off of 1x10-6. *In silico* prediction of secreted proteins from the *Tritonia* transcriptome shotgun assembly (TSA) produced a database of 579 unique sequences of secreted proteins, which also exhibited markedly higher expression levels compared to other genes in the TSA.

**Conclusions:**

Our efforts greatly expand the availability of gene sequences available for *Tritonia diomedea*. We were able to extract full length protein sequences for most queried genes, including those involved in electrical excitability, synaptic vesicle release and neurotransmission, thus confirming that the transcriptome will serve as a useful tool for probing the molecular correlates of behavior in this species. We also generated a neurosecretome database that will serve as a useful tool for probing peptidergic signalling systems in the *Tritonia* brain.

## Background

The properties of the brains of gastropod molluscs provide advantages for studying the neural mechanisms of learning, memory, and behavior [1]. Gastropods, such as the sea slug *Tritonia diomedea*, have large, accessible neurons that are identifiable from specimen to specimen and whose electrical activity and synaptic connectivity can be understood in the context of simple behaviors. For example, the central pattern generator (CPG) circuit underlying a rhythmic escape swimming behavior produced by *Tritonia* has been elucidated [2,3]. The CPG consists of just three bilaterally represented neuronal types. Indeed, the simplicity and accessibility of the *Tritonia* swim CPG circuit, as well as other gastropod and invertebrate CPG circuits, have provided important insights into the fundamental cellular and synaptic elements required for establishing and controlling self-driven rhythmic circuits [4]. Notably, in gastropods, CPG studies have been typically constrained to electrophysiological characterization of neuronal excitability and synaptic connectivity; little headway has been made towards understanding these systems at the level of genes and molecules. A pre-requisite for such exploration is the knowledge of gene sequences, which can be used to monitor when and where particular genes are expressed and to manipulate gene expression for functional characterization. Currently, only a small number of gastropod species have had their transcriptomes sequenced. These in include *Aplysia californica* [5], *Lymnaea stagnalis* [6,7] and *Lottia gigantea* [8]. For other species, few gene sequences are available. In the case of *Tritonia diomedea* for example, there are only 15 mRNA sequences available on NCBI, and an EST database is available containing 7,105 highly fragmented mRNA sequences (NCBI taxonomy ID 70853). Fortunately, recent advances in next-generation DNA sequencing technology, such as Illumina sequencing, have provided a means to overcome limitations in access to gene sequence data. Now, a single sequencing effort can produce thousands of sequences quickly and at a relatively low cost. Furthermore, advances in bioinformatic tools have made it possible to assemble mRNA transcriptome shotgun assemblies (TSAs) without a requirement of a reference genome (i.e. *de novo* assembly [9]), which is highly advantageous for non-mainstream models such as *Tritonia* for which a genome sequence is not available.

We generated a neuronal TSA for *Tritonia diomedea*, by Illumina sequencing of mRNAs from the central nervous system and *de novo* transcript reconstruction using the program Trinity [10,11]. A BLAST comparison with RefSeq and SwissProt protein databases, as well as with mRNA/TSA sequences from other invertebrates indicates that the assembly contains a vast amount of genetic information, with more than 18,000 unigene sequences finding strong homology in the other databases. Gene ontology mapping corroborates a diversity of gene content and produces a strikingly similar GO term distribution as other molluscan transcriptomes [5,7,12,13]. The *Tritonia* TSA contains a vast number of transcripts with full to nearly full-length protein coding sequences, allowing for the extraction of *Tritonia* gene homologues that underlie neuronal excitability, synaptic transmission, and neuropeptide secretion.

## Results

### Sequencing and assembly of the *Tritonia diomedea* CNS transcriptome

Messenger RNAs (mRNAs) from the *Tritonia diomedea* central nervous system (CNS) consisting of the cerebral, pleural, pedal, and buccal ganglia, were sequenced on an Illumina HiSeq2500 platform. This generated a total of 133,156,930 2x100 base pair paired-end reads, with 94.49% of all bases having Phred (Q) scores [14] above 30 (indicative of good sequencing quality) (Table 1). The program Sickle (https://github.com/najoshi/sickle) was used to remove uncertain bases/reads from the data. Reads were then reconstructed into contiguous sequences (contigs) using the *de novo* assembler Trinity [10,11] and contaminating sequences were removed (see methods). This generated a total of 185,546 contigs, which grouped into 123,154 components (e.g. compX in the Trinity nomenclature) and 144,415 sub-components (e.g. compX_cY_1_, compX_cY_2_ etc.). Together, components and their corresponding sub-components represent grouped sequences of alternatively-spliced gene isoforms, polymorphic gene variants, and/or closely-related gene paralogues [10,11] (Table 1). Notably, the total number of assembled transcript sequences is elevated when compared to an expected gene content of ~24,000 based on genome predictions from the related gastropod mollusc *Lottia gigantea* [8]. This is a common consequence of *de novo* reconstruction brought about in part by fragmentation of gene sequences, assembly artefacts, contamination by non-coding RNA or genomic DNA, and low-level contamination from outside sources [9,15–17].

**Table 1:** Metrics for the *Tritonia* TSA and predicted peptides.

**Raw Illumina data**	
Number of reads	133,156,930
%Phred score ≥ 30	94.49
**Assembled contigs**	
Trinity transcripts	185,546
Trinity sub-components	144,415
Trinity components	123,154
%GC	39.4
N_50_	1,353 nt
Longest contig	19,496 nt
Median contig length	363 nt
Average contig length	74 nt
Total bases	137,742,822 nt
**Predicted peptides**	
Total ORFs	33,778
Total LCPC ORFs	15,439
Average length (aa)	435 aa
Median length (aa)	308 aa
Longest peptide	5,075 aa

Abbreviations: amino acids (aa), nucleotides (nt), longest contig per component (LCPC), open reading frames (ORFs).

**Fig 1:**
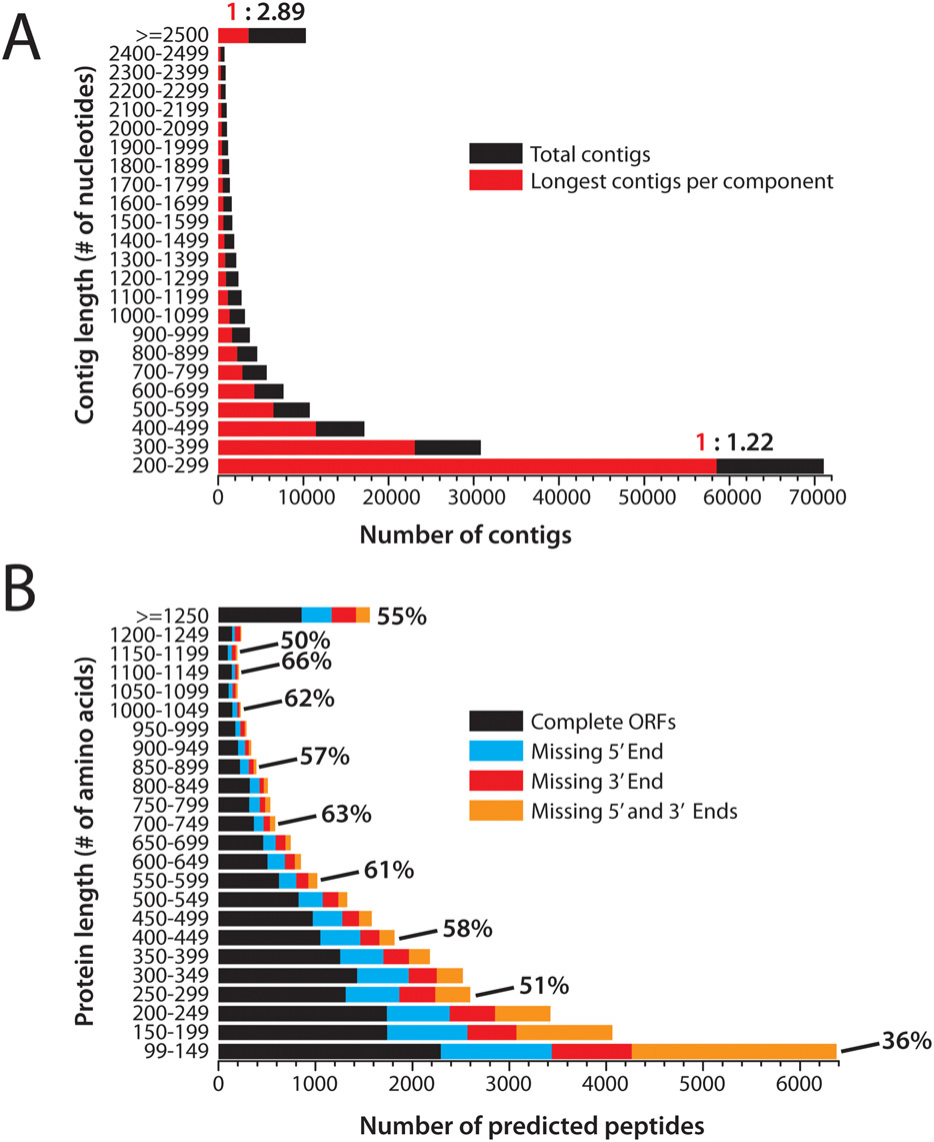
Length histograms of the *Tritonia* Trinity-assembled transcripts and TransDecoder-predicted peptide sequences. **A)** The length distribution of contigs (black bars) and longest contig per component (LCPC; red bars) reveals that a major portion of Trinity-assembled sequences range between 200 to 399 nucleotides in length, and that transcript and LCPC counts drop rapidly with increasing length. **B)** Length histogram of longest reading frames predicted from Trinity transcripts using TransDecoder. The bars are partitioned to show the percentage of open reading frames (ORFs) that are complete (black bars), missing the 5’ end (cyan), missing the 3’ end (red), or missing both 5’ and 3’ ends (orange bars). The percentage of complete ORFs increases with length, where only 36% are complete with lengths between 99 and 149 amino acids, whereas 55% are complete above 1,250 amino acids with a peak of 66% between 1,100 and 1,149 amino acids (red text).

Metrics for the *Tritonia* transcriptome shotgun assembly (TSA) are shown in Table 1. A length histogram of these sequences reveals that more than 50% fall between 200 and 399 nucleotides (nt) in length (Fig. 1A, black bars), a pattern mirrored when only considering the longest contig per Trinity component (LCPC), which interpret to represent unique gene sequences (i.e. unigenes) (Fig. 1A, red bars). Notably, the ratio of total transcripts to LCPC transcripts increases as length increases; in the small length range between 200 to 299 nucleotides, there are almost as many transcripts as there are LCPC unigene sequences (i.e. ratio of 1.22:1), whereas above 2,500 nucleotides, there are almost 3 transcripts for every LCPC sequence (2.89:1). This pattern reflects an increasing number of alternatively-spliced sequences, grouped into Trinity components, as length increases. This is not surprising given that longer genes tend to contain more exons providing greater opportunity for transcript variation.

*In silico* translation of the transcriptome dataset with TransDecoder [11] produced a similar declining distribution in number of protein sequences with increasing length, but with a slower rate of decline; more than 50% of the total 33,778 predicted sequences fell within 100 to 349 amino acids (aa) in length (Fig. 1B, Table 1). A majority of the predicted peptides contain full length open reading frames (ORFs; 17,313 or 51.3%), whereas the remaining 16,465 are missing either the 5’ end (i.e. N-terminus; 6,631 or 19.6%), the 3’ end (C-terminus; 4,296 or 12.7%), or both ends (5,538 or 16.4%) (Fig. 1B). Extracting only the longest predicted peptide for each Trinity component yielded 15,439 unique protein sequences, of which 41.4% are complete (6,397), 21.7% are missing a portion of the N-terminus (3,344), 11.1% the C-terminus (1,719), and 25.8% both N- and C-termini (3,979) (S1 Fig.). Thus, the inflated number of Trinity-assembled unigenes/components is in large part due to fragmentation of transcript sequences into separate contigs. However, a rough estimate would indicate that 26.7% of the *Tritonia* transcribed genome is represented by full-length ORFs in our TransDecoder data (6,397 unique protein sequences out of ~24,000 genes). For fragmented ORFs, we were frequently able to extend peptide sequences by manually searching for missing fragments (detailed below), indicating that although Trinity failed to resolve some transcript/peptide junctions, the information to do so is nevertheless present in the raw data. Indeed, as *de novo* assembly methods improve, the degree of gene fragmentation will drop significantly. For this reason, we have included our raw, untrimmed Illumina data along with the *Tritonia* transcriptome assembly and the predicted peptide sequences as NCBI BioProject PRJNA252890.

### BLAST annotation

**Fig 2:**
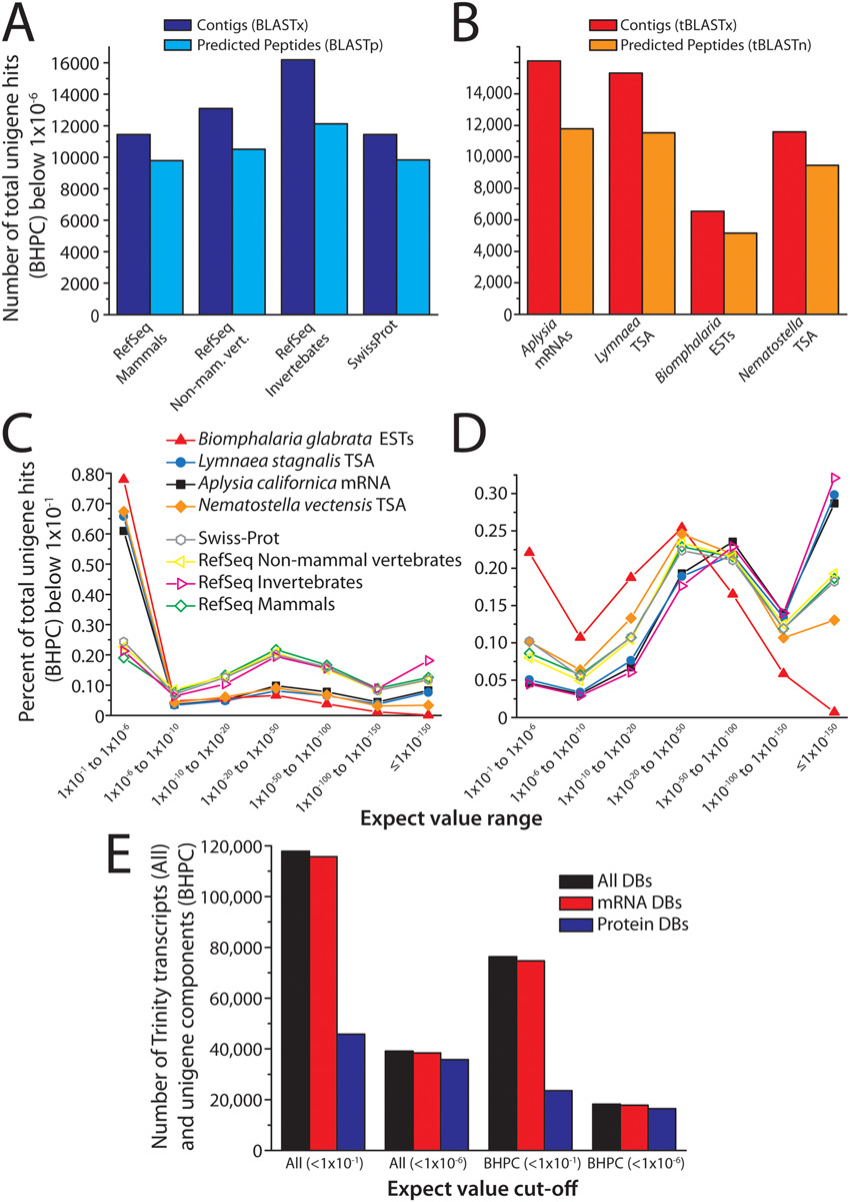
BLAST annotation of the *Tritonia* TSA. **A)** Total number of unigene hits with E-values below 1x10^-6^ when comparing the *Tritonia* TSA (dark blue bars) and predicted peptide sequences (light blue bars) against the RefSeq mammalian, RefSeq non-mammalian vertebrate, RefSeq invertebrate, and Swiss-Prot protein databases. We define unigenes as single representative contigs/transcripts from each Trinity component, selected based on the highest BLAST BitScore within each component (best hit per component or BHPC). There is a consistent pattern of reduced hits when comparing BLAST results from the *Tritonia* TSA contigs to the TransDecoder predicted protein sequences, indicating that protein prediction fails to detect a significant number of ORFs that are nevertheless detected by BLAST. **B)** Total number of unigene hits (BHPC) with E-values below 1x10^-6^ when comparing the *Tritonia* TSA (red bars) and predicted peptide sequences (orange bars) against mRNA sequence data from gastropod molluscs *Aplysia californica* (NCBI mRNAs with taxonomy ID 6500), the *Lymnaea stagnalis* (CNS TSA [7]), *Biomphalaria glabrata* (ESTs from NCBI with taxid 6526), and cnidarian *Nematostella vectensis* (TSA [19]). **C)** Percent of total unigene BLAST hits (BHPC; y-axis) that fall within different E-value ranges (x-axis), for BLAST comparisons of *Tritonia* TSA contigs with the Swiss-Prot and RefSeq protein databases, and the invertebrate mRNA datasets. Note that when compared to the well curated Swiss-Prot and RefSeq protein databases, the invertebrate mRNA datasets produce proportionately many more low scoring hits between 1x10^-1^ and 1x10^-6^ (i.e. 60.9 to 77.9% of hits for mRNA datasets vs. 19.0 to 24.3% for the protein databases), and fewer with strong scores below 1x10^-20^. **D)** The E-value distribution of BHPC BLAST hits changes significantly for TransDecoder predicted peptides compared to TSA sequences, with *Aplysia californica* mRNA and *Lymnaea stagnalis* peptide sequences, as well as those in the invertebrate partition of RefSeq, producing relatively fewer low scoring hits between 1x10^-1^ and 1x10^-6^ (4.5 to 5.0%), compared to RefSeq mammalian (8.6%), RefSeq non-mammalian vertebrates (8.0%), and Swiss-Prot (10.2%). Conversely, the percent of BLAST hits with very good scores (below 1x10^-150^) are much higher for RefSeq invertebrate (32.1%), *A.californica* (28.7%), and *L.stagnalis* (29.8%) than for RefSeq non-mammalian vertebrate (19.3%), RefSeq mammalian (18.6%), and Swiss-Prot (18.2%). Notably, *Biomphalaria glabrata* ESTs produce the poorest distribution of BLAST E-values, for both the *Tritonia* TSA and the predicted peptide sequences. **E)** Breakdown of the total number of *Tritonia* TSA sequences that produce BLAST hits in at least one of the four protein databases (Swiss-Prot and the three RefSeq partitions) or one of the four invertebrate mRNA datasets. When using a low E-value cut-off of 1x10^-1^, a total of 117,868 TSA sequences find hits in at least one database/dataset (black bar), and many more hits are found in the mRNA datasets (115,710; red bar) than in the protein databases (45,795; blue bar). At an increased E-value stringency below 1x10^-6^, only 39,137 TSA sequences find BLAST hits, which is mostly the same pool of sequences that find hits in the mRNA datasets (38,388), and the protein databases (35,801). This pattern is similar for unigene TSA sequences (BHPC), where of the total 18,246 that produce BLAST hits with scores below 1x10^-6^ in at least one of the eight databases/datasets, 17,789 produce hits in the mRNA datasets and 16,478 produce hits in the protein databases.

NCBI BLAST [18] (2.2.28+) was used to compare the *Tritonia* TSA (BLASTx) and predicted peptides (BLASTp) to Swiss-Prot and various partitions of the RefSeq protein database: mammalian (RefSeqMam), non-mammalian vertebrate (RefSeqNMV), and invertebrate (RefSeqInv). To quantify BLAST results, we counted the total number of *Tritonia* TSA and peptide sequences producing high scoring pairs (i.e. BLAST hits) with E-value scores below a designated cut-off (i.e. total hits), and we also counted unigene hits by extracting the best scoring hit per Trinity component (BHPC). Setting a E-value cut-off of 1x10^-6^ revealed that the *Tritonia* TSA and peptide sequences found the most BLAST hits in RefSeqInv and the fewest in Swiss-Prot (Fig. 2A, S1 Table). These results are not surprising, given that RefSeqInv sequences are phylogenetically closer to those of *Tritonia*, and that the Swiss-Prot database is considerably small than all other databases (i.e. RefSeqInv: 738,982 sequences; RefSeqNMV: 772,610; RefSeqMam: 1,762,320; Swiss-Prot: 542,503).

We also compared the *Tritonia* transcriptome and predicted peptides with mRNA sequences from other gastropods: *Aplysia californica* mRNAs (NCBI taxid 6500), *Lymnaea stagnalis* TSA [7], and *Biomphalaria glabrata* ESTs (NCBI taxid 6526) and from the cnidarian *Nematostella vectensis* TSA [19]. Most apparent is that the recent CNS transcriptome of *Lymnaea*, which is the result of a single Illumina sequencing effort [7], produces almost as many hits as all NCBI mRNA sequences for *Aplysia* (Fig. 2B, S2 Table). Also notable is the relatively low level of homology between *Tritonia* sequences and *Biomphalaria* expressed sequence tag (EST) sequences. 77.9% of *Tritonia* TSA and 22.1% of *Tritonia* peptide sequences produce unigene hits with low scores (E-values between 1x10^-1^ and 1x10^-6^) when aligned against Biomphalaria ESTs (S2 Table), even when compared to *Nematostella*, which, based on phylogeny, is expected to have more divergence in gene sequences relative to *Tritonia* (Fig. 2C and D). A possible explanation for this inconsistency is that EST sequences are inherently small and fragmented, which reduces alignment length and hence lowers scores. Conversely, the *Tritonia* TSA and peptide sequences find the most homology with *Aplysia* and *Lymnaea* mRNA sequences, especially the *Tritonia* peptides where 28.7% and 29.8% of unigene hits produce E-values below 1x10^-150^ (Fig. 2C and D, S2 Table).

For BLAST results, there was a considerable reduction in the number of unigene hits when comparing *Tritonia* TSA with those of predicted peptides (Fig. 2A and B). This suggests a considerable loss of sequence information upon peptide prediction. However, predicted peptides in general produce stronger alignment scores (Fig. 2D vs. C), whereas the majority of BLASTx/tBLASTx alignments of the TSA produce low scores clustered between 1x10^-1^ and 1x10^-6^. Many of these are likely to be artifactual alignments (Fig. 2C and D, S1 and S2 Tables). Another striking contrast occurs between the *Tritonia* TSA—invertebrate mRNA BLAST alignments compared to the *Tritonia* TSA—Swiss-Prot/RefSeq protein BLAST alignments; a large proportion of TSA-mRNA alignments produce low scores (i.e. 60.9 to 77.9% between 1x10^-1^ and 1x10^-6^, and 0.1 to 8.3% below 1x10^-150^) whereas TSA-protein alignments produce much higher scores in general (i.e. only 19.0 to 24.3% between 1x10^-1^ and 1x10^-6^, and 11.6 to 18.1% below 1x10^-150^) (Fig. 2C, S1 and S2 Tables). When considering only *Tritonia* peptide BLAST alignments, *Aplysia* and *Lymnaea* mRNAs and RefSeqInv proteins produce the best scores, whereas *Biomphalaria* and *Nematostella* produce the lowest (Fig. 2D, S1 and S2 Tables). As mentioned above, the poor alignment scores for *Biomphalaria* are unexpected given its comparatively close relation to *Tritonia*, however the remaining E-value distributions are as expected, where databases with phylogenetically closer sequences produce better alignments (i.e. *Aplysia*/*Lymnaea*/RefSeqInv > RefSeqMam/RefSeqNMV/Swiss-Prot > *Nematostella*; Fig. 2D).

In total, 117,868 *Tritonia* Trinity-assembled TSA sequences and 76,292 Trinity components find BLAST hits in at least one of the 8 databases with scores below 1x10^-1^ (RefSeqMam, RefSeqNVM, RefSeqInv, Swiss-Prot, *Aplysia* mRNAs, *Lymnaea* TSA, *Biomphalaria* ESTs, and *Nematostella* TSA) (Fig. 2E). Although the invertebrate mRNA sequences account for most of these hits, at a higher stringency of 1x10^-6^ the contributions from the mRNA and the RefSeq/Swiss-Prot protein databases are nearly the same, indicating that more or less the same subset of 39,137 Trinity transcripts and 18,246 unigene BHPC components are finding strong BLAST hits across all of these databases (Fig. 2E).

### Comparison of the *Tritonia* TSA with previously generated *Tritonia* EST data

In order to evaluate the content of our Illumina-generated *Tritonia* CNS transcriptome with respect to a previous Sanger-based EST sequencing effort (7,105 sequences from juvenile CNS mRNAs; NCBI taxonomy ID 70853), we performed reciprocal BLASTn analysis with the two datasets. Using a very stringent expect value cut-off of 1x10^-75^, 79.3% of EST sequences found near-identical to identical homology in a *Tritonia* TSA subject database, with 4,402 of the 7,105 of EST sequences (62.0%) producing expect values approximating zero (Table 2). Conversely, BLASTn of *Tritonia* TSA sequences against an EST subject database, only produced 3,303 total hits (1.8% of the 185,546 TSA sequences) and 1,419 BHPC unigene hits (1.2% of the 123,154 components). This indicates that 98.2% of *Tritonia* TSA sequences, and 98.8% of unigenes/components, are novel and not found in the EST database. We also noted that a much higher proportion of EST sequences find stringent homology in the *Tritonia* TSA, compared to TSA sequences finding homology in the EST sequences (i.e. compare 5,636 EST sequences vs. only 1,419 unigene TSA sequences). Such a discrepancy was also reported for the similarly sequenced *Lymnaea stagnalis* CNS TSA when compared to a corresponding Sanger-sequenced EST database [6,7]. This inconsistency was attributed to much longer contigs of the Illumina-derived TSA aligning with several independent EST sequences [7]. This is consistent with our finding, where the 5,636 EST sequences that find homology in the *Tritonia* TSA (expect value cut-off 1x10^-75^) all align to the same set of 1,358 unigene sequences. Clearly, these comparisons confirm that the present sequencing effort has benefitted greatly from recent advantages in mRNA sequencing, yielding more complete and comprehensive sequences for *Tritonia* than previously available.

**Table 2:** Reciprocal BLASTn comparisons of the *Tritonia* TSA and Tritonia EST sequences.

Number of *Tritonia* TSA sequences with a BLASTn hit in the Tritonia EST database					Number of *Tritonia* EST sequences with a BLASTn hit in the Tritonia TSA database		
Expect value	All	% of Total	BHPC	% of Total	Expect value	All	% of Total
≤ 1x10^-75^, > 1x10^-100^	288	0.2%	73	0.1%	≤ 1x10^-75^, > 1x10^-100^	223	3.1%
≤ 1x10^-100^, > 1x10^-125^	338	0.2%	87	0.1%	≤ 1x10^-100^, > 1x10^-125^	323	4.5%
≤ 1x10^-125^, > 1x10^-150^	160	0.1%	80	0.1%	≤ 1x10^-125^, > 1x10^-150^	278	3.9%
≤ 1x10^-150^, > 1x10^-175^	232	0.1%	82	0.1%	≤ 1x10^-150^, > 1x10^-175^	313	4.4%
≤ 1x10^-175^, > 1x10^-200^	44	0.0%	19	0.0%	≤ 1x10^-175^, > 1x10^-200^	97	1.4%
≤ 1x10^-200^, = 0	2,241	1.2%	1078	0.9%	≤ 1x10^-200^, = 0	4,402	62.0%
Sum Total	3,303	1.8%	1,419	1.2%	Sum Total	5,636	79.3%
Total Trans/Comps	**185,546**		**123,154**		Total ESTs	**7,105**	

### Gene ontology mapping of *Tritonia* gene sequences

**Fig 3:**
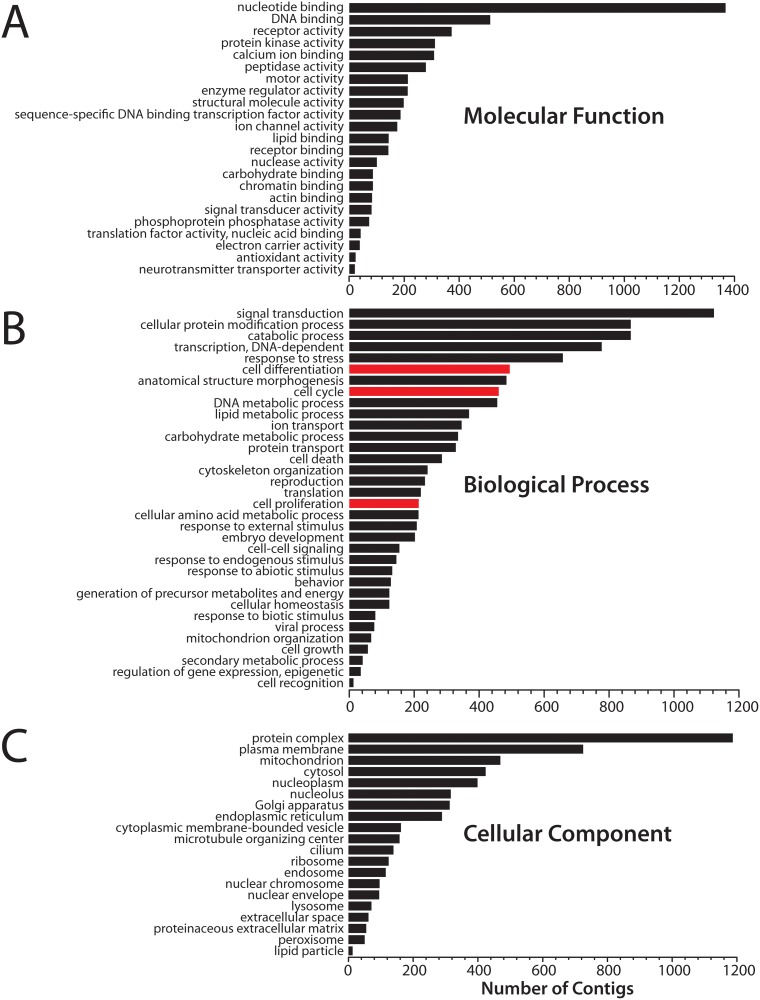
Combined graphs of gene ontology mappings for *Tritonia* TSA unigene sequences. **A)** Number of unigene Trinity contigs (BHPC against Swiss-Prot; bottom axis) having GO-terms for molecular function (i.e. multi-level GO terms filtered by number of sequences). The most prominent GO assignments for the *Tritonia* TSA include binding activity, such as nucleotide and DNA binding, and receptor activity. **B)** Number unigene contigs with multi-level GO terms associated with biological function. The red bars illustrate that a significant number of genes from the *Tritonia* TSA are mapped with terms for cell proliferation and differentiation (cell cycle = 460; cellular proliferation = 214; cellular differentiation = 494), which is unexpected given that most neurons in the adult CNS are expected to be non-proliferative and terminally differentiated.

Sequences from the *Tritonia* TSA were assigned gene ontology (GO) mappings at the protein level using Blast2GO [20]. Because Trinity outputs multiple “gene-isoforms”, clustered as components, for any given gene sequence [10,11], directly attempting large-scale GO annotation on a Trinity data set would result in over-representation of subsets of genes with multiple isoforms. To circumvent this, we isolated single gene sequences from each component by filtering by BHPC when BLAST was conducted against the UniProtKB Swiss-Prot protein database (BLASTx with an E-value cut-off of 1x10^-3^). This produced a total of 12,599 unique transcript sequences, which were imported into Blast2GO along with their corresponding Swiss-Prot BLAST results. GO-mappings were inferred based on BLAST data (using a cut-off of 1x10^-6^) and InterproScan protein domain analysis [21] (using default parameters). GO mappings were obtained for 9,388 unigene sequences, and their assigned GO terms were then simplified using a generic GO-slim vocabulary. Combined graphs summarizing GO assignments were generated (multi-level, filtered by sequence number) in the three major branches for gene ontology [22]: molecular function (Fig. 3A), biological process (Fig. 3B), and cellular component (Fig. 3C).

As expected for a CNS transcriptome, the *Tritonia* CNS TSA is enriched for proteins with molecular functions of binding, including nucleotide binding (e.g. G proteins bind GTP/GDP for intracellular signalling), DNA binding (e.g. gene expression, chromatin modification, etc.), and Ca^2+^ binding (e.g. calcium signalling and sequestration), as well as receptor activity (Fig. 3A). Some of the most prevalent biological processes represented by GO annotation include signal transduction, protein modification, and gene transcription, but it is interesting that 11.1% of annotations were classified under processes involving cell division and differentiation (Fig. 3B, red bars), given that most neurons in the *Tritonia* CNS are expected to be terminally differentiated and non-proliferative [23]. The most prevalent GO assignments for cellular component include integration into protein complexes, localization to the plasma membrane, and localization to mitochondria (Fig. 3C). Overall, the complexity and abundance of GO terms that map onto the *Tritonia* CNS TSA indicate that the data set captures a broad spectrum of genetic information.

### *In silico* generation of a neurosecretome database from the *Tritonia* CNS

Expression levels of *Tritonia* transcripts were estimated using RSEM, a program that effectively maps raw reads onto *de novo* assembled sequences to produce corresponding transcripts per million (TPM) scores [24]. Of note, RSEM outputs TPM values for Trinity sub-components to quantify expression of “genes”, since individual transcripts within a shared a sub-component can vary significantly in TPM value, and single components can contain paralogous gene sequences partitioned into separate sub-components [11]. The thirty most abundantly expressed “genes” (i.e. sub-components) in the *Tritonia* CNS, for which we could find BLAST hits below 1x10^-4^ in either Swiss-Prot, RefSeqInv, RefSeqNMV, RefSeqMam or *Aplysia* mRNAs, are shown in Table 3. As expected, the most abundantly expressed genes include cytoskeletal proteins, and proteins involved in translation and energy metabolism. Remarkable is that the three most abundant gene transcripts, and ten out of the top thirty, are homologous to molluscan secretory neuropeptides: Cerebral peptide 1 [25,26], Pedal peptide 1 [27,28], Abdominal ganglion neuropeptide R3–1 [29,30], Molluscan insulin related peptide (MIP) 3 [31] and MIP 7 [32], L5 neuropeptide [33], Neuroactive polyprotein R15 [34], PTSP-like peptide [5], small cardioactive peptide [35], and sodium-influx-stimulating peptide [36]. Also in the top thirty is the *Tritonia* homologue of the glia-secreted acetylcholine-binding protein, which in the mollusc brain serves to modulate cholinergic synapses [37,38].

**Table 3:** BLAST annotation of the top 30 most abundantly-expressed genes in the *Tritonia* TSA, with Hit Description and Function of secreted proteins bolded and italicized.

ComponentID	TPM	BitScore	E-Val	Species	Subject Accession	Name	Function
comp41991_c0	13036	170	5.E-47	*Aplysia californica*	Q10998	***Cerebral peptide 1***	***Neuropeptide***
comp68470_c0	10195	54.7	7.E-07	*Aplysia californica*	AY833134.1	***Pedal peptide-1***	***Neuropeptide***
comp51703_c0	8645	44.7	1.E-04	*Aplysia californica*	P01364	***Abdominal ganglion neuropeptide R3–14***	***Neuropeptide***
comp68518_c1	8204	187	2.E-60	*Pseudopodoces humilis*	XP_005524281.1	Polyubiquitin-C	Protein homeostasis/regulation
comp71320_c3	7161	850	0.E+00	*Aplysia californica*	NP_001191530.1	Beta tubulin	Cytoskeleton
comp41988_c0	7129	84.7	5.E-19	*Lymnaea stagnalis*	P80090	***Molluscan insulin-related peptide 3***	***Neuropeptide***
comp41990_c0	6318	120	2.E-28	*Saccoglossus kowalevskii*	NP_001171863.1	***Acetylcholine-binding protein***	***Secreted Ach receptor***
comp18379_c0	5874	325	6.E-91	*Aplysia californica*	EZ114798.1	Soma ferritin	Iron homeostasis
comp70659_c1	5314	1017	0.E+00	*Aplysia californica*	AF481056.1	Alpha tubulin 2	Cytoskeleton
comp62529_c0	5123	182	5.E-46	*Aplysia californica*	NM_001204490.1	Memory suppression protein	Fatty acid binding
comp51849_c0	4613	243	0.E+00	*Aplysia californica*	XM_005113378.1	Uncharacterized protein	N/A
comp70507_c1	4445	897	0.E+00	*Aplysia californica*	U01352.1	Actin	Cytoskeleton
comp62384_c0	4442	48.7	2.E-05	*Aplysia californica*	EZ114614.1	Thymosin beta-12	Cytoskeleton
comp59184_c0	4437	93.6	3.E-18	*Aplysia californica*	M13649.1	***L5 neuropeptide***	***Neuropeptide***
comp65742_c0	3873	797	0.E+00	*Roboastra europaea*	NP_702975.1	Cytochrome c oxidase subunit I	ATP synthesis
comp70065_c7	3685	57.4	9.E-14	*Aplysia californica*	XM_005110939.1	Septin-1	Cytokinesis
comp65963_c1	3514	55.6	3.E-07	*Aplysia californica*	XM_005096893.1	***Neuroactive polyprotein R15***	***Neuropeptide***
comp71549_c0	3257	287	4.E-96	*Branchiostoma floridae*	XP_002602335.1	40S ribosomal protein S8	Protein synthesis
comp41985_c0	2974	788	0.E+00	*Branchiostoma floridae*	XP_002604724.1	Elongation factor 1-alpha	Protein synthesis/transport
comp71548_c0	2813	118	9.E-30	*Lymnaea stagnalis*	P91797	***Molluscan insulin-related peptide 7***	***Neuropeptide***
comp18350_c0	2698	110	2.E-25	*Trichinella spiralis*	XP_003366348.1	Uncharacterized protein	N/A
comp68118_c3	2644	185	6.E-54	*Aplysia californica*	NM_001204682.1	***PTSP-like peptide***	***Neuropeptide***
comp71553_c0	2208	77.4	2.E-13	*Xiphophorus maculatus*	XP_005800490.1	Frizzled-2	Wnt signalling
comp41984_c0	2196	355	2.E-97	*Aplysia californica*	HM163175.1	Calmodulin	Calcium signalling
comp65749_c0	1970	380	8.E-105	*Aplysia californica*	EZ114747.1	Cytochrome c oxidase subunit III	ATP synthesis
comp71558_c0	1966	174	1.E-43	*Aplysia californica*	XM_005110736.1	40S ribosomal protein S25	Protein synthesis
comp51746_c0	1890	1123	0.E+00	*Aplysia californica*	XM_005097955.1	Heat shock protein 70	Stress Response
comp67389_c0	1886	162	2.E-45	*Lymnaea stagnalis*	O97374	***Small cardioactive peptide***	***Neuropeptide***
comp41987_c1	1879	70.1	6.E-13	*Aplysia californica*	XP_005101466.1	***Sodium-influx-stimulating peptide***	***Neuropeptide***
comp71559_c0	1866	190	8.E-60	*Aplysia californica*	NP_001191522.1	Ribosomal protein L27a	Protein synthesis

**Fig 4:**
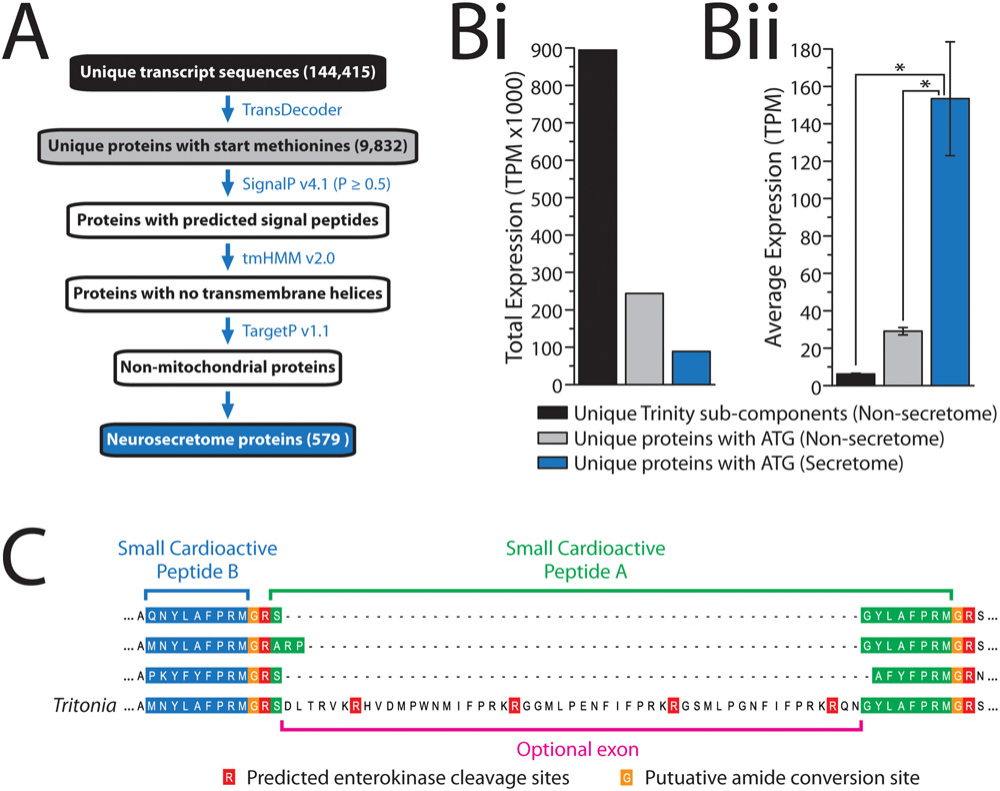
Generation of a neurosecretome database of the *Tritonia* brain. **A)** Schematic overview of the bioinformatics pipeline used to generate the neurosecretome database. TransDecoder translation of the 144,415 Trinity sub-component sequences produced 9,832 non-redundant proteins bearing N-terminal start methionines, and these were filtered for the presence of signal peptides (identified with the program SignalP), absence of transmembrane helices (with tmHMM), and absence of mitochondrial targeting signals (TargetP), producing a total of 579 predicted neurosecretome proteins. **Bi)** The sum TPM expression value for all neurosecretome proteins accounts for 26.67% of the sum TPM value for all 9,832 non-redundant proteins, and 9.93% of all sub-component gene sequences. **Bii)** The average TPM value for the neurosecretory proteins (153.38 ± 30.44 standard error) is 5.26-fold higher than the average of all non-redundant proteins (excluding neurosecretome proteins; 29.13 ± 2.00), and 24.67-fold higher than all sub-component gene sequences (also excluding neurosecretome proteins; 6.22 ± 0.40). The asterisks indicate probability values below 0.000 for one-way analysis of variance analysis comparing average TPM values. **C)** Section of a MUSCLE protein alignment of various small cardioactive peptide pre-pro-protein homologues from gastropod molluscs illustrates a novel optional exon discovered in the *Tritonia* TSA that alters the N-terminal amino acid coding sequence of SCP-A and introduces three novel putative neuropeptide repeats, each ending with the residues IFPRK. Arginine (R) residues predicted by the NeuroPred algorithm as convertase cleavage sites are depicted with a red background, potential glycine residues that are likely converted to amides have an orange background, and the SCP-B and SCP-A peptide sequences have blue and green backgrounds, respectively.

To further characterize *Tritonia* secretory proteins, we used a bioinformatic approach to generate a neurosecretome database of 579 unique protein sequences (Fig. 4A, S1 Dataset, S3 Table), predicted from a total of 9,832 non-redundant, non-overlapping protein sequences bearing start codons (derived from the 144,415 Trinity sub-components; see materials and methods). Briefly, secreted peptides were required to 1) bear SignalP-predicted [39] signal peptides required for co-translational insertion into the endoplasmic reticulum and the secretory pathway, 2) lack transmembrane helices predicted with tmHMM [40], and 3) lack mitochondrial targeting motifs as predicted by TargetP [41]. The 579 neurosecretome proteins constitute a small fraction of *Tritonia* sequences, representing only 5.89% of all start-codon-bearing proteins, and only 0.40% of all Trinity sub-components. Despite their small number, and in accordance with Table 3, neurosecretome gene expression is very high. Summing TPM values for the 579 Trinity sub-components corresponding to neurosecretome proteins produces a value of 88,806.44, which accounts for 26.68% of the sum TPM value of all 9,832 non-redundant protein sequences (i.e. 332,896.67). Perhaps more striking is that 9.93% of the sum TPM value for all 144,415 Trinity sub-components, 982,966.01, is accounted for by neurosecretome proteins (Fig. 4Bi). Similarly, the average sub-component TPM expression value for the 579 neurosecretome proteins is ~5.3-fold higher than that of the remaining 9,253 non-redundant proteins, and ~24.7-fold higher than the remaining 143,836 Trinity-subcomponents (Fig. 4Bii). We should caution however that the comparison between neurosecretome genes and all other sub-components are likely partially exaggerated, since the 144,415 Trinity sub-components are expected to collapse into a considerably smaller number once fragmented transcript sequences are combined and contaminating non-coding RNA sequences, genomic DNA sequences, and other contaminants are removed.

Eight of the eleven secreted proteins listed in Table 3, which were identified via BLAST homology, are found amongst the 428 full-length and 151 C-terminally-truncated neurosecretome proteins, including the *Tritonia* homologue for small cardioactive peptide (SCP). Interestingly, upon closer inspection of this particular neuropeptide pre-pro-protein, we found alternatively spliced variants in the TSA, encoding an optional exon situated between the classically described SCP-B and SCP-A, which when inserted alters the sequence of SCP-A from **S**GYLARFPRMamide to **QN**GYLARFPRMamide. Furthermore, it contains novel repeating peptide sequences flanked by molluscan convertase cleavage sites (predicted using the NeuroPred prediction server [42]), which may constitute novel neurosecretory peptide repeats (Fig. 4C).

**Fig 5:**
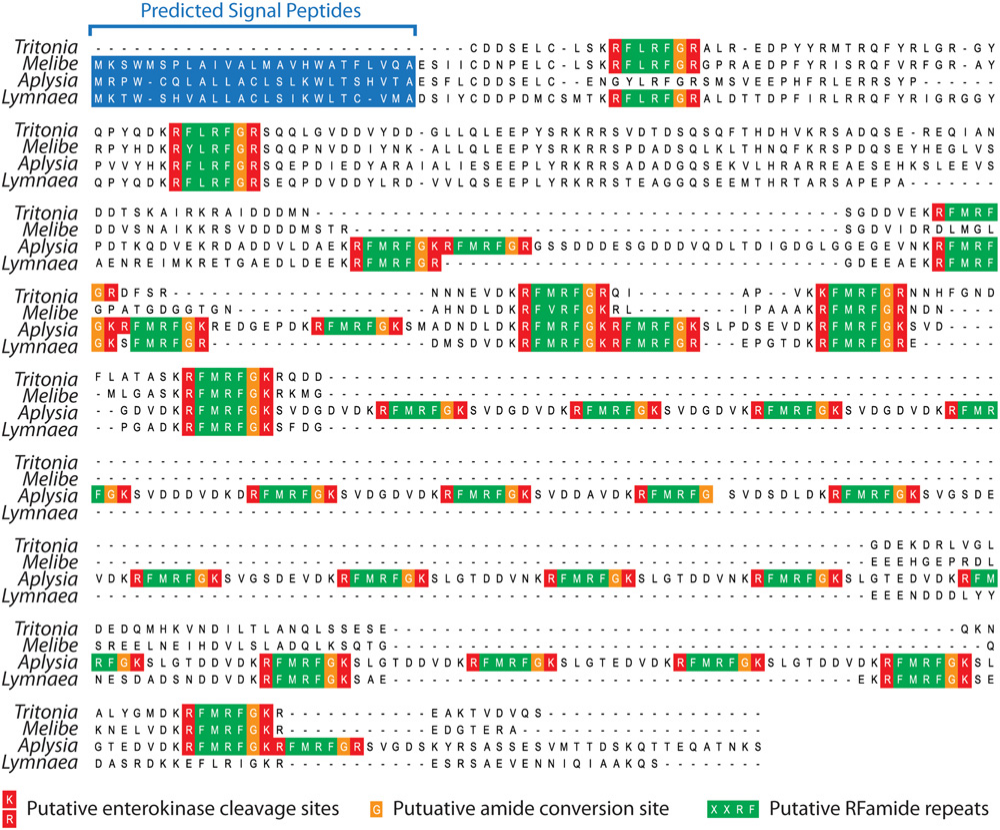
Absence of a signal peptide of the *Tritonia* FMRFamide homologue due to incomplete assembly. A MUSCLE protein alignment of FMRFamide homologues from *Tritonia*, fellow Nudibranch *Melibe leonina*, and fellow gastropods *Aplysia californica* (UniProt accession no. P08021) and *Lymnaea stagnalis* (UniProt accession no. P08021) reveals the absence of a signal peptide at the *Tritonia* FMRFamide N-terminus caused by incomplete assembly of this particular gene transcript. The *Aplysia* pre-pro-peptide had 29 FMRFamide repeats compared to 13 in *Lymnaea*, 7 in *Tritonia*, and 6 in *Melibe*. Amino acids corresponding to SignalP-predicted signal peptides are shown with a blue background, arginine (R) and lysine (K) residues corresponding to putative convertase cleavage sites are depicted with a red background, and potential glycines that are converted to amides have an orange background.

There are three secreted protein homologues listed in Table 3 that are not present in the neurosecretome database: Pedal peptide 1, Abdominal ganglion neuropeptide R3–1, and MIP 3). Pedal peptide 1 and Abdominal ganglion neuropeptide R3–1 have corresponding transcript sequences that are truncated and lack N-terminal coding sequences, which precluded them from the analysis due to the inability to evaluate the presence of signal peptides, whereas the MIP 3 transcript encodes a complete protein sequence but lacks a signal peptide. Indeed, we were able to identify MIP 3 as a non-classically secreted protein using the program SecretomeP [43], however many other genes identified in this manner were homologous to cytosolic, nuclear, and mitochondrial proteins, so this data was not included with our analysis. The absence of the three *Tritonia* homologues for secreted proteins listed in Table 3 from the neurosecretome database indicates that additional secreted proteins are likely absent. This was found to be the case for the *Tritonia* homologue for the classic neurosecretory pre-pro-protein, FMRFamide [44,45]. Aligning the *Tritonia* FMRFamide protein sequence with homologues from fellow gastropods *Aplysia californica* and *Lymnaea stagnalis*, and the newly identified homologue from fellow Nudibranch *Melibe leonina*, for which we have also generated a CNS transcriptome (manuscript in preparation), reveals that *Tritonia* FMRFamide protein sequence is missing the N-terminal signal peptide sequence (Fig. 5). Interestingly, alignment of these four gastropod FMRF homologues reveals an interesting pattern where *Aplysia* FMRFamide has a greatly expanded number of FMRFamide repeats (i.e. 29) compared to *Lymnaea* (13), *Tritonia* (7), and *Melibe* (6) (Fig. 5).

### *Tritonia* genes involved in electrical signal propagation

**Fig 6:**
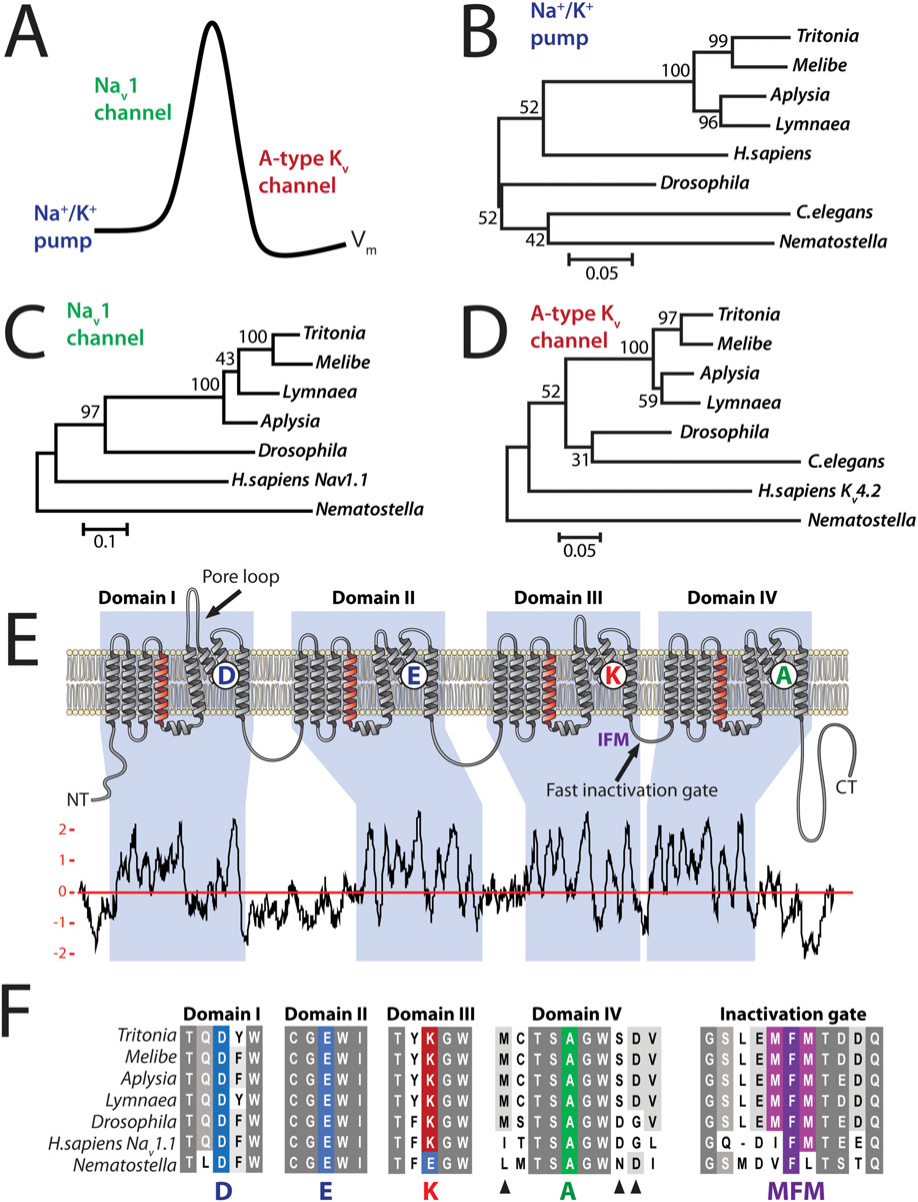
Phylogenetic analyses of *Tritonia* genes involved in propagating electrical signals. **A)** Depiction of three gene products with a central involvement in neuronal electrical signaling. The Na^+^/K^+^ ATPase pump actively transports three Na^+^ ions out of the cell for every 2 K^+^ ions pumped in, generating a charge differential across the cell membrane stored as electrical potential energy (resting membrane potential; RMP). The inside-negative RMP is a pre-requisite action potential propagation in neurons, where a propagating wave of depolarization mediated by Na^+^ influx through voltage-gated sodium (Na_v_) channels is countered by voltage-gated potassium (K_v_) channels that restore membrane polarization. **B)** A maximum likelihood phylogenetic tree based on MUSCLE alignment of Na^+^/K^+^ ATPase homologues from various species: *Tritonia diomedea* (extracted from CNS TSA), *Melibe leonina* (also a nudibranch; extracted from unpublished CNS TSA), *Aplysia californica* (GenBank accession no. XP_005093050.1), *Lymnaea stagnalis* (accession no. FX180758.1), *Drosophila melanogaster* (accession no. NP_996247.1), *Caenhorhabditis elegans* (accession no. NP_506269.1), *Homo sapiens* (accession no. NP_689509.1) and *Nematostella vectensis* (sequence ID NVE24413 from unpublished TSA [19]). **C)** Phylogenetic analysis of neuronal voltage-gated sodium (Na_v_1) channel homologues. GenBank accession numbers: *Lymnaea* FX180203.1; *Aplysia* AAC47457.1; *Drosophila* NP_001188603.1; *H.sapiens* BAC21101.1; and *Nematostella* (merge between TSA NVE7195 [19] and GenBank accession XP_001627761.1). **D)** Phylogeny for rapidly activating A-type potassium (K_v_) channels. GenBank accession numbers: *Lymnaea* FX185418.1; *Aplysia* XP_005091742.1; *Drosophila* NP_001097646.1; *C.elegans* NP_500975.2; *H.sapiens* AAD22053.1; and *Nematostella* (TSA NVE15334 [19]). **E)** Depiction of the expected topology for the *Tritonia* Na_v_1 channel, with 4 repeat domains (I to IV) each containing 6 transmembrane helices (S1 to S6, numbered left to right in each domain). The S4 helices are packed with alternating positively charged Lysine (K) or arginine (R) residues for voltage sensing. The pore-loops between S5 and S6 each project one of four key selectivity filter amino acids into the channel pore to define Na^+^ selectivity: aspartate (D); glutamate (E); Lysine (K); and alanine (A). The expected topology of *Tritonia* Na_v_1 is supported by hydrophobic peaks on a Kyte-Doolittle hydrophobicity plot (window size of 17; y-axis is the hydrophobicity score), that likely fold into transmembrane alpha-helices. **F)** Alignment of the pore-loop selectivity filter regions, and the Domain III-IV intracellular loop that houses the fast inactivation gate for Na_v_1 channels. The black arrows represent regions in the invertebrate channels that have diverged from vertebrate channels in positions known to affect TTX sensitivity [49].

**Fig 7:**
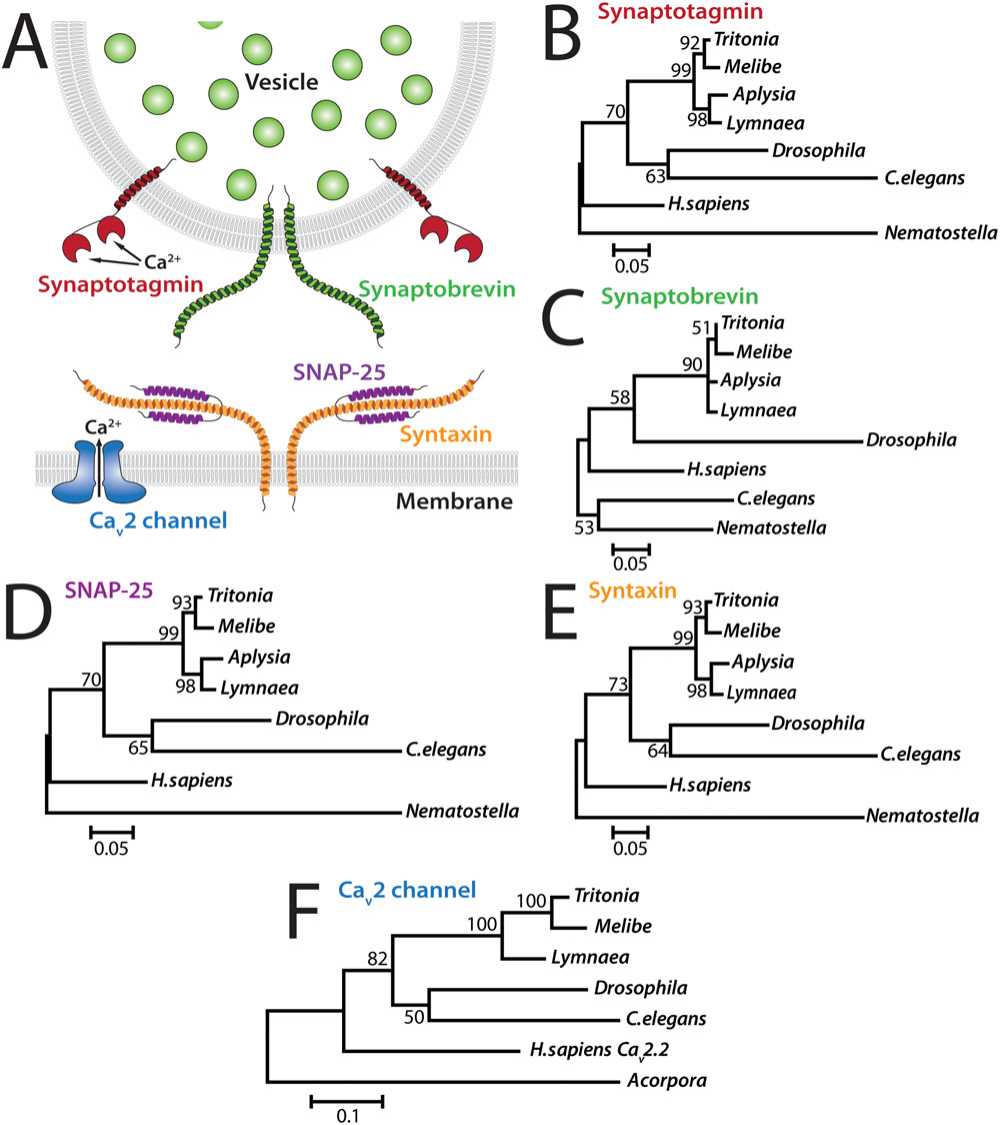
Phylogenetic analysis of *Tritonia* genes involved in synaptic vesicle release. **A)** Illustration of some core components of the synaptic vesicle release machinery, including pre-synaptic voltage-gated calcium (Ca_v_2) channels which convert membrane depolarizing signals such as action potential into inward Ca^2+^ flux, synaptobrevin, which binds Ca^2+^ to trigger assembly of the SNARE complex whose core components Synaptobrevin, SNAP-25, and Syntaxin mediate vesicle fusion and exocytosis [50]. **B)** Phylogenetic tree of Synaptotagmin homologues from various animal species. GenBank accession numbers: *Aplysia* NP_001191553.1; *Lymnaea* AAO83847.1; *Drosophila* NP_523460.2; *C.elegans* NP_001022129.1; *H.sapiens* NP_005630.1; and *Nematostella* TSA NVE15550. **C)** Phylogenetic tree of Synaptobrevin homologues. GenBank accession numbers: *Aplysia* NP_001191557.1; *Lymnaea* AAO83848.1; *Drosophila* NP_728646.2; *C.elegans* NP_504688.1; *H.sapiens* NP_055046.1; and *Nematostella* TSA NVE20764. **D)** Phylogenetic tree of SNAP-25 homologues. GenBank accession numbers: *Aplysia* NP_001191612.1; *Lymnaea* (merge between AAO83846.1 and FX183262.1); *Drosophila* NP_001036641.1; *C.elegans* NP_505641.2; *H.sapiens* BAD97337.1; and *Nematostella* TSA NVE19812. **E)** Phylogenetic tree of Syntaxin homologues. GenBank accession numbers: *Aplysia* AAA03566.1; *Lymnaea* AAO83845.1; *Drosophila* NP_524475.1; *C.elegans* NP_001022615.1; *H.sapiens* NP_004594.1; and *Nematostella* TSA NVE4110. **F)** Phylogenetic tree of Ca_v_2 voltage-gated calcium channel homologues. GenBank accession numbers: *Aplysia* XP_005113089.1; *Lymnaea* AAO83841.1; *Drosophila* NP_001245636.1; *C.elegans* NP_001123176.1; *H.sapiens* NP_000709.1; and cnidarian *Acropora millepora* JR974719.1.

**Fig 8:**
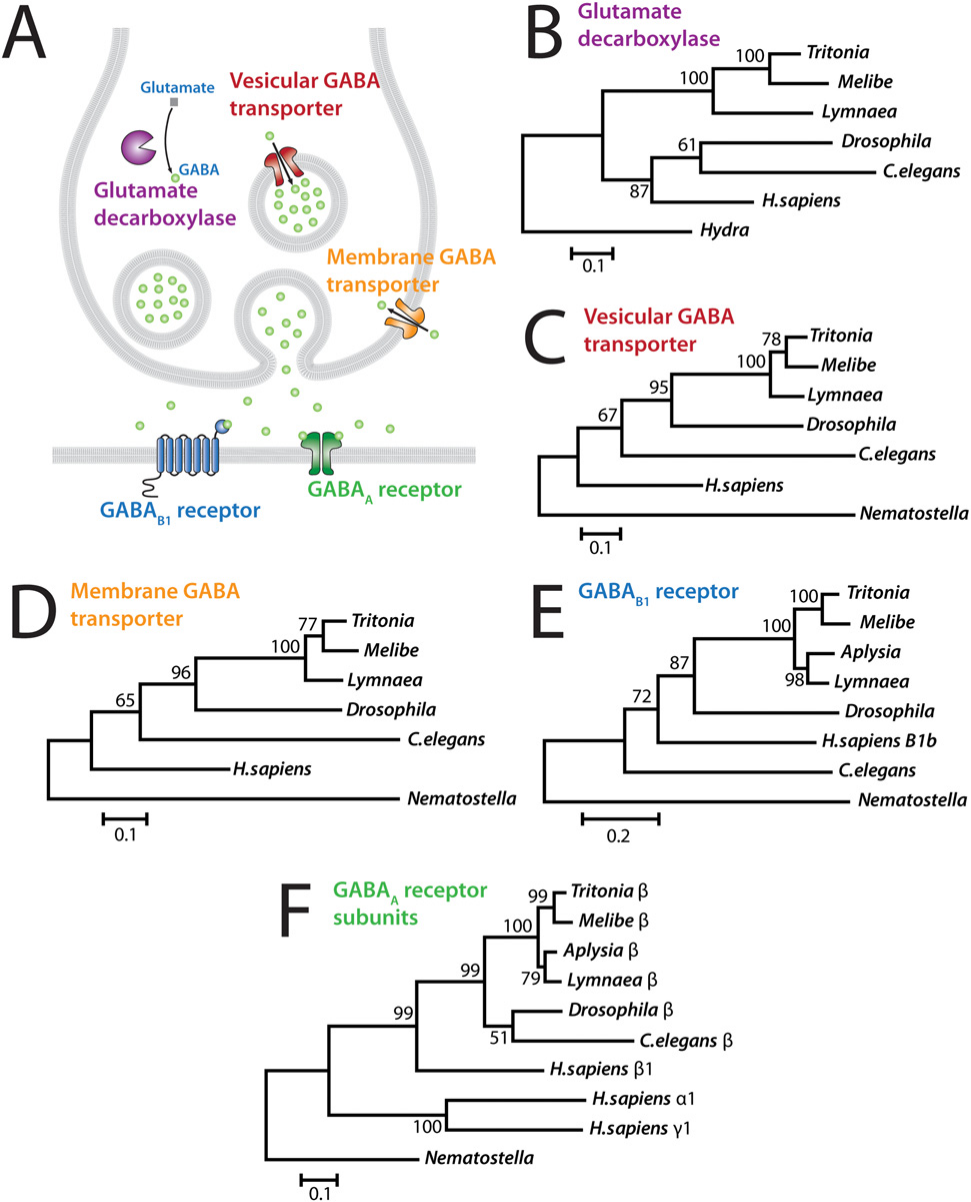
Phylogenetic analysis of *Tritonia* genes involved GABAergic neurotransmission. **A)** Illustration of some key genes involved in GABA-mediated neurotransmission, many of which were discovered in *C.elegans* [51]. The enzyme glutamate decarboxylase converts amino acid glutamate to neurotransmitter gamma aminobutyric acid (GABA). The vesicular GABA transporter fills presynaptic vesicles with GABA. The membrane GABA transporter clears secreted GABA from the synaptic cleft. The B1 subunit of the heterodimeric G-protein coupled GABA_B_ receptor mediates long-lasting post-synaptic responses to secreted GABA. The post-synaptic GABA_A_ receptor, a pentameric ligand-gated channel comprised of five subunits (2 α-subunits, 2 β-subunits, and 1 γ-subunit), conducts Cl^-^ ions upon ligand binding that depending on the chloride concentration gradient across the membrane can mediate depolarizing excitatory currents or hyperpolarizing inhibitory currents. **B-F)** Phylogenetic trees based on protein alignments with *Tritonia* homologues for (B) glutamate decarboxylase; (C) vesicular GABA transporter; (D) membrane GABA transporter; (E) the B1 subunit of heterodimeric G-protein coupled GABA_B_ receptor; and (F) various subunits of heteropentameric GABA_A_ GABA-gated Cl^-^ channels (i.e. β-subunits from protostome invertebrate channels; an unclassified subunit from *Nematostella*, and α-, β-, and γ-subunits from *H.sapiens*). GenBank accession numbers for **B)** glutamate decarboxylase: *Lymnaea* (FX184910.1 merged with FX210632.1); *Drosophila* NP_523914.2; *C.elegans* CAA21537.1; *H.sapiens* AAA62368.1; cnidarian *Hydra vulgaris* XP_002166171.2; **C)** vesicular GABA transporter: *Lymnaea* FX184418.1; *Drosophila* NP_610938.1; *C.elegans* P34579.2; *H.sapiens* NP_542119.1; *Nematostella* TSA NVE25975; **D)** membrane GABA transporter: *Lymnaea* FX184450.1; *Drosophila* AAR96151.1; *C.elegans* NP_505873.2; *H.sapiens* CAA38484.1; *Nematostella* TSA NVE14046; **E)** GABA_B1_ receptor: *Aplysia* XP_005092803.1; *Lymnaea* FX183485.1; *Drosophila* AAK13420.1; *C.elegans* ACE63490.1; *H.sapiens* NP_068703.1; *Nematostella* TSA NVE5469; and **F)** GABA_A_ receptor subunits: *Aplysia* β XP_005105426.1; *Lymnaea* β FX185014.1; *Drosophila* β NP_996469.1; *C.elegans* β AAN65376.1; *H.sapiens* β1 AK225167.1; *H.sapiens* α1 AK225167.1; *H.sapiens* γ1 P14867.3; and *Nematostella* (unclassified subunit) TSA NVE24726.

To highlight the abundance of genetic information available in the *Tritonia* TSA, we extracted *Tritonia* genes critical for action potential propagation (Fig. 6 and S3–S5 Figs.), synaptic vesicle release (Fig. 7 and S6–S10 Figs.), and one mode of synaptic neurotransmission (i.e. GABAergic; Fig. 8 and S11–S15 Figs.). We made alignments with homologous proteins from representative species of basal cniderians (*Nematostella vectensis*, *Acropora millepora*, or *Hydra vulgaris*), and the major division in bilaterian animals: 1) deuterostomes (*Homo sapiens*), and 2) protostome invertebrates (molluscs *Aplysia californica* and *Lymnaea stagnalis*; arthropod *Drosophila melanogaster*; and nematode *Caenorhabditis elegans*). We also included sequences from the TSA of Nudibranch *Melibe leonina*.

We selected three genes that are critical for propagating electrical signals in neurons: 1) the sodium-potassium ATPase (Na^+^/K^+^ pump), which polarizes the membrane by actively transporting Na^+^ and K^+^ ions out and into the cell respectively, 2) the neuronal voltage gated sodium channel (Na_v_1) which provides the major inward Na^+^ flux for action potential depolarization [46], and 3) the rapidly-activating A-type voltage-gated potassium channel (K_v_), which along with other K_v_ channels repolarizes the membrane to prime subsequent depolarizations (Fig. 6A). For all three genes, we identified transcripts containing full length ORFs. MUSCLE alignment (multiple sequence comparison by log-expectation) [47] of *in silico* translated protein sequences from *Tritonia* with those from other species (S3–S5 Figs.) and maximum likelihood phylogenetic tree reconstruction using the software MEGA 6.0 [48], revealed a mostly predictable pattern in the relatedness of *Tritonia* sequences to those of other animal phyla (Fig. 6B to D). With the exception of Na^+^/K^+^ pump, which is very highly conserved at the protein level making phylogeny difficult to infer (Fig. 6B and S3 Fig.), the *Tritonia* proteins are most similar to those from fellow nudibranch *Melibe*, followed by more distantly related gastropod molluscs *Aplysia* and *Lymnaea*. Other protostomes (*D. melanogaster* and *C. elegans*) show more similarity than deutersotomes (*H. sapiens*). Lastly, the basal metazoan with radial body symmetry, the cnidarian sea anemone *N. vectensis*, shows the least similarity.

The Na_v_1 channel alignment (S4 Fig.), and a Kyte-Doolittle Hydrophobicity plot of the *Tritonia* Na_v_1 channel protein sequence (Fig. 6E), reveal that *Tritonia* and in fact all molluscan Na_v_1 channels retain the structural hallmarks of voltage-gated sodium channels. These include 4-repeat domains (Domains I to IV), each containing six transmembrane alpha helices/segments (S1–S6). They have the canonical sodium channel selectivity filter motif DEKA, projecting one key amino acid into the pore to define sodium selectivity: aspartate (D), glutamate (E), lysine (K) and alanine (A), (Fig. 4E and F, S4 Fig.). The *Tritonia* and other molluscan Na_v_1 channels also retain strong homology in their domain III-domain IV linkers (Fig. 6E and F), which house the hinged-lid inactivation gate structure containing an IFM motif in vertebrates (MFM in the molluscan channels) that quickly occludes the pore upon channel opening for rapid inactivation [52,53]. Molluscan channels do differ from vertebrate channels in three loci of the domain IV pore-loop where mutations are known to reduce sensitivity to tetrodotoxin (TTX; Fig. 6F black arrows) [49]. This is interesting given that molluscan channels are generally less TTX-sensitive [54].

### *Tritonia* genes involved in neurotransmission

Voltage-gated N- and P/Q-type (i.e. Ca_v_2) calcium channels open in response to membrane potential depolarization caused by action potentials. Generally located at presynaptic terminals, they are responsible for the influx of Ca^2+^ that triggers secretion of vesicles via the actions of Ca^2+^-sensitive synaptotagmin and the SNARE complex proteins synaptobrevin, SNAP-25 and syntaxin [55] (Fig. 7A). We isolated full-length *Tritonia* homologues for these five genes (S6–S10 Figs.). Phylogenetic trees were created based on protein alignments with homologues from other species (Fig. 7 B to F, S6–S10 Figs.). Once again, the single-gene phylogenies reflect the species phylogenies, with *Melibe*, *Aplysia*, and *Lymnaea* proteins more similar than those from *Nematostella* and fellow cnidarian *Acropora millipora*. The only exception occurs for synaptobrevin, which is a particularly short and highly conserved protein (Fig. 7C and S7 Fig.).

Gamma aminobutyric acid (GABA), synthesized from the amino acid glutamate by glutamate decarboxylase (Fig. 8A), is a ubiquitous neurotransmitter that generally mediates inhibitory synaptic transmission. In several species of gastropod molluscs, GABAergic neurotransmission has been implicated in behaviors such respiration [56], olfaction [57,58], feeding [59–62] and reproduction [63], however its role specifically in Nudibranchs remains poorly understood [64]. Comparative immuno-localization of GABA in the CNS of several Nudibranch species, including *Tritonia*, revealed clusters of conserved GABAergic neurons in the cerebral and pedal ganglia [64]. Indeed, knowing the sequences of genes involved in different modes of synaptic transmission, including GABA, dopamine, glutamate, acetylcholine, and neuropeptides, provides avenues for exploring the CNS at the molecular level, such as localization of defined protein epitopes, or mRNA *in situ* hybridization. To illustrate, we extracted *Tritonia* homologues of several genes involved in GABAergic neurotransmission (Fig. 8A): Glutamate decarboxylase (Fig. 8B and S11 Fig.); the vesicular GABA transporter (Fig. 8C and S12 Fig.); the membrane GABA transporter (Fig. 8D and S13 Fig.); the postsynaptic metabotropic GABA_B1_ receptor (2,685 nt/894 aa; Fig. 8E and S14 Fig.); and a β subunit of the hetero-pentameric ionotropic GABA_A_ receptor (1,497 nt/498 aa; Fig. 8F and S15 Fig.). Alignment and phylogenetic analysis of the protein sequences again follows a predictive pattern (Fig. 8B to F), with the exception of the *C. elegans* metabotropic GABA_B1_ receptor, which falls between cnidarian *N. vectensis* and its bilaterian relatives (Fig. 8E). Also notable is that all of the invertebrate GABA_A_ receptor β subunits are more closely related to the human β than to α and γ subunits (Fig. 8F), corresponding to claims of shared ancestry for the pentameric subunits that make up ionotropic GABA_A_ receptors [65,66].

Unlike with other genes reported here, several of the *Tritonia* GABA-related genes were found to be partial/fragmented (i.e. Glutamate decarboxylase, the vesicular GABA transporter, and the membrane GABA transporter). However, in all cases we found additional overlapping sequences in the TSA that expanded the truncated ORFs, which for the membrane GABA transporter, yielded a full-length coding sequence (S13 Fig.).

## Discussion

### A CNS transcriptome for *Tritonia diomedea*

Here, we sequenced the CNS transcriptome of the nudibranch mollusc, *Tritonia diomedea* using Illumina sequencing and *de novo* transcript reconstruction. Our efforts generated a total of 185,546 sequences, which partitioned into 123,154 non-redundant components consisting of alternatively-spliced gene variants and closely related gene paralogues. *In silico* translation of TSA sequences revealed that a majority contain a complete protein-coding sequence (51.3%), however a substantial number are fragmented are missing the 5’ N-terminal coding sequence (19.6%), the 3’ C-terminal coding sequence (12.7%), or both (16.4%), as depicted in Fig. 1B and S1 Fig. Although in total 48.7% of genes are fragmented, many of these correspond to larger sequences that the Trinity assembler program was unable to resolve, probably due to low sequence coverage across gaps. As such, the percentage of fragmented coding sequences is artificially inflated relative to complete ones, since many of the former should combine into single transcripts.

To illustrate the utility of the *Tritonia* TSA for identifying gene sequences, we extracted those of genes involved in electrical excitability, synaptic vesicle release and in GABA neurotransmitter processing and post-synaptic detection. In most cases, we were able to derive full length protein sequences from the *Tritonia* TSA, and based on alignments with homologous proteins (S3–S15 Figs.), these did not contain any obvious assembly artifacts such as truncation or chimeric fusion with other sequences. In the few cases where selected genes were fragmented, we were often able to manually join several fragmented sequences by BLAST searching for missing 5’ or 3’ segments (see the section corresponding to Fig. 8). We predict, therefore, that as assembly programs improve, the same Illumina dataset should yield a much higher proportion of full-length ORFs. In the meantime, as it stands the TSA will serve as a useful tool for obtaining a multitude of gene sequences for studying their roles in neuronal connectivity, neuronal physiology and circuit dynamics in the *Tritonia* CNS.

PCR re-amplification and direct sequencing of the extracted *Tritonia* gene sequences (Figs. 6–8 and S3–S15 Figs.) was not performed. However, we have successfully done this for numerous other genes (not shown), indicating that the TSA represents *Tritonia* mRNA sequences with considerable accuracy. We also took extensive measures to post-filter contaminating gene sequences from five additional nudibranch species sequenced in parallel with *Tritonia* and are confident that most of these were removed (see methods). There may still be low level contamination from two mushroom and one moth species samples from other laboratories, which were also processed with our RNA. Without access to the corresponding raw Illumina data, we were unable to filter for them. Given the low degree of contamination that we did identify from other nudibranchs, we estimate that there are between one to four mushroom/moth contaminating unigene sequences per thousand TSA unigene sequences (i.e. < 0.4%).

### BLAST annotation of the *Tritonia* TSA

Through BLAST annotation, we found that the *Tritonia* TSA sequences and their corresponding *in silico* translated proteins find considerable homology in highly curated RefSeq and SwissProt protein databases. Of these, the invertebrate partition of RefSeq contained the most homologous sequences, as expected, with BLAST E-values below 1x10^-6^ (i.e. 16,179 and 12,123 unigene hits for the *Tritonia* TSA and predicted proteins respectively) (Fig. 2A). These numbers closely matched BLAST comparisons with a database of *Aplysia californica* mRNA sequences available on NCBI (16,096 for TSA, 11,793 for predicted proteins), which have been deposited over several years by multiple independent sequencing efforts. Interestingly, comparisons with TSA sequences from the recent *Lymnaea stagnalis* CNS transcriptome, which similar to the *Tritonia* TSA was generated via a single Illumina sequencing effort, contained almost as many BLAST hits as invertebrate RefSeq and *Aplysia* NCBI mRNAs (15,328 for TSA, 11,534 for predicted proteins) (Fig. 2B). Overall, our BLAST comparisons reveal that recent advances in sequencing technology and transcriptome assembly have vastly improved the amount of genetic information that can be extracted by a single sequencing effort. In fact, given the low cost for sequencing which is projected to continue dropping, it might be advantageous to re-sequence transcriptomes of species that were derived using the older Sanger sequencing method.

When considering the total and BHPC hits for *Tritonia* TSA BLAST comparisons, many more BLAST hits were found in the combined invertebrate mRNA databases (i.e. *Aplysia* mRNAs, *Lymnaea* TSA, *Biomphalaria* ESTs, and *Nematostella* TSA) than in the combined protein databases (Swiss-Prot and the three partitions of RefSeq: mammalian, non-mammalian vertebrates and invertebrates) (Fig. 2E). However, the majority of this enrichment occurs in the poorly stringent E-value range of 1x10^-1^ to 1x10^-6^, where hits are more likely to arise from alignment artifacts and not bona fide homology *per se* (Fig. 2). Correspondingly, protein prediction from the *Tritonia* TSA sequences with TransDecoder, which would filter out pseudogenes with lost reading frames, antisense RNA transcripts, non-coding RNA sequences, and contaminating genomic DNA sequences, produces markedly fewer alignments with poorer E-value scores between 1x10^-1^ and 1x10^-6^ (Fig. 2D). The elevated number of low scoring mRNA BLAST hits could also be partially accounted for by the fact that curated SwissProt and RefSeq protein databases should be devoid of pseudogenes and non-coding or genomic sequences, however these would be present in significant numbers in the mRNA databases used in our study. As such, these would be expected to undergo a greater degree of divergence relative to protein-coding genes, resulting in low-scoring BLAST hits.

### Gene ontology mapping

To evaluate the diversity of gene types contained within the *Tritonia* CNS TSA, we performed GO-mapping in the categories of molecular function, biological process and cellular component [22]. Interestingly, level 2 filtering of GO terms produced a distribution remarkably similar to those of fellow molluscs derived using a diversity of different sequencing technologies: *Lymnaea stagnalis* (Illumina sequencing of CNS [7]), *Aplysia californica* (Sanger sequencing of CNS [5]), *Octopus vulgaris* (Illumina sequencing of CNS [12]) and *Haliotis diversicolor* (Roche 454 sequencing of larvae [13]) (S2 Fig.). This makes sense, given that the GO term distributions are relative and would scale proportionately regardless of gene sequence number. Thus, the more noticeable impact for the different sequencing technologies is the total number of gene sequences that can be extracted in a given experiment [67]. Filtering GO terms by number of sequences, which provides more detailed information about GO mappings, revealed that a major portion of the mapped *Tritonia* TSA sequences and their associated proteins were assigned with functions of binding (i.e. to nucleotides, DNA, receptors and calcium) (Fig. 3A), which is to be expected of a neuronal transcriptome. To illustrate, binding of proteins to nucleotides, such as cyclic AMP and GTP/GDP, is highly prevalent in neurons for signal transduction. One obvious example is signalling by G-protein coupled receptors, that upon activation by an extracellular ligand, deploy GTP/GDP binding G-proteins to transduce an intracellular signal [68]. Similarly, Ca^2+^ binding proteins such as calmodulin are a prominent means of intracellular signal transduction in neurons [69]. Correspondingly, the most prominent GO term under biological process was signal transduction (Fig. 3B). A similar correspondence between molecular function and biological process can be seen for the large number of *Tritonia* TSA sequences associated with the molecular function of DNA binding (e.g. proteins that are transcriptional regulators and/or are involved in chromatin structure and modification) (Fig. 3A). In neurons, DNA binding is critical not only for cellular homeostasis but also for neuronal gene expression changes associated with learning and memory. In accordance, nuclear transcription is the fourth most abundant GO term in the category of biological process (Fig. 3B).

There was an unanticipated large number of genes associated with biological processes involving cellular proliferation and differentiation (Fig. 3B), given that the CNSs of juvenile/adult animals are expected to contain mostly non-proliferative terminally differentiated neurons. However, the molluscan CNS is interesting in that neurons are known to undergo massive genomic duplications [70,71] (i.e. endoreplication producing up to 200,000 times more DNA in a single neuron compared to a haploid sperm), where clearly, genes involved in pre-mitotic DNA replication (S phase of the cell cycle) would be recruited. Central neurons in gastropods have a broad range in soma diameter, with some massive cells reaching up to 1 mm diameter [5,72]. Genomic endoreplication is thought to facilitate exuberant cellular growth in eukaryotes [73], which in this case might allow these giant neurons to support more extensive post-synaptic innervation [72]. Also, the gastropod central nervous system is known to contain glia, and is surrounded by connective tissues containing myocytes, both of which might have contributed to the enrichment of proliferation/differentiation-associated genes in the *Tritonia* TSA.

### Neurosecretome analysis reveals that secreted proteins are amongst the most abundantly expressed genes in the *Tritonia* CNS

Quantification of assembled *Tritonia* gene sequences with the program RSEM revealed that neuronal secretory proteins are highly expressed in the brain. Indeed, the top three most highly-expressed genes with BLAST scores below 1x10^-4^ in either Swiss-Prot, RefSeqInv, RefSeqNMV, RefSeqMam or *Aplysia* mRNAs were neurosecretory peptides Cerebral peptide 1 [25,26], Pedal peptide 1 [27,28], and Abdominal ganglion neuropeptide R3–1 [29,30] (Table 3). Seven additional neuropeptides and one secreted protein also show up in the top thirty: Molluscan insulin related peptides 3 [31] and 7 [32], L5 neuropeptide [33], Neuroactive polyprotein R15 [34], PTSP-like peptide [5], small cardioactive peptide [35], sodium-influx-stimulating peptide [36], and the glia-secreted acetylcholine-binding protein [37,38]. This enrichment is quite staggering, especially when considering that there are only about 200 neuropeptide pre-pro-protein genes expected in the mollusc genome [74], and in the *Tritonia* brain ten of these are found within the top thirty most abundantly expressed genes. Gastropod neurons that are known to specialize in the secretion of neuropeptides are often enlarged, which is associated with genomic endoreplication to support higher level gene expression to support increased [72]. Indeed, the prominence of peptidergic signaling in gastropods, exemplified by the enrichment of neurosecretory peptide gene expression in the *Tritonia* CNS, have made gastropods useful models for understanding the roles of peptidergic signaling in neuronal physiology.

We generated a neurosecretome database from the *Tritonia* TSA, derived from a dataset of 9,832 completely non-redundant protein sequences (S3 Table). Our criteria for selection was similar to that recently used for generating a helminth secretome database [75], where sequences were filtered based on the presence of an N-terminal signal peptide (SignalP [39]), the absence of transmembrane helices (tmHMM [40]), and the absence of mitochondrial targeting signals (TargetP [41]) (Fig. 4A). The *Tritonia* neurosecretome differs from the helminth secretome in that we excluded predictions of non-classically secreted proteins that lack signal peptides identified using the program SecretomeP [43]. Using a criteria of no signal peptide, no transmembrane helices, and a SecretomeP NN-score ≥ 0.9, we identified 454 proteins. Amongst these we found the *Tritonia* homologue for MIP 3 (comp41988_c0), which shares significant BLAST homology with MIP 3 from *Lymnaea stagnalis* (Table 3). However, BLAST analysis of additional candidate secreted proteins revealed that many of them likely remain inside the cell, with functions relevant only to internal cellular sub-structures including the cytosol, the nucleus and the mitochondria. We therefore excluded the SecretomeP sequences from the neurosecretome database, but will likely expand this analysis once additional non-classically secreted protein sequences are available from other Nudibranch/gastropod species for cross-validation.

In accordance with our BLAST annotation of most abundantly expressed genes in Table 3, analysis of TPM expression levels of Trinity sub-component gene sequences reveals that secreted proteins undergo very high rates of transcription, accounting for as much as 26.68% of the sum TPM value for all non-redundant proteins combined (Fig. 4Bi). Perhaps more striking is that the sum TPM value for the 579 Trinity sub-components that correspond to neurosecretome proteins account for 9.93% of the sum TPM value for all 144,415 Trinity sub-components. We note that although the presented neurosecretome database is partly incomplete due to the absence of proteins lacking N-termini or non-classically secreted proteins, it nevertheless provides a valuable resource for studying peptidergic signalling in the mollusc brain, as well as other types of secreted proteins. Indeed, even with a preliminary analysis of two candidate neuropeptide pre-pro-proteins we were able to identify two interesting phenomena. First, alternative splicing serves to introduce novel putative neuropeptide repeats in the SCP protein that also changes the N-terminal sequence of the SCP-A peptide (Fig. 4C). Second, there is a pattern of duplication or loss of FMRFamide repeats in gastropod pre-pro-protein homologues, where the *Aplysia* gene has 29 repeats compared to 13 in *Lymnaea*, 7 in *Tritonia*, and 6 in *Melibe* (Fig. 5).

## Conclusions

*Tritonia* and other gastropods have highly accessible nervous systems with individually identifiable neurons whose connectivity and electrophysiological properties can be studied easily. *Tritonia* has played an important role in understanding how neural circuits are organized and how neurons co-ordinate behavior [76]. Recent studies have compared the neural circuits in *Tritonia* with those in other species [77]. However, until now, there has been little exploration at the molecular level, largely because of a lack of genetic sequence information. Indeed, if the diversity of molluscan systems is to remain viable for neuroscience research, accelerated progress needs to be made in techniques for molecular exploration. A clear first step is to generate a comprehensive dataset of expressed gene sequences for these species, which can subsequently be used for genetic manipulation (e.g. ectopic overexpression, gene silencing), functional characterization (expression and characterization of cloned genes), localization (*in situ* localization of mRNAs and proteins) and quantification (e.g. quantitative PCR, microarray). Fortunately, advances in next generation sequencing have made obtaining such mRNA sequence data relatively simple. The challenge now is to advance tools that permit using these sequences for manipulating experimental preparations.

## Methods

### RNA isolation and Illumina sequencing

Fifteen wild wild-caught *Tritonia diomedea* specimens with whole body masses between 49 and 205 grams were supplied by Living Elements (Vancouver, British Columbia, Canada). Total RNA was extracted from the central nervous system (cerebropleural, pedal and buccal ganglia) using an RNeasy Plus Universal Midi Kit (Qiagen). RNA integrity was confirmed by visualization of electrophoresed RNA on an ethidium bromide-stained agarose gel, as well as with a bioanalyzer using an Agilent RNA 6000 Pico Kit. RNA was then diluted to ~100 ng/μL in TE buffer (1 mM EDTA, 10 mM Tris pH 7.5), and shipped frozen on dry ice to Beckman Genomics for cDNA synthesis and Illumina sequencing. An automated pipeline was used by Beckman Genomics for sequencing, where first, mRNAs were isolated from total RNA using polyA capture, then cDNA was synthesized and PCR-amplified using proprietary primers that incorporate unique sequencing adaptors with barcodes for tracking Illumina reads from multiplexed samples run on a single sequencing lane. After paired-end sequencing of cDNAs on an Illumina HiSeq2500 (2x100 base pair reads), adaptor sequences were removed and low quality reads were filtered by Beckman Genomics prior to data delivery, producing a total of 133,156,930 paired reads (Table 1).

### *De novo* assembly, protein sequence prediction and transcript abundance estimation of the *Tritonia* TSA

The *Tritonia* CNS transcriptome shotgun assembly (TSA) was generated by *de novo* assembly of Illumina reads with the program Trinity [10,11] (release 2013–08–14). Prior to assembly, reads were trimmed with the program Sickle (https://github.com/najoshi/sickle) to remove 3’ and 5’ ends as well as entire reads with low quality scores. Trinity assembly, as well as all other analyses that employed high performance computers (HPCs), were done on a cluster of four IBM System x3850 X5 servers running Linux (VELA HPC system at Georgia State University). Parameters used for the Trinity assembly included a Jellyfish memory of 120 Gigabytes (-JM 120G), threaded on 48 processors (—CPU 48), and a FASTQ sequence type (—seqType fq). After assembly, the program TrinityStats.pl, which is part of the Trinity package, was used to generate metrics for the assembled transcript sequences (Table 1). Protein-coding open reading frames (ORFs) were predicted with the program TransDecoder [11] (http://transdecoder.sourceforge.net/), and custom MATLAB scripts were used to partition transcript and predicted peptide sequences into bins set by nucleotide/amino acid length. For TransDecoder peptides, a MATLAB script was used to further partition sequences within bins to count the numbers of full length, 5’ partial, 3’ partial, and internal fragmented ORFs (Fig. 1B, S1 Fig.). The relative expression levels of assembled gene sequences (i.e. Trinity sub-components) were estimated with the program RSEM (RNA-Seq by Expectation-Maximization) [24], which is bundled with the Trinity program. RSEM provides expression values as transcripts per million (TPM) (Table 3).

### BLAST annotation and gene ontology mapping with Blast2GO

Basic local alignment search tool (BLAST [18]) analyses were done with the 2.2.28 release of NCBI-BLAST+. The SwissProt and RefSeq protein databases used for BLAST comparison with the *Tritonia* TSA, as well the *Aplysia californica* NCBI mRNAs (taxonomy ID 6500), the *Lymnaea stagnalis* CNS TSA [7], the *Biomphalaria glabrata* NCBI ESTs (taxonomy ID 6526), and cnidarian *Nematostella vectensis* TSA [19] were all downloaded on February 22^nd^ 2014. For analysis of results, BLAST output files were converted to Microsoft Excel worksheets, and these were imported into a relational database using Microsoft SQL Server 2012. Custom SQL scripts were used to extract desired information the relational database. Gene ontology (GO) mapping was performed using NCBI-BLAST+, and the 2.7.1 version of Blast2GO [20] which infers GO terms based on BLAST data as well as InterProScan [21] annotations that can be run directly within Blast2GO.

### Filtering the *Tritonia* TSA to remove contaminating sequences

In parallel with *Tritonia*, we also sequenced the CNS transcriptomes of fellow Nudipleura *Melibe leonina*, *Dendronotus iris*, *Flabellina iodinea*, *Hermissenda crassicornis*, and *Pleurobranchaea californica* (manuscripts in preparation), from which several *Melibe* protein sequences were extracted for protein alignments and phylogenetic trees (Figs. 6–8 and S3–S15 Figs.). Of note, CNS RNA from these different species were processed and sequenced in parallel at the sequencing facility and we suspected that a low level of cross-contamination might have occurred. Furthermore, RNA from two fungal species and one moth species from other laboratories were processed at the sequencing facility along with our samples. To filter out contaminating nudibranch sequences, we performed nucleotide comparisons (BLASTn) between the *Tritonia* TSA and each of the other five TSAs. We then removed entire components from the *Tritonia* TSA from which at least one sequence produced a nucleotide alignment in another transcriptome of at least 50 nucleotides in length, a ≥ 97% identity, and where its RSEM TPM expression level value was ≤ 1/10^th^ of the TPM value of the aligned sequence from the other TSA. Unfortunately, without access to the sequence data for the fungi and the moth, we were unable to remove contamination originating from them. However, based on the number of sequences from the other nudibranchs, we expect only about 3 out of every 1000 components (i.e. unigenes) to arise from contamination. We have included RSEM TPM values in the FASTA headers of all *Tritonia* TSA sequences, which can be used to discern the likelihood of contamination, where contaminating sequences are expected to have very low TPM values.

### Generation of a neurosecretome database

TSA sequences were *in silico* translated with the program TransDecoder [11] and longest protein sequences for every Trinity sub-component, which were either complete (i.e. contained both a start methionine and a stop codon) or fragmented at the 3’ end (contained start methionine but lacked a stop codon) were extracted from the intermediate unfiltered TransDecoder output file called “longest_ORFs.pep”. Redundant sequences were then removed with the program CD-HIT-EST [78], producing a dataset of 9,832 completely non-redundant, unique protein sequences. These were then analyzed with SignalP version 4.1 [39], tmHMM version 2.0 [40], and TargetP version 1.1 [41]. A custom Microsoft SQL Server script was used to select protein sequences with predicted signal peptides with probability values ≥ 0.5, that lacked predicted mitochondrial targeting signals and that either lacked transmembrane helices altogether or contained a single transmembrane helix within the first 60 amino acids (the latter mostly represent signal peptides incorrectly predicted as transmembrane helices by tmHMM [40]). Annotations pertaining to each of the 579 predicted neurosecretory proteins are provided in S3 Table, and S1 Dataset contains the protein sequences in FASTA format.

### Identification of selected *Tritonia* protein sequences, protein alignments and phylogentic analyses

*Lymnaea stagnalis* and *Aplysia californica* protein sequences for genes of interest were manually collected online from NCBI or UniProtKB databases, and these sequences were used to find homologues in the *Tritonia diomedea* TSAs using BLAST. We used a similar strategy to identify homologous proteins from another Nudibranch mollusc, *Melibe leonina*, for which we have also generated a CNS TSA (manuscript is in preparation). The same set of *Lymnaea* and *Aplysia* sequences was used to find homologous proteins from other animal phyla, by BLAST searching through various NCBI and UniProtKB databases: *Homo sapiens*, *Drosophila melanogaster*, *Caenorhabditis elegans*, *Nematostella vectensis*, *Acropora millepora* and *Hydra vulgaris* (accession numbers for specific protein sequences are provided in the corresponding figure legends). In the case of *Nematostella vectensis*, protein sequences were obtained from a combination of NCBI/UniProtKB databases, as well as from the unpublished transcriptome assembly [19]. Proteins alignments (MUSCLE [47]) and phylogenetic trees (Maximum Likelihood [79]) were created using the Molecular Evolutionary Genetics Analysis (MEGA) software, version 6.0 [48]. Confidence values for phylogenetic tree branching were generated by the bootstrap method [80] (2000 replicates).

## Supporting Information

S1 Dataset FASTA file containing the sequences of the predicted *Tritonia* neurosecretome proteins. (TXT)

S1 Fig Length histogram of the longest representative peptide sequence per Trinity component. Extracting only the longest predicted protein sequence per Trinity component produces a decreasing distribution in the number of sequences as protein length increases (vertical axis), with an enrichment of complete ORFs (black bars) with respect to fragmented ones (5’ partial, blue bars; 3’ partial, red bars; and internal lacking both 5’ start sites and 3’ stop codons, yellow bars).
(EPS)

S2 Fig Combined graphs of gene ontology mapping for *Tritonia* TSA unigene sequences filtered by level 2 gene ontology terms. Percentage of *Tritonia* TSA contigs with assignments in the three major branches of gene ontology, molecular function, biological process, and cellular component. The distribution of gene ontology terms presented here are in clone agreements with the similarly and recently sequenced CNS transcriptome of freshwater snail *Lymnaea stagnalis* [7], with a major enrichment in terms for binding and catalytic activity (molecular function) and for cell and organelle (cellular component), and a broader distribution of terms for biological process.
(EPS)

S3 Fig MUSCLE protein alignment of Na^+^/K^+^ ATPase homologues from *Tritonia diomedea*, *Melibe leonina*, *Aplysia californica*, *Lymnaea stagnalis*, *Drosophila melanogaster*, *Caenorhabditis elegans*, *Homo sapiens* and *Nematostella vectensis*. (DOCX)

S4 Fig MUSCLE protein alignment of voltage-gated Na_v_ channel homologues from *Tritonia diomedea*, *Melibe leonina*, *Aplysia californica*, *Lymnaea stagnalis*, *Drosophila melanogaster*, *Homo sapiens* (Na_v_1.1 isotype) and *Nematostella vectensis*. (DOCX)

S5 Fig MUSCLE protein alignment of voltage-gated A-type K_v_ channel homologues from *Tritonia diomedea*, *Melibe leonina*, *Aplysia californica*, *Lymnaea stagnalis*, *Drosophila melanogaster*, *Caenorhabditis elegans*, *Homo sapiens* (K_v_4.2 isotype) and *Nematostella vectensis*. (DOCX)

S6 Fig MUSCLE protein alignment of synaptotagmin homologues from *Tritonia diomedea*, *Melibe leonina*, *Aplysia californica*, *Lymnaea stagnalis*, *Drosophila melanogaster*, *Caenorhabditis elegans*, *Homo sapiens* and *Nematostella vectensis*. (DOCX)

S7 Fig MUSCLE protein alignment of synaptobrevin homologues from *Tritonia diomedea*, *Melibe leonina*, *Aplysia californica*, *Lymnaea stagnalis*, *Drosophila melanogaster*, *Caenorhabditis elegans*, *Homo sapiens* and *Nematostella vectensis*. (DOCX)

S8 Fig MUSCLE protein alignment of SNAP-25 homologues from *Tritonia diomedea*, *Melibe leonina*, *Aplysia californica*, *Lymnaea stagnalis*, *Drosophila melanogaster*, *Caenorhabditis elegans*, *Homo sapiens* and *Nematostella vectensis*. (DOCX)

S9 Fig MUSCLE protein alignment of syntaxin homologues from *Tritonia diomedea*, *Melibe leonina*, *Aplysia californica*, *Lymnaea stagnalis*, *Drosophila melanogaster*, *Caenorhabditis elegans*, *Homo sapiens* and *Nematostella vectensis*. (DOCX)

S10 Fig MUSCLE protein alignment of voltage-gated Cav2 channel homologues from *Tritonia diomedea*, *Melibe leonina*, *Aplysia californica*, *Lymnaea stagnalis*, *Drosophila melanogaster*, *Caenorhabditis elegans*, *Homo sapiens* (Cav2.2 isotype) and *Nematostella vectensis*. (DOCX)

S11 Fig MUSCLE protein alignment of glutamate decarboxylase homologues from *Tritonia diomedea*, *Melibe leonina*, *Aplysia californica*, *Lymnaea stagnalis*, *Drosophila melanogaster*, *Caenorhabditis elegans*, *Homo sapiens* and Hydra vulgaris. (DOCX)

S12 Fig MUSCLE protein alignment of vesicular GABA transporter homologues from *Tritonia diomedea*, *Melibe leonina*, *Lymnaea stagnalis*, *Drosophila melanogaster*, *Caenorhabditis elegans*, *Homo sapiens* and *Nematostella vectensis*. (DOCX)

S13 Fig MUSCLE protein alignment of membrane GABA transporter homologues from *Tritonia diomedea*, *Melibe leonina*, *Lymnaea stagnalis*, *Drosophila melanogaster*, *Caenorhabditis elegans*, *Homo sapiens* and *Nematostella vectensis*. (DOCX)

S14 Fig MUSCLE protein alignment of metabotropic GABAB type Β1 homologues from *Tritonia diomedea*, *Melibe leonina*, *Aplysia californica*, *Lymnaea stagnalis*, *Drosophila melanogaster*, *Caenorhabditis elegans*, *Homo sapiens* (β1b subunit) and *Nematostella vectensis*. (DOCX)

S15 Fig MUSCLE protein alignment of ionotropic GABAA receptor homologues from *Tritonia diomedea*, *Melibe leonina*, *Aplysia californica*, *Lymnaea stagnalis*, *Drosophila melanogaster*, *Caenorhabditis elegans*, *Homo sapiens* (β1, α1 and γ1 subunits) and *Nematostella vectensis*. (DOCX)

S1 Table BLAST comparisons of the *Tritonia* TSA and predicted peptides with the Swiss-Prot and RefSeq protein databases. (XLSX)

S2 Table BLAST comparisons of the *Tritonia* TSA and predicted peptides with mRNA sequences from other invertebrates. (XLSX)

S3 Table Annotation details for the 579 predicted neurosecretory proteins from *Tritonia*. (XLSX)
