# Correction: Deep mRNA Sequencing of the *Tritonia diomedea* Brain Transcriptome Provides Access to Gene Homologues for Neuronal Excitability, Synaptic Transmission and Peptidergic Signalling

**DOI:** 10.1371/journal.pone.0123514

**Published:** 2015-03-30

**Authors:** The PLOS ONE Staff

In Fig. 4, “Generation of a neurosecretome database of the *Tritonia* brain”, some labels for species names are incorrectly omitted. Please see the Fig. 4 here.

**Fig 4:**
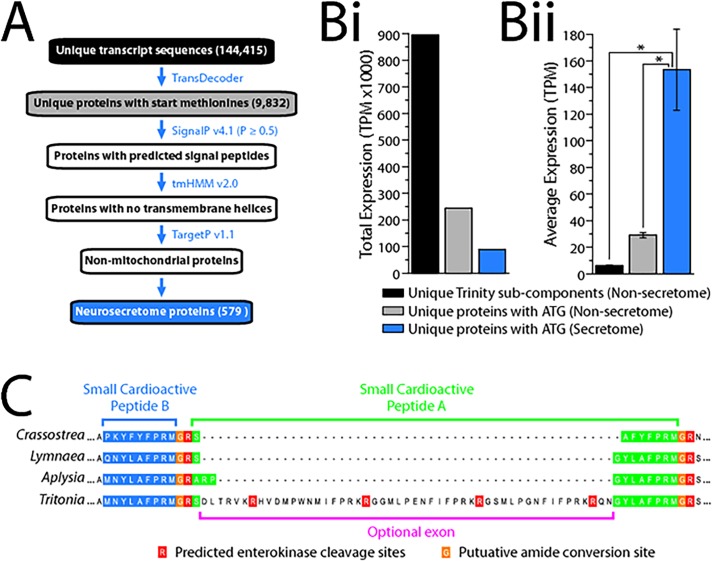
Generation of a neurosecretome database of the Tritonia brain. **A)** Schematic overview of the bioinformatics pipeline used to generate the neurosecretome database. TransDecoder translation of the 144,415 Trinity sub-component sequences produced 9,832 non-redundant proteins bearing N-terminal start methionines, and these were filtered for the presence of signal peptides (identified with the program SignalP), absence of transmembrane helices (with tmHMM), and absence of mitochondrial targeting signals (TargetP), producing a total of 579 predicted neurosecretome proteins. **Bi)** The sum TPM expression value for all neurosecretome proteins accounts for 26.67% of the sum TPM value for all 9,832 non-redundant proteins, and 9.93% of all sub-component gene sequences. **Bii)** The average TPM value for the neurosecretory proteins (153.38 ± 30.44 standard error) is 5.26-fold higher than the average of all non-redundant proteins (excluding neurosecretome proteins; 29.13 ± 2.00), and 24.67-fold higher than all sub-component gene sequences (also excluding neurosecretome proteins; 6.22 ± 0.40). The asterisks indicate probability values below 0.000 for one-way analysis of variance analysis comparing average TPM values. **C)** Section of a MUSCLE protein alignment of small cardioactive peptide pre-pro-protein homologues from bivalve mollusc *Crassostrea gigas* (UniProt accession no. Q5H7U9), and gastropod molluscs *Lymnaea stagnalis* (UniProt accession no. O97374), *Aplysia californica* (UniProt accession no. P09892) and *Tritonia diomedea* illustrates the position of a novel optional exon discovered in the *Tritonia* TSA that alters the N-terminal amino acid coding sequence of SCP-A and introduces three novel putative neuropeptide repeats, each ending with the residues IFPRK. Arginine (R) residues predicted by the NeuroPred algorithm as convertase cleavage sites are depicted with a red background, potential glycine residues that are likely converted to amides have an orange background, and the SCP-B and SCP-A peptide sequences have blue and green backgrounds, respectively.
