# Supplementary material for: Deep mRNA Sequencing of the Tritonia diomedea Brain Transcriptome Provides Access to Gene Homologues for Neuronal Excitability, Synaptic Transmission and Peptidergic Signalling
Source: PLoS One. 2015 Feb 26;10(2):e0118321. doi: 10.1371/journal.pone.0118321 (PMC4342343; doi:10.1371/journal.pone.0118321)
Supplement: S3 Fig — (DOCX) [file pone.0118321.s004.docx]

***T.diomedea* 1 ---MASSKVNKQGGPRAESYRYAVTP---------------SGDE--KKKKKKKGD--DLDELKQELEMDEHKIPIEELYTRLGVDPATGHSHDRAAEIL
*M.leonina* 1 -------------------------M---------------AKEK--KKKKKKGGD--DLDELKQELEMDEHKIPVEELYSRLGSNPTTGHSHERAKEIL
*A.californica* 1 ---MASSKVNKQG-PRADSYRYAVTP---------------SGEEKKKKKKKKGDD--DLDALKQELEMDEHKVPIEELYDRLEADPTNGHSPDRAKEIL
*L.stagnalis* 1 ---MASSKVNKQGGPRAESYRYAVTP---------------SGDEK-KSKKKKGEE--KLNELKQELDMDEHKIPIEELYERFGADPNNGHTPERAKEIL
*D.melanogaster* 1 -----------------------------------------------MPAKVNKKE--NLDDLKQELDIDFHKISPEELYQRFQTHPENGLSHAKAKENL
*C.elegans* 1 ------------------------------------------------MGKKDKKQ--ELHDLKQEVKMDEHIVPIEELVARLGTNLETGLTRQKAQEVL
*H.sapiens* 1 -----------------------MGD---------------KKDDKDSPKKNKGKERRDLDDLKKEVAMTEHKMSVEEVCRKYNTDCVQGLTHSKAQEIL
*N.vectensis* 1 MADSTELAALDEEYGRTDSYRVATSPLMGEQAAAPEGGKKAGEKKQGKRAKKKKDQKEKMEELKQELDVDWHRITVEELMTRLDTNVQTGLTDEEAAIRL

*T.diomedea* 79 NRDGPNALTPPKTTPEWIKFCKVLFTGFSLLLWIGAVLCYIAYSIEASQKDDVKGDNLYLGIVLTAVVVVTGIFSYYQEAKSSRIMDSFKTMVPQKALVI
*M.leonina* 57 LRDGPNELTPPKTTPEWIKFCKVLFTGFSLLLWIGAILCFIAYTIEATQKDNVAADNLYLGIVLTAVVVVTGIFSYYQEAKSSRIMDSFKTMVPQNATVI
*A.californica* 80 ARDGPNMLTPPKTTPEWIKFCKVLFTGFSLLLWIGAILCYIAYSIQASQQENPPGDNLYLGIVLTAVVVVTGCFSYYQEAKSSRIMDSFKNMVPQYAIVV
*L.stagnalis* 80 ARDGPNMLTPPKTTPEWVKFCKVLFTGFSLLLWIGAILCYIAYSIQASQSEDPPGDNLYLGIVLTAVVVVTGIFSYYQEAKSSRIMDSFKNMVPQYAVVI
*D.melanogaster* 52 ERDGPNALTPPKQTPEWVKFCKNLFGGFAMLLWIGAILCFVAYSIQASTSEEPADDNLYLGIVLSAVVIVTGIFSYYQESKSSKIMESFKNMVPQFATVI
*C.elegans* 51 AKNGPNALSPPETTPEWIKFCKNLFGGFAMLLWVGAILCYIAYSVDYFTMEYPSKDNLYLGIVLMTVVVITGVFQYYQESKSSKIMDSFKNMVPTFALVH
*H.sapiens* 63 ARDGPNALTPPPTTPEWVKFCRQLFGGFSILLWIGAILCFLAYGIQAGTEDDPSGDNLYLGIVLAAVVIITGCFSYYQEAKSSKIMESFKNMVPQQALVI
*N.vectensis* 101 KRDGPNALTPPPTTPEWVKFMKQMFGGFAMLLWIGAILCFIAQGIMEATEEEPLRDNLYLGIVLAFVVIVTGIFSYYQESKSSKIMESFKNLVPQEANVL

*T.diomedea* 179 RKGEPNTIDVEHIVMGDIVEVKFGDRVPADMRIISAHGFKVDNSSLTGESEPQTRTPEFTHENPLETRNLAFFSTNCVEGTARGIVVRIGDRSVMGRIAN
*M.leonina* 157 RQGEPLSLRVEEIVMGDIVEVKFGDRVPADMRIVSAHGFKVDNSSLTGESEPQTRTFEFTHENPLETRNLAFFSTNCVEGTARGIVVRIGDQSVMGRIAN
*A.californica* 180 RGSQKLSVRAEELVLGDIIEVKFGDRVPADMRVISAHGFKVDNSSLTGESEPQSRTAEFTHENPLETRNLAFFSTNAVEGTARGIVVKIGDQSVMGRIAN
*L.stagnalis* 180 RGGQLLSIRAEELVLGDIVEVKFGDRVPADIRVVSAHGFKVDNSSLTGESEPQSRTAEFTHENPLETRNLAFFSTNAVEGTCRGIVVKIGDNSVMGRIAN
*D.melanogaster* 152 REGEKLTLRAEDLVLGDVVEVKFGDRIPADIRIIEARNFKVDNSSLTGESEPQSRGAEFTHENPLETKNLAFFSTNAVEGTAKGVVISCGDHTVMGRIAG
*C.elegans* 151 RDGQKQQVKTEELVVGDIVEVKGGDRVPADLRVVSAFGFKVDNSSLTGESEPQSRSPDCTNENPLETRNIAFFSTNAVEGTAKGIVIYTGDNTVMGRIAH
*H.sapiens* 163 REGEKMQVNAEEVVVGDLVEIKGGDRVPADLRIISAHGCKVDNSSLTGESEPQTRSPDCTHDNPLETRNITFFSTNCVEGTARGVVVATGDRTVMGRIAT
*N.vectensis* 201 RSGGRKTVGAENLVIGDVVSVKGGDRLPGDIRILECKSFKVDNSSLTGESEPQSRGPDCTHENPLETRNLAFFSTYALEGNATGVVVQTGDNTVMGRIAN

*T.diomedea* 279 LASGLEVGDTPIHKEIDHFIHIITSVAVFLGVTFFIIAFILGYFWLDAVIFLIGIIVANVPEGLLATVTVCLTLTAKRMAKKNCLVKNLEAVETLGSTST
*M.*leonina 257 LASGLEVGDTPIAKEIDHFIHIITSVAVFLGVTFFIIAFILGYFWLNAVIFLIGIIVANVPEGLLATVTVCLTLTAKRMAKKNCLVKNLEAVETLGSTST
*A.californica* 280 LASGLEVNETPIAKEIAHFIHIITGVAVFLGVTFFIIAFILGYFWLDAVIFLIGIIVANVPEGLLATVTVCLTLTAKRMAKKNCLVKNLEAVETLGSTST
*L.*stagnalis 280 LASGLEVGETPIAKEIAHFIHIITGVAVFLGVTFFIIAFILGYFWLDAVIFLIGIIVANVPEGLLATVTVCLTLTAKRMAKKNCLVKNLEAVETLGSTST
*D.melanogaster* 252 LASGLDTGETPIAKEIHHFIHLITGVAVFLGVTFFVIAFILGYHWLDAVIFLIGIIVANVPEGLLATVTVCLTLTAKRMASKNCLVKNLEAVETLGSTST
*C.elegans* 251 LASGLDTGMTPIAREIEHFIHLITGVAVFLGISFFIIAFILGYHWLTAVVFLIGIIVANVPEGLIATVTVCLTLTAKRMASKNCLVKNLEAVETLGSTST
*H.sapiens* 263 LASGLEVGKTPIAIEIEHFIQLITGVAVFLGVSFFILSLILGYTWLEAVIFLIGIIVANVPEGLLATVTVCLTLTAKRMARKNCLVKNLEAVETLGSTST
*N.vectensis* 301 LASGLGSGKTPIAVEIEHFIHIITGVAVFLGVTFFIIAFILKYKWLEAVIFLIGIIVANVPEGLLATVTVCLTLTAKRMASKNCLVKNLEAVETLGSTST

*T.diomedea* 379 ICSDKTGTLTQNRMTVAHMWFDGRIVEADTSDDQCNAVYSASDPTWKSLARIAMLCNHAEFKGGQENVAVLKRECKGDASESALLKCAELSIGKVMEFRK
*M.leonina* 357 ICSDKTGTLTQNRMTVAHMWFDGRILEADTSDEQRNAKYSASDPTWKSLARIAMLCNRAEFRGGQDNVPVLKRECNGDASESALLKCVELSIGKVTEYRR
*A.californica* 380 ICSDKTGTLTQNRMTVAHMWFDGRISEAETNEDQTTASYSSKDPTWMSLARIGMLCNRAEFKANQENVPVLKRECNGDASESALLKCVELNIGKVTEFRR
*L.stagnalis* 380 ICSDKTGTLTQNRMTVAHMWFDGRIIEADTSDDQSNASYSRNDPTWMSLARISMLCNRAEFKAGQENVPVLKRECNGDASESALLKCVELSIGKVTEFRR
*D.melanogaster* 352 ICSDKTGTLTQNRMTVAHMWFDNQIIEADTTEDQSGVQYDRTSPGFKALSRIATLCNRAEFKGGQDGVPILKKEVSGDASEAALLKCMELALGDVMNIRK
*C.elegans* 351 ICSDKTGTLTQNRMTVAHMWYDETIHECDTTETQTSQE-KRTGASFEALVRIASLCNRAEFKAGQQDTPILRRDCTGDASEIALLKFTELTQGNVIAVRE
*H.sapiens* 363 ICSDKTGTLTQNRMTVAHMWFDNQIHEADTTEDQSGTSFDKSSHTWVALSHIAGLCNRAVFKGGQDNIPVLKRDVAGDASESALLKCIELSSGSVKLMRE
*N.vectensis* 401 ICSDKTGTLTQNRMTVAHLWYDNNIVEADTSEDQKQASGEKKSTTWYALSRVATLCNRAEFKADQEDKPVLKRECTGDASESALLKYVELAIGNVISFRK

*T.diomedea* 479 RHVKVTEIPFNSTNKYQVSIHTTDD---------------PNDPRHMLVMKGAPERIMDRCSTIMMHGKDQPLDDNFREAFNAAYLELGGLGERVLGFCD
*M.leonina* 457 RNKKMVEIPFNSTNKYQVSIHETED---------------PNDPRFVLVMKGAPERIMDRCSSILMHGKDQPLDDNFREAFNAAYLELGGLGERVLGFCD
*A.californica* 480 RNKKIVEIPFNSTNKYQVSIHETED---------------PNDPSYLLVMKGAPERIMDRCSTILMHGKDQPLDDNFREAFNAAYLELGGLGERVLGFCD
*L.stagnalis* 480 RNKKIVEIPFNSTNKYQVSIHETED---------------PNDPSYLLVMKGAPERIMDRCSTILMHGKEQPLDDNFREAFNSAYLELGGLGERVLGFCD
*D.melanogaster* 452 RNKKIAEVPFNSTNKYQVSIHETED---------------TNDPRYLLVMKGAPERILERCSTIFINGKEKVLDEEMKEAFNNAYMELGGLGERVLGFCD
*C.elegans* 450 KNKKIAEIPFNSTNKYQVSIHDNGD-------------------HYLLVMKGAPERILDVCSTIFLNGKESELTDKLREDFNTAYLELGGMGERVLGFCD
*H.sapiens* 463 RNKKVAEIPFNSTNKYQLSIHETED---------------PNDNRYLLVMKGAPERILDRCSTILLQGKEQPLDEEMKEAFQNAYLELGGLGERVLGFCH
*N.vectensis* 501 NHKKVCEIPFNSTNKYQVSIHEIPDEPEHDIEEGSDVDEREKNSKHILVMKGAPERILDRCSTILVNGKEQALDDKEKENFNQAYLDLGGLGERVLGFCH

*T.diomedea* 564 YTLPIEEFPPNFAFDPEG-PNFPLTGLRFVGLMSMIDPPRAAVPDAVGKCRSAGIKVIMVTGDHPITAKAIAKGVGIISEGSKTVEDIAAERGQPVEEVD
*M.leonina* 542 FVLPIEEFPPDFEFDPEG-PNFPLTGLRFVGLMSMIDPPRAAVPDAVGKCRSAGIKVIMVTGDHPITAKAIAKGVGIISEKSKTVEDIAAERGVPVEEVD
*A.californica* 565 YNLPIDEFPPNFEFDAEG-PNFPITGLRFVGLMSMIDPPRAAVPDAVGKCRSAGIKVIMVTGDHPITAKAIAKGVGIISEGSKTVEDLAAERSVPVEEVD
*L.stagnalis* 565 YLLPSAEFPPNYQFDPEG-PNFPITGLRFVGLMSMIDPPRAAVPDAVGKCRSAGIKVIMVTGDHPITAKAIAKGVGIISEGSKTVEDIAAERSIPVEEVD
*D.melanogaster* 537 FMLPSDKYPNGFKFNTDD-INFPIDNLRFVGLMSMIDPPRAAVPDAVAKCRSAGIKVIMVTGDHPITAKAIAKSVGIISEGNETVEDIAQRLNIPVSEVN
*C.elegans* 531 FVLPADKFPKGFKFDVEE-VNFPLKNLRFVGLMSMIDPPRAAVPDAVAKCRSAGIKVVMVTGDHPITAKAIAKSVGIISDGTETVEDIAIRRGIPVEEVN
*H.sapiens* 548 YYLPEEQFPKGFAFDCDD-VNFTTDNLCFVGLMSMIDPPRAAVPDAVGKCRSAGIKVIMVTGDHPITAKAIAKGVGIISEGNETVEDIAARLNIPVSQVN
*N.vectensis* 601 FYLPTEQFPLGFEFDPEDNPNFPLEGLCFVGLMSMIDPPRAAVPDAVSKCRSAGIKVIMVTGDHPITAKAIAKGVGIISEGTETVEDIAERLNIPVEEVD

*T.diomedea* 663 PREAKAAVIHGGDLRDMTPAQIDEILINHGEIVFARTSPQQKLIIVEGCQRQGQIVAVTGDGVNDSPALKKADIGVAMGIAGSDVSKQAADMILLDDNFA
*M.leonina* 641 PRDAKAAVIHGGDLRDMTPAQIDEILINHSEIVFARTSPQQKLIIVEGCQRQGQIVAVTGDGVNDSPALKKADIGVAMGIAGSDVSKQAADMILLDDNFA
*A.californica* 664 PREAKAAVIHGGDLRDMTPAQIDEILINHAEIVFARTSPQQKLIIVEGCQRQGQIVAVTGDGVNDSPALKKADIGVAMGIAGSDVSKQAADMILLDDNFA
*L.stagnalis* 664 PREAKAAVIHGGDLRDMTPAQIDEILINHAEIVFARTSPQQKLIIVEGCQRQGQIVAVTGDGVNDSPALKRADIGIAMGIAGSDVSKQAADMILLDDNFA
*D.melanogaster* 636 PREAKAAVVHGAELRDVSSDQLDEILRYHTEIVFARTSPQQKLIIVEGCQRMGAIVAVTGDGVNDSPALKKADIGVAMGIAGSDVSKQAADMILLDDNFA
*C.elegans* 630 PREAKAAVIHGSDLREMSEDQLAEIIKYHSEIVFARTSPQQKLMIVEGFQKQGQIVAVTGDGVNDSPALKKADIGVAMGIAGSDVSKQAADMILLDDNFA
*H.sapiens* 647 PRDAKACVIHGTDLKDFTSEQIDEILQNHTEIVFARTSPQQKLIIVEGCQRQGAIVAVTGDGVNDSPALKKADIGVAMGIAGSDVSKQAADMILLDDNFA
*N.vectensis* 701 ETKAKAIVVHGSQLKDYDQEKIDGILCNHTEIVFARTSPQQKLIIVEGCQRMGAIVAVTGDGVNDSPALKKADIGVAMGIAGSDVSKQAADMILLDDNFA

*T.diomedea* 763 SIVTGVEEGRLIFDNLKKSIAYTLTSNIPEISPFLLFILADIPLPLGTITILCIDLGTDMVPAISLAYEQAELDIMKRSPRNPVTDKLVNERLISMAYGQ
*M.leonina* 741 SIVTGVEEGRLIFDNLKKSIAYTLTSNIPEISPFLLFILADIPLPLGTITILCIDLGTDMVPAISLAYEQAEQDIMKRLPRNPVTDKLVNERLISMAYGQ
*A.californica* 764 SIVTGVEEGRLIFDNLKKSIAYTLTSNIPEISPFLLFILADIPLPLGTITILCIDLGTDMVPAISLAYEQAELDIMKRAPRNPFTDKLVNERLISMAYGQ
*L.stagnalis* 764 SIVTGVEEGRLIFDNLKKSIAYTLTSNIPEISPFLLFILADIPLPLGTITILCIDLGTDMVPAISLAYEQAELDIMKRLPRNPLKDKLVNDRLISMAYGQ
*D.melanogaster* 736 SIVTGVEEGRLIFDNLKKSIAYTLTSNIPEISPFLAFILCDIPLPLGTVTILCIDLGTDMVPAISLAYETAESDIMKRQPRNPFQDKLVNERLISMAYGQ
*C.elegans* 730 SIVVGVEEGRLIFDNLKKSIAYTLTSNIPEISPFLTYILFGIPLPLGTVTILCIDLGTDMVPAISLAYEEAESDIMKRQPRDPIRDKLVNERLISLAYGQ
*H.sapiens* 747 SIVTGVEEGRLIFDNLKKSIAYTLTSNIPEITPFLLFIMANIPLPLGTITILCIDLGTDMVPAISLAYEAAESDIMKRQPRNPRTDKLVNERLISMAYGQ
*N.vectensis* 801 SIVTGVEEGRLIFDNLKKSIAYTLTSNIPEISPFLMFIILDIPLPLGTVTILCIDLGTDMVPAISLAYEHAENDIMKRQPRDPINDKLVNERLIAMAYGQ

*T.diomedea* 863 IGMIQATAGFFTYFVIMGENGFWMSTLLGLRKQWDAPGVNDLEDSYGQEWTYTQRKKLEYTCHTGFFVSIVIVQWADLIICKTRRLSLFQQGMKNHRLTF
*M.leonina* 841 IGMIQASAGFFTYFVIMGENGFWMSKLLGLREQWDSITFNDVEDSYGQEWTYTQRKKLEYTCHTGFFVSIVIVQWADLIICKTRRLSLFQQGMKNHRLTF
*A.californica* 864 IGMIQASAGFFTYFVIMGENGFWMSTLLGIRKEWDSLGINDLEDSYGQEWTYSQRKKLEYTCHTAFFVAIVVVQWADLIICKTRRLSLFQQGMKNHRLTF
*L.stagnalis* 864 IGMIQATAGFFTYFVIMGENGFWMSRLLGIRKEWDSLGINDLEDSYGQEWTYSQRKKLEYTCHTAFFVSIVIVQWADLIICKTRRLSLFQQGMKNHRLTF
*D.melanogaster* 836 IGMIQAAAGFFVYFVIMAENGFLPKKLFGIRKMWDSKAVNDLTDSYGQEWTYRDRKTLEYTCHTAFFISIVVVQWADLIICKTRRNSIFQQGMRNWALNF
*C.elegans* 830 IGMIQASAGFFTYFWIMADNGFMPWDLYQLRAQWDSRAYNNVLDSYGQEWTYANRKILEYTCQTAYFVSIVVVQWADLIISKTRRNSLVQQGMSNWTLNF
*H.sapiens* 847 IGMIQALGGFFSYFVILAENGFLPGNLVGIRLNWDDRTVNDLEDSYGQQWTYEQRKVVEFTCHTAFFVSIVVVQWADLIICKTRRNSVFQQGMKNKILIF
*N.vectensis* 901 IGMIQASAGFFTYFVIMAENGFRPSLLFGLRRQWDDKYNHSVVDSYGQEWGYSQRKILEYTCHTAFFVSIVIVQWADLIICKTRRNSLFTQGMTNKFLNF

*T.diomedea* 963 GLFFETALAAFLTYCPGLDKGLRMQNLRFTWWLVALPYSLTIFVYDECRKLILRRNPQGFVERETYY
*M.leonina* 941 GLFFETALAAFMTYCPGLDKGLRMQNLRFTWWLVALPYSLAIFIYDECRKLILRKNPGGFVERETYY
*A.californica* 964 GLFFETALAAFLCYCPGLDKGLRMQPLRFTWWLTAIPYSLTIFIYDECRKLILRRNPGGFVERETYY
*L.stagnalis* 964 GLLFETVLAAFLCYCPGLDKGLRMQPLRFTWWLVALPYSLAIFIYDEVRKFILRRHPGGFVERETYY
*D.melanogaster* 936 GLVFETVLAAFLSYCPGMEKGLRMYPLKLVWWFPAIPFALAIFIYDETRRFYLRRNPGGWLEQETYY
*C.elegans* 930 GLVFETALAWFMCYCPGLDNGLRMYGLRFSWWFCALPFSILIFVYDEIRRFLIRRYPGGWVERETYY
*H.sapiens* 947 GLFEETALAAFLSYCPGMDVALRMYPLKPSWWFCAFPYSFLIFVYDEIRKLILRRNPGGWVEKETYY
*N.vectensis* 1001 GLFFETALAAFFSYTPGMSNGLRMYPLNWQWWFPAMPFSLIIWIYDECRRWILRRYPGCWLDKETYY**

**Figure S3. MUSCLE protein alignment of Na+/K+ ATPase homologues from *Tritonia diomedea*, *Melibe leonina*, *Aplysia californica*, *Lymnaea stagnalis*, *Drosophila melanogaster*, *Caenorhabditis elegans*, *Homo sapiens* and *Nematostella vectensis*.**
