# Supplementary material for: Deep mRNA Sequencing of the Tritonia diomedea Brain Transcriptome Provides Access to Gene Homologues for Neuronal Excitability, Synaptic Transmission and Peptidergic Signalling
Source: PLoS One. 2015 Feb 26;10(2):e0118321. doi: 10.1371/journal.pone.0118321 (PMC4342343; doi:10.1371/journal.pone.0118321)
Supplement: S4 Fig — (DOCX) [file pone.0118321.s005.docx]

*T.diomedea* 1 MDVELVEWTP-----FRLFTRESLFNIERRI-AEEEAAKHAENAQAESD----DDDDDLDEPSHHEENLKCNPKLEAGRKLPPSL-EDYP

*M.leonina* 1 MDEELTEWTP-----FRLFTRESLFNIERRI-AEEEAAKHAENVQAESD----DDDDDLDEPSHHEENLKCNPKLAAGRKLPPSL-EDYP

*A.californica* 1 MDHELAEWTP-----FRLFTRESLFTIDRRI-AEEEAAKHAEKAPPESD----DDDEELDEPSHHEENLKPNPKLEAGRKLPPSL-EDYP

L.stagnalis 1 MEEETVEWTP-----FRLFTRESLFNIERRI-AEEEAAKHAEKVKPESD----DEEDD-DDASQNEETLKPNPKLEAGRKLPPSL-EDYP

*D.melanogaster* 1 MTEDSDSISEEERSLFRPFTRESLVQIEQRIAAEHEKQKELERKRAEGEVIRYDDEDEDEGP-------QPDPTLEQGVPIPVRLQGSFP

*H.sapiens* Nav1.1 1 M--EQTVLVPPGPDSFNFFTRESLAAIERRI-AEEKA------KNPKPD----KKDDDENGP-------KPNSDLEAGKNLPFIY-GDIP

*N.vectensis* 1 ---------------------QSFLVIAGKF----------------------------------------------GKT----------

*T.diomedea* 80 KEYVAKPLEDLDEYYHNQKTFVVINKDKAIFRFSATDAVFLLTPFNPIRRVAIRILV--HPNF----NLLVMLTIIVNCVFMTLSW-SPP

*M.leonina* 80 KEYVAKPLEDLDEYYHNQKTFVVINKEKVIFRFSATNAVFILSPFNPIRRVAICILV--HPLF----SLLVMLTIITNCAFMTQIW-NPP

*A.californica* 80 REYVGKPLEDLDEFYHNQKTFVVLKKDKAIFRFSATDAIFLLSPFNPIRRIAIYILV--HPIF----SLLVMMTILVNCVFMAITSYTPP

L.stagnalis 79 REYIGKPLEDLDEFYHNQKTFVVLNKDKAIFRFSATNAIFLLSPFNPIRRTAIYILT--HPLF----SLTVMITIITNCVFMARAE-NPP

*D.melanogaster* 84 PELASTPLEDIDPYYSNVLTFVVVSKGKDIFRFSASKAMWMLDPFNPIRRVAIYILV--HPLF----SLFIITTILVNCILMIMP--TTP

*H.sapiens* Nav1.1 70 PEMVSEPLEDLDPYYINKKTFIVLNKGKAIFRFSATSALYILTPFNPLRKIAIKILV--HSLF----SMLIMCTILTNCVFMTMS--NPP

*N.vectensis* 14 ----------------------------QVYRFNRSKSLYLFGPENPIRQFSLKLITNQYPLQLHNRHMFVLLTILVNCVFLAMT--NPP

*T.diomedea* 163 GY---VEHIFTGIYTAEAVVKILSRGFILQPFTYLRDPWNWLDFFVISIAYMTNTIDVLGNLSALRTFRVFRALKTISVIPGLKTIVGAL

*M.leonina* 163 GY---VEHIFTGIYTVEAVVKILSRGFILQPFTYLRDPWNWLDFVVISIAYMTNTVPDLGNLSALRTFRVLRTLKTISVIPGLKTIVGAL

*A.californica* 164 AF---VEHIFLGIYTVEAVVKVLSRGFVLKPFTYLRDPWNWLDFFVISIAYMTMTVKSFGNLQALRTFRVLRALKTISVIPGLKTIVGAL

L.stagnalis 162 EY---VEHIFLGIYTVEAFIKTLSRGFILKPFTYLRDPWNWLDFFVISIAYMTMAIKSLGNLSALRTFRVLRALKTISVIPGLKTIVGAL

*D.melanogaster* 166 TV-ESTEVIFTGIYTFESAVKVMARGFILCPFTYLRDAWNWLDFVVIALAYVTMGID-LGNLAALRTFRVLRALKTVAIVPGLKTIVGAV

*H.sapiens* Nav1.1 152 DWTKNVEYTFTGIYTFESLIKIIARGFCLEDFTFLRDPWNWLDFTVITFAYVTEFVD-LGNVSALRTFRVLRALKTISVIPGLKTIVGAL

*N.vectensis* 74 EQ---PEYVFAAIYTIEMFCKIIAKGFALHRYAYLRDKWNWLDFIVVILGNVTISPD-VANLSGIPTFRVFRALRTISAVKGLKAMVNTL

*T.diomedea* 250 LEAVRRLRDVTILTVFMLSIFALIGMQLYTGSLRQKCVRNYELF-------LDQNHTME-------------ERIEFIW-NASNWLKDNF

*M.leonina* 250 LEAVSRLRDVTILTLFMLSIFALIGMQLYTGSLKQKCVKNYQLH-------LPINYTLE-------------EHDEFIH-NESNWKKDFF

*A.californica* 251 LEAVRRLRDVMILTVFVLSIFALIGMQLYSGALRQKCVLNPVPE-------LGTNITHD-------------EWNDWVN-NESHWQKDFY

L.stagnalis 249 LEAVRRLRDVMILTIFVLSIFALVGMQLYSGSLRHKCIKNYRIF-------YGANISHD-------------EWWEWVN-NESNWRTDHY

*D.melanogaster* 254 IESVKNLRDVIILTMFSLSVFALMGLQIYMGVLTQKCIKKFPLD-------GSWGNLTD-------------ENWDYHNRNSSNWYSEDE

*H.sapiens* Nav1.1 241 IQSVKKLSDVMILTVFCLSVFALIGLQLFMGNLRNKCIQWPPTNASLEEHSIEKNITVNYNGTLINETVFEFDWKSYIQDSRYHYFLEGF

*N.vectensis* 160 LVSMKMLWDVMVLTLFFICIFALIGMQLFIGELRNKCA----LP-------VPENLSV--------------PYRTYAS-NSSIWYLED-

*T.diomedea* 319 GTENQVCGEKQGAGKCGNESMNGTKIYICMADIGDNPNKDFTSFDNFGLALLSAFRLMTQDYWESLYHLILRAEGSLHFLYFVLVILLGS

*M.leonina* 319 GDEYMVCGNKTGAGGCGNSTRNGLPEYVCLPDIGPNPNGDFTSFDNFGLALLSAFRLMTQDFWESLYHLVLRAEGSLHLVYFVLVILFGS

*A.californica* 320 -DEWQVCGNGTGAGKCGNGTINGTAEWLCLPNIGQNPNHDFTSFDNFGMALLCAFRLMTQDFWESLYHLVLRAVGSAHCLYFVLVILLGS

L.stagnalis 318 -NEIQVCGNNSGAGQCGNNTFNGTAEYECLPGIGKNPNFDFTSFDNFGMALLCAFRLMTQDYWESLYHLVLRAEGMAHCLYFVLVILLGS

*D.melanogaster* 324 GISFPLCGNISGAGQCDDD-------YVCLQGFGPNPNYGYTSFDSFGWAFLSAFRLMTQDFWEDLYQLVLRAAGPWHMLFFIVIIFLGS

*H.sapiens* Nav1.1 331 -LDALLCGNSSDAGQCPEG-------YMCV-KAGRNPNYGYTSFDTFSWAFLSLFRLMTQDFWENLYQLTLRAAGKTYMIFFVLVIFLGS

*N.vectensis* 223 -NEPILCGNTTGSRTCPTN-------YTCLPHAGGNPNFGYTSFDHFGWALLTGFQLITLDFWENVYNNVIYTMGPWYVVYFAVVIFFGP

*T.diomedea* 409 FYLLNLILAIVAMSYEKTQK--QDQADAEE-------EAAERQEEETRKEALSL-------------------------MTKSPSNSSW-

*M.leonina* 409 FYLLNLILAIVAMSYDEQQK--QDKADAEE-------EAAERQEEETRKEVLSL-------------------------MTKSPSNSSW-

*A.californica* 409 FYLVNLILAIVAMSYDETQK--QDQADAEE-------EAAERQEEEARKEALSI-------------------------MTKSPSNSSWN

L.stagnalis 407 FYLVNLILAIVAMSYDEQQK--QDQADADE-------EAAERQEEEARKEAMSI-------------------------MSKSQSNSSW-

*D.melanogaster* 407 FYLVNLILAIVAMSYDELQKKAEEEEAAEE-------EAIREAEEAAAAKAAKLEERANAQAQAAADAAAAEEAALHPEMAKSPTYSCIS

*H.sapiens* Nav1.1 412 FYLINLILAVVAMAYEEQNQATLEEAEQKEAEFQQMIEQLKKQQEAAQQAATAT----------------------ASEHSREPSAAGRL

*N.vectensis* 305 FFLVNLVLAVVAASYENEVK------------------ASKMETEEERKE----------------------------------------

*T.diomedea* 464 NEYEAERAAMCDKPEEKERLSVTS-------------DHSVTSSHLKPSLKN--------------HKRHS------LSLPGSPYIHRTN

*M.leonina* 464 PEYEAERAAICDRAEEKERLSVTS-------------DHSLTSSHLKPSLKN--------------HKRHS------LSLPGSPYIHRTN

*A.californica* 465 NDFEAGVRTAGDKAEEKERLSLTS-------------DHSATSAHLKPSRLN--------------QKRHS------LSLPGSPYIHRRN

L.stagnalis 462 NEFDAGEKDLVDKPDEKERLSVTS-------------DQSMTSAHLKPSLLN--------------QKRHS------LSLPGSPYIHRRN

*D.melanogaster* 490 YELFVG-GEKGNDDNNKEKMSIRSVEVESESVSVIQRQPAPTTAHQATKVRK--------------VSTTS------LSLPGSPFNIRRG

*H.sapiens* Nav1.1 480 SDSSSEASKLSSKSAKERRNRRKKRKQKEQSGGEEKDEDEFQKSESEDSIRRKGFRFSIEGNRLTYEKRYSSPHQSLLSIRGSLFSPRRN

*N.vectensis* 337 ------------------------------------------------------------------QKRVA-----------SSYSVRRA

*T.diomedea* 521 SRGS-QYSWRKPVANTKRGVHCNDRQPLVHHTLENLP--LPFADDTSAVTPSSEDLCNYNYIRNMPNGRRFSFASQRRSGHPSDGNAGGR

*M.leonina* 521 SRGS-QYSWRKPVANTKRGVHCNDRQPLVHHTLENLP--LPYADDTSAVTPSSEDLCNYSFIRNMPNGRRFSFASQRRAGHP-DGSIGGR

*A.californica* 522 SRGS-QYSWRKPVPTAKRSPYCPDRQPLVHHTLENLP--LPFADDSAAVTPSSEDLCNFSFIRNMPNGRRFSFASQRR---P-DG--TGR

L.stagnalis 519 SKGS-QYSWRKPVTATKRGGHYTDRQPLVHHTLENLP--LPFADDSGAVTPSSEDLCNYSFVRNMPNGRRFSFASQKRSAGP-DS--GKQ

*D.melanogaster* 559 SRSSHKYTIRN--GRGRFGIPGSDRKPLVLSTYQDAQQHLPYADDSNAVTPMSEE-----------NGAIIV---------P---VYYGN

*H.sapiens* Nav1.1 570 SRTS-LFSFR---GRAKDVGSEND-----------------FADDEHSTFEDNESRRDSLFVPRRHGERRNSNLSQTS-----------R

*N.vectensis* 350 SILSIVY------GQGDVIPDEPNSKGLYD---------IPVVTCSEVTKSDEEALSVFQI---------------RQ-----------E

*T.diomedea* 608 PGSRRSSFTSNHSRASRTSCAS-QVDKSKMETLLNFKKGKVPDVVLDKSKLDDD-DSVSS-DSGH------------FPEKNK--ASESN

*M.leonina* 607 PGSRRSSFTSNHSRASRTSRGS-QVDKSKMETLLNFKKGKVPDVVLDKSKLDDE-DSVSS-DSGH------------FPEKDK--ASESN

*A.californica* 603 SGSRRSSFASNHSRASRTSRGSGQGDRTKTQTLLNFKKGKVPDVVLDKSKLDDDQDSVSSGGSGH------------CPEKDK--ASESN

L.stagnalis 603 TGSRRSSFASNHSRTSRTSRGSQQADRSKMETLLNFKKGKVPDVVLDKSKLDDDADSLSS-GSGH------------CPEKDK--TSESN

*D.melanogaster* 624 LGSRHSSYTSHQSRISYTSHGDLLGGMAVMGVSTMTKESKLRNRNTRNQSVGATNGGTTCLDTNHKLDHRDYEIGLECTDEAGKIKHHDN

*H.sapiens* Nav1.1 628 SSRMLAVFPANGKMHSTVDCNGVVSLVGGPSVPTSPVGQLLPEVIIDKPATDDNGTTTETEMRKR--------------RSSS--FHVSM

*N.vectensis* 399 SGIQNQNLTLHQNWIGSRQPSSVTLPSLKPEHALHMNGSQ-------RSNLNHTSDIIGS-------------------QKSN--LNNAS

*T.diomedea* 681 PFLSHSPGGHNVEMKDVMVLKDILDQASG-HRRSFVSMASI----------QQKTMKDTVWK-------YFCVWDCNPQFQKVQRLASLF

*M.leonina* 680 PFLSHSSGGHNVEMKDVMVLKDILDQASG-HRRSFVSMASI----------QQKTMKDTVWK-------YFCVWDCHPYFTQLQHYVSLF

*A.californica* 679 PFLSHSPGGPNVEMKDVMVLKDILDQASG-HRRSFVSMTSI----------HQRTMKDIMWK-------YFCTWDCHPNFQKLQRLVSLF

L.stagnalis 678 PFLGNTPGGPNVEMKDVMVLKDILDQASG-HRRSFVSMASI----------QQKTMKDIMWK-------YFCTWDCNPNFQKLQRLVSLF

*D.melanogaster* 714 PFIEPVQTQTVVDMKDVMVLNDIIEQAAGRHSRASDRGVSVYYFPTEDDDEDGPTFKDKALEVILKGIDVFCVWDCCWVWLKFQEWVSLI

*H.sapiens* Nav1.1 702 DFLED-PSQRQRAMSIASILTNTVEELE-----------------------ESRQKCPPCWY---KFSNIFLIWDCSPYWLKVKHVVNLV

*N.vectensis* 461 QIISSHKSSLNLSVKK-----------------------------------EEKKRSSIMAR----------------RWKRFRRRLLKF

*T.diomedea* 753 IMDAFVDLFITICILVNTFFMAMDQYDMDKDLKRMSGQANEVFTAIFAIEAFLKILALSPVNYFKDGWNIFDSIIVALSLMELCLEE---

*M.leonina* 752 IMDAFVDLFINISILVNTSFMALEQHDMDPDLARISTIANEVFTSIFAIEAFLKILALSPVNYFKEGWNIFDSLIVALSLMELSLKEL--

*A.californica* 751 IMDAFVDLFITICILVNTAFMAMEHYDMEDDLKAVSNAANLVFTAIFAVEAFLKILALSPVIYFKDGWNIFDSIIVALSLMELSMTKL--

L.stagnalis 750 IMDAFVDLFITVCIVVNTLFMAMDHYNMDKNLQDISSQANEVFTAIFAAEAFLKILAMSPVVYFKDGWNIFDSLIVALSLMELSMKEL--

*D.melanogaster* 804 VFDPFVELFITLCIVVNTMFMAMDHHDMNKEMERVLKSGNYFFTATFAIEATMKLMAMSPKYYFQEGWNIFDFIIVALSLLELGLEGV--

*H.sapiens* Nav1.1 765 VMDPFVDLAITICIVLNTLFMAMEHYPMTDHFNNVLTVGNLVFTGIFTAEMFLKIIAMDPYYYFQEGWNIFDGFIVTLSLVELGLANV--

*N.vectensis* 500 TNGKVMEIFIIVCILLNTLVMSIEHPRLEDPLLTVVNISNEVFTFIFLLEMILKLIALGFLGYIRVAWNIFDGIVVIISIVDFIVNKFVP

*T.diomedea* 840 ----SGLFSVLRAFRLLRVFKLAKSWRTLNMLISIVARTMSALGNLIIVLGIVIFIFAVMGQQLFSEDYV--EAAMVREG-PNKTFDVSK

*M.leonina* 840 ----PGGLSVLRAFRLLRVFKLAKSWRTLNMLISIVARTMGALGNLIIVLGMVIFIFAVMGQQLFSADYI--REATVDFGTPNSTFVLEL

*A.californica* 839 ----PGL-SVLRAFRLLRVFKLAKSWPTLNMLIAIVGRTMGALGNLIIVLGIIIFIFAVMGQQLFSSDYKTYEREIDAWG--NVTINKDK

L.stagnalis 838 ----PGL-SVLRAFRLLRVFKLAKSWPTLNMLIAIVARTMGALGNLIIVLAIVIFIFAVMGQQLFSTHYAIYLYKELDNG--TKVYDIDN

*D.melanogaster* 892 ----QGL-SVLRSFRLLRVFKLAKSWPTLNLLISIMGRTMGALGNLTFVLCIIIFIFAVMGMQLFGKNYH--DH-KDRFP-------DGD

*H.sapiens* Nav1.1 853 ----EGL-SVLRSFRLLRVFKLAKSWPTLNMLIKIIGNSVGALGNLTLVLAIIVFIFAVVGMQLFGKSYK--DCVCKIAS-------DCQ

*N.vectensis* 590 DAGGTGI-SVLRTFRLLRVLKLAKSWSTMNSLLATIGKSLGALGNLTVILAIIVYIFAVMGMQLLGNSYT-----PDKFG--------GS

*T.diomedea* 923 MPRWNFSDFLHSFMIVFRVLCGEWIESMWGCYEVSGWS-CVPFFLLTYIIGNLVVLNLFLALLLSSFGTATL--SEAEDEPNKLAEAIDR

*M.leonina* 924 MPRWNFSDFAHSLMIVFRVLCGEWIESMWGCYSAAGMS-CVPFFLLTYIIGNLVVLSLFLALLLSSFGSESLTGSDGEGEPNKLGEAKNR

*A.californica* 922 MPRWNFNDFLHSFMIVFRVLCGEWIESMWGCYLVSGWA-CVPFFLLTYVVGNLVVLNLFLALLLSSFGSESLQRSESDDEPSKIAEAIDR

L.stagnalis 921 MPRWNFNDFLHSFMIVFRVLCGEWIESMWWCHKAAGWP-CVPFFLLTYIIGNLVVLNLFLALLLSSFGSESLSRSESADEPNKIAEAIDR

*D.melanogaster* 967 LPRWNFTDFMHSFMIVFRVLCGEWIESMWDCMYVGDVS-CIPFFLATVVIGNLVVLNLFLALLLSNFGSSSLSAPTADNDTNKIAEAFNR

*H.sapiens* Nav1.1 929 LPRWHMNDFFHSFLIVFRVLCGEWIETMWDCMEVAGQAMCLTVFMMVMVIGNLVVLNLFLALLLSSFSADNLAATDDDNEMNNLQIAVDR

*N.vectensis* 666 IPRWNFKDFPHSFMMIFRVLCGEWIEPLWDCMLATGPV-AMLLFVPAFILGNFIILNLFLALLLSSFA------SESGTQPKK-------

*T.diomedea* 1010 FKRFGGWVKVKMIVF---VKVKLQRKKNWRPPTVPLEQPDANGKEAATLSDRVV---------SSLEKTPDDIPDGAMLSRAGSICSAKK

*M.leonina* 1013 FIRFGRWVKVKTIVF---VKVKLQRKKNYRPPTGLDDQPDANGKEAATLSDRVV---------SSLEKTPDDAPDGAMLSRAGSVCSMKH

*A.californica* 1011 FKRFGNWVKVKIIVC---IKVKLQRQKNWRPP-PTTGQSEVNGKDPA-VVDGTV---------VSMEKTPDDFPDGPCCPAQAAICSAKK

L.stagnalis 1010 FKRFGNWVKVKIIVC---IKVKLQRQKNWRPSVPPSELPELNGKENA-FGDGTV---------IAMEKTPDDFPDGAMVSRAGSIYSTK-

*D.melanogaster* 1056 IGRFKSWVKRNIADCFKLIRNKLTNQISDQPSEHGDNELEL-GHDEI-LADGLIKKG-----IKEQTQLEVAIGDGMEFTIHGDMKNNKP

*H.sapiens* Nav1.1 1019 MHKGVAYVKRKIYEF---IQQSFIRKQKILDEIKPLD--DLNNKKDSCMSNHTTEIGKDLDYLKDVNGTTSGIGTGSSVEKYIIDESDYM

*N.vectensis* 742 -KKRKSFLSRMKNIM---ILMRLNKRTQVEPT---KGEGEVIGENVS---------------------------TGGEAEEEGVEQDSSL

*T.diomedea* 1088 E--------------LKSPTASH-----SGSSHCSS--CSSL-SENAHATTKIDLEAEHEINEVDVVYVK------------------EP

*M.leonina* 1091 E--------------LKSPSGSH-----SGSSHCSS--CSSL-SENAHTTTKIDLEGEQEINEVDVVYVK------------------EP

*A.californica* 1087 D--------------LKSPSGSHSN---SGSSHCSS--CSSL-SESAQ-TKKIDLEADHEINEVEIVYVK------------------EP

L.stagnalis 1086 D--------------LKSPLGSH-----SGSSHCSS--CSSL-SDSAQ-TKKIDLEGDHEINEVEIVYAK------------------EP

*D.melanogaster* 1139 KKSKYLNNATDDDTASINSYGSHKNRPFKDESHKGS--AETMEGEEKRDASKEDLGLDEELDEEGECEEGPLDGDIIIHAHDEDILDEYP

*H.sapiens* Nav1.1 1104 S-------------FINNPSLTVTVPIAVGESDFENLNTEDFSSESDLEESKEKLNESSSSSEGSTVDIGAPVEEQPVVEPEETL---EP

*N.vectensis* 798 E--------------LQP----------SGSTKKAV--CCCV----------IDLTDYP------------------------------V

*T.diomedea* 1138 DDCLCYSCTRRCPWCTKLEKKLIGRIWWKFRCVIYRLAEHKYFDTFIIVMILASSCALALEDAYLHEKPVLKDILEYLDKVFTVIFIVEM

*M.leonina* 1141 DDCFGYSCTKRCPWCLKLEKKLIGRMWWKFRCFFYQLAEHKYFDTFIIVMIVASSCALALEDAYLHEKPLLKDILEYMDRVFTAIFIIEM

*A.californica* 1138 DDCFCYMCTKRCPWCVKVEKSKIGRAWWAVRCFFYRLTENKYFDSFIIAMILASSCALALEDAYLHEKPILKEILEYLDKVFTAIFIIEM

L.stagnalis 1135 DDCFCYSLTKRCTWCLVIEKSPIGRAWWALRCFMYRLAEHRYFDTFIIVMILLSSCALALEDAYLHEKPLLKEILEYMDKVFTVIFIVEM

*D.melanogaster* 1227 ADCCPDSYYKKFPILAGDDDSPFWQGWGNLRLKTFQLIENKYFETAVITMILMSSLALALEDVHLPQRPILQDILYYMDRIFTVIFFLEM

*H.sapiens* Nav1.1 1178 EACFTEGCVQRFKCCQINVEEGRGKQWWNLRRTCFRIVEHNWFETFIVFMILLSSGALAFEDIYIDQRKTIKTMLEYADKVFTYIFILEM

*N.vectensis* 822 EPCFPTSW---CVCRLACMDSGFCAVWKVMRYKVRRLVEHKVFEGIILFLIAASSISLAFEDVYLDSKPTLKQVLQILNILFAVIFTVEM

*T.diomedea* 1228 IVKWFAFGFKVYFTDAWCWLDFSIVMLSIVMLIADTLEPADG-GGGGMGAMRSMRTLRALRPLRAVSRWEGMRVVVNALFKAIPSICHVL

*M.leonina* 1231 LIKWFAFGFKVYFTDAWCWLDFSIVMLSIVMLVAEQVQPADE-GGGGVGAMKSMRTLRALRPLRAVSRWEGMRVVVNALFKAIPSICNVL

*A.californica* 1228 LIKWLAFGFKTYFTDAWCWLDFTIVMLSIVMLVADLTASEE--GGGSMSWMKSMRTLRALRPLRAVSRWEGMRVVVNALFKAIPSICNVL

L.stagnalis 1225 LVKWFAFGFKTYFTDAWCWLDFCIVMLSIMMLMADMMASADGHGGGKMGAMRSIRTLRALRPLRAVSRWEGMRVVVNALFKAIPSICNVL

*D.melanogaster* 1317 LIKWLALGFKVYFTNAWCWLDFVIVMVSLINFVASLV------GAGGIQAFKTMRTLRALRPLRAMSRMQGMRVVVNALVQAIPSIFNVL

*H.sapiens* Nav1.1 1268 LLKWVAYGYQTYFTNAWCWLDFLIVDVSLVSLTANAL------GYSELGAIKSLRTLRALRPLRALSRFEGMRVVVNALLGAIPSIMNVL

*N.vectensis* 909 LLKWIGLGFKTYFTNPWNILDFVIVIVSLATIF----------GNDQIAFIRSLRTLRAFRPLRAISRFEGMKVVITSLLHAIPGIGNVL

*T.diomedea* 1317 MVLLVFWLIFGIMGVQLFNGKFYACYN--NGEKVTREEVPDRDTCF-----EKEYNWTNAQINFDNVIAAYLALFQVATYKGWIDIMNSA

*M.leonina* 1320 VVCLVFWLIFGIIGVQLFKGRFYACFN--MEERCSEEEVPDKETCF-----AKQYNWTNAMINFDNVIQAYLALLEIATYKGWIDIMNSA

*A.californica* 1316 LVCLVFWLIFGIMGVQLFNGKFHACRD-EKGDKYPREEVPNKTVCI-----DKGYNWTNAQINFDHVLSAYLALFQVATYKGWIDIMNNA

L.stagnalis 1315 LVCLVFWLIFGIMGVQLFNGKFHACVC-ENGTRCEPDVIPNRTVCE-----LQGYNWTNAQINFDNVIAAYLALFQVATYKGWVDIMNNA

*D.melanogaster* 1401 LVCLIFWLIFAIMGVQLFAGKYFKCED-MNGTKLSHEIIPNRNACE-----SENYTWVNSAMNFDHVGNAYLCLFQVATFKGWIQIMNDA

*H.sapiens* Nav1.1 1352 LVCLIFWLIFSIMGVNLFAGKFYHCINTTTGDRFDIEDVNNHTDCLKLIERNETARWKNVKVNFDNVGFGYLSLLQVATFKGWMDIMYAA

*N.vectensis* 989 LVCLMFWLIFSIMGVQIFGGKFGKCVD-EGGEKCPASVVPNKTVCL-----SNGYRWENSNINFDTVDQGFLALLQVATFEGWMEIMEDA

*T.diomedea* 1400 IDSTGIGNQPTREHSTYYYLFFVLFIIFGSFFTLNLFIGVIIENFNSQKRKAG--GSLEMFMTDDQKKYYNAMKRMQSKSPQKSIPRPKY

*M.leonina* 1403 IDSTGIDKQPIREYSLVYYIFFVLFIILGSFFTLNLFIGVIIENFNSQKKKAG--GSLEMFMTDDQKKYYNAMKRMQSKSPQKSIPRPKY

*A.californica* 1400 IDSTEIGQQPSREENVIMYLFFVLFIVFCSFFTLNLFIGVIIENFNSQKKKAG--GSLEMFMTEDQKKYYNAMKRMQSKSPQKSIPRPKY

L.stagnalis 1399 IDAREIGIQPKREENIYSYLFFVLFIIFGSFFTLNLFIGVIIDNFNSQKKKAG--GSLEMFMTDDQKKYYNAMKRMKSKSPQKSIPRPKY

*D.melanogaster* 1485 IDSREVDKQPIRETNIYMYLYFVFFIIFGSFFTLNLFIGVIIDNFNEQKKKAG--GSLEMFMTEDQKKYYNAMKKMGSKKPLKAIPRPRW

*H.sapiens* Nav1.1 1442 VDSRNVELQPKYEESLYMYLYFVIFIIFGSFFTLNLFIGVIIDNFNQQKKKFG--GQ-DIFMTEEQKKYYNAMKKLGSKKPQKPIPRPGN

*N.vectensis* 1073 VDATKIDEQPIEENNVSAYLFFVVFIILGTFFTLNLFIGVIIDNFNQLKQQMEAVGSMDVFLTSTQRNWMNALKSAATKKPKKRIRRPQN

*T.diomedea* 1488 KLAALIFDITTDQKFDMVIMVIIILNMLTMMFEHYGMSKQMKDILGIFNLIFITVFTTECVLKIIGLRWHYFKIPWNVFDFIVVVLSILA

*M.leonina* 1491 KLAALIFDITTDQKFDVVIMVIIILNMLTMMFEHYGMSEQMKDILAIFNLVFITVFTTECVLKIIGLRWYYFKIPWNVFDFIVVVLSILA

*A.californica* 1488 KLAGLIFDITTDQKFDIAIMVIIILNMLTMMFEHHGMSAQMKNILGIFNLIFITIFTAECVLKLIGLRWYYFKIPWNVFDFVVVVLSILA

L.stagnalis 1487 KLAALVFDITTDQKFDIVIMIIIILNMLTMMFEYEDMSKQMKDILGIFNLVFITIFTAECVLKLFGLRWYYFKVPWNVFDFIVVVLSIMA

*D.melanogaster* 1573 RPQAIVFEIVTDKKFDIIIMLFIGLNMFTMTLDRYDASDTYNAVLDYLNAIFVVIFSSECLLKIFALRYHYFIEPWNLFDVVVVILSILG

*H.sapiens* Nav1.1 1529 KFQGMVFDFVTRQVFDISIMILICLNMVTMMVETDDQSEYVTTILSRINLVFIVLFTGECVLKLISLRHYYFTIGWNIFDFVVVILSIVG

*N.vectensis* 1163 KFQGELYDMVQSRKFEVFIMLFITINMVVMMVQHYDHLAVWSK--QILNLIFTSVFILEAILRIIALRKGYFLNPWNVFDFVIVISSIIG

*T.diomedea* 1578 SSLSEFENSFIISPTLLRVIRVFRVGRVLRLVKSAKGIRTLLFSLAVSLPALFNIGLLLALVMFIYAVMGMNFFQNYPQKYGMDDAFNFD

*M.leonina* 1581 SSLSEIENSFFISPTLLRVIRVFRVGRVLRLVKSAKGIRTLLFSLAVSLPALFNIGLLLALVMFIYAIMGMNFFQNYPQEYGMDDAFNFD

*A.californica* 1578 SSLSEFEDSFFISPTLLRVIRVFRVGRVLRLVKSAKGIRTLLFSMAVSLPALFNIGLLLGLIMFIYAIMGMNFFMGAEQKYGLDDAFNFD

L.stagnalis 1577 SSLDEFEDSFFISPTLLRVIRVFRVGRVLRLVKSAKGIRTLLFSLAVSLPALFNIGLLLGLVMFIYAIMGMNFFQGYPQTFGMDDAFNFD

*D.melanogaster* 1663 LVLSDIIEKYFVSPTLLRVVRVAKVGRVLRLVKGAKGIRTLLFALAMSLPALFNICLLLFLVMFIFAIFGMSFFMHVKEKSGINDVYNFK

*H.sapiens* Nav1.1 1619 MFLAELIEKYFVSPTLFRVIRLARIGRILRLIKGAKGIRTLLFALMMSLPALFNIGLLLFLVMFIYAIFGMSNFAYVKREVGIDDMFNFE

*N.vectensis* 1251 IIVENLQTSLVINPSLLRVVRVFRVGRLLRFFEAARGIRRLLFSLVISTPALFNIGALLFLIIFIYAIIGMSIFGHVKKTEALNDVVNFE

*T.diomedea* 1668 TFLSSLILLFQMCTSAGWSDVLNALISR----CKA---------DEPCTDYSKASLFLASYLIVSFLVVVNMYIAVILENFSKATEEVQQ

*M.leonina* 1671 TFVRSLILLFQMCTSAGWSDVLNGLVSR----CNP---------HDSCTDYTKASVFLASYLIATFLVVVNMYIAVILENFSKATEEVQQ

*A.californica* 1668 TFLRSFILLFQMCTSAGWSDVLNGLIAR----CAP---------EGTCKDYNVATIYLATYLVVSFLVVVNMYIAVILENFSQATEDEQQ

L.stagnalis 1667 TFLSSFILLFQMCTSAGWSDVLNALISP----CPP---------TGSCSHYNKATLYLATYLIISFLVVVNMYIAVILENFSQATEDVQQ

*D.melanogaster* 1753 TFGQSMILLFQMSTSAGWDGVLDAIINE--EACDPPDNDKGY--PGNCGSATVGITFLLSYLVISFLIVINMYIAVILENYSQATEDVQE

*H.sapiens* Nav1.1 1709 TFGNSMICLFQITTSAGWDGLLAPILNSKPPDCDPNKVNPGSSVKGDCGNPSVGIFFFVSYIIISFLVVVNMYIAVILENFSVATEESAE

*N.vectensis* 1341 TFGSSFVLLFRLMTSAGWNDILDPLMIS-EPDCDP--NYRGLP-SGNCGNKYMAPIYLGSFVVIIFLILINMYIAVILENYNQVMEQEKI

*T.diomedea* 1745 GLSPDDFDMYYEKWEKFDPNATKYIPLDQLSDFVDYLEEPLRLPKPNHFILVKLDIPICENDRCYCRDILDALTKNFLG--------TSE

*M.leonina* 1748 GLSPDDFDMYYEKWEKFDPNATKYIPLDQLSDFVDYLEEPLRLPKPNHFILVKLDIPICEGDRCYCRDILDALTKNFLG--------TSE

*A.californica* 1745 GLTPDDFDMYYEKWEKYDPKASKYIPLDQLSDFVDYLEEPLRLPKPNHFILVKLDIPICENDRCYCRDILDALTKNFLG--------TGE

L.stagnalis 1744 GLTPDDFDMYYEKWEKYDPKATKYIPLDQLSDFVDYLEEPLRLPKPNHFILVKLDIPICEGDKCYCRDILDALTKNFLG--------TSE

*D.melanogaster* 1839 GLTDDDYDMYYEIWQQFDPEGTQYIRYDQLSEFLDVLEPPLQIHKPNKYKIISMDIPICRGDLMYCVDILDALTKDFFARKGNPIEETGE

*H.sapiens* Nav1.1 1799 PLSEDDFEMFYEVWEKFDPDATQFMEFEKLSQFAAALEPPLNLPQPNKLQLIAMDLPMVSGDRIHCLDILFAFTKRVLG-------ESGE

*N.vectensis* 1427 GITNEDIELFYQMWELFDPNATQYIPYADLSDFVHQMEGNLRIPKPNKAACALLNIPLVKGDKIHCLDLLQALVKRIVS--GFEDVDSEG

*T.diomedea* 1827 VGDIPQGERE-----EKEKEVYTPISSTLRRQKEHYAARIIQKAYRNYK-----GIVFE----EGDETIDEKQSSNDD-NENDDSDDAS-

*M.leonina* 1830 TGDIPAGESD-----DKEKEEYNPISSTLRRQKEHYAARIIQKAYRNYK-----GTTFD----ERDASNDDKLDLNDDKNDKDDKDEKS-

*A.californica* 1827 TSDIPQKETD------KEKEEYKPISSTLRRQKEHYAARIIQKAYRNFK-----GITFGDGTGSSGKDEDTRSSKSDDDNDDDNGGDGR-

L.stagnalis 1826 TADIPIKETD------KEKEEYTPISSTLRRQKEHYAARIIQKAYRNYK-----GLTISEVSYGHEDVMDSYSQDNDDDRDSGGSSGRNL

*D.melanogaster* 1929 IGEIAAR---------PDTEGYEPVSSTLWRQREEYCARLIQHAWRKHKARGEGGGSFEP---DTDHGDGGDPDAGDPAPDEATDGDAP-

*H.sapiens* Nav1.1 1882 MDALRIQMEERFMASNPSKVSYQPITTTLKRKQEEVSAVIIQRAYRRHL------------------LKRTVKQASFTYNKNKIKGGAN-

*N.vectensis* 1515 FKIVMQRMEERFQAAFPSRTGHKSTITTMEIKRHQEAAKVIVRAIRGY------------------------------------------

*T.diomedea* 1901 ---------------------SKENTDSKNSSDNTEN-DNSNKDSNKQNGCELGKHK--PSSMPES--------KECTVEIVSE---ADA

*M.leonina* 1905 ---------------------LKGDEDDKNSSDDSNSHDGNNKSSNKQNGCDIDKKQ--SSSPSE---------KECDVEIVNE---VDV

*A.californica* 1905 -----------GGGMNGTRGGRRHGAENGEKTKDRDGSKRDGKDSPSKDSNDDSSQQ--AQDKPKETEDEEQRLQDSGIVIVNE---TDA

L.stagnalis 1905 DKSFPSPPSSYKSDKKPPENGTKEKKSEDSSKKAKDKKDKGKDKKAKSDKKDDGKKKKDASKPPNGLSKTTRSKESAAITLINE---TEG

*D.melanogaster* 2006 -----------AGGD-----GSVNGTAEGAADADESNVNSPGEDAAAAAAAAAAAAA--AGTTTAGSPGAGSAGRQTAVLVESDGFVTKN

*H.sapiens* Nav1.1 1953 ---------------------LLIKEDMIIDRINENSITEKTDLTMSTAACPPSYDR--VTKP----------------IVEKH---EQE

*N.vectensis* ------------------------------------------------------------------------------------------

*T.diomedea* 1956 DPKPVELGPESGVVA-----

*M.leonina* 1960 DSKSVELGPESGVVA-----

*A.californica* 1979 DPKTVELGPDSGVVA-----

L.stagnalis 1992 ENKTVELGPNSGIVA-----

*D.melanogaster* 2078 GHKVVIHSRSPSITSRTADV

*H.sapiens* Nav1.1 2001 GKDEKAKGK-----------

*N.vectensis* --------------------

**Figure S4. MUSCLE protein alignment of voltage-gated Na_v_ channel homologues from *Tritonia diomedea*, *Melibe leonina*, *Aplysia californica*, *Lymnaea stagnalis*, *Drosophila melanogaster*, *Homo sapiens* (Na_v_1.1 isotype) and *Nematostella vectensis*.**
