# Supplementary material for: Deep mRNA Sequencing of the Tritonia diomedea Brain Transcriptome Provides Access to Gene Homologues for Neuronal Excitability, Synaptic Transmission and Peptidergic Signalling
Source: PLoS One. 2015 Feb 26;10(2):e0118321. doi: 10.1371/journal.pone.0118321 (PMC4342343; doi:10.1371/journal.pone.0118321)
Supplement: S5 Fig — (DOCX) [file pone.0118321.s006.docx]

*T.diomedea* 1 --------------MASVAAWLPFARASAIGWVPISQNPLPLPTVRPECR------LGDEKLRINVSGRRFETWRTTLEKYPDSLLGSNERDFFYDEDTN

*M.leonina* 1 --------------MASVAAWLPFARASAIGWVPISQNPLPTPTVRPECR------LGDEKLRINVSGRRFETWRTTLEKFPDSLLGSNERDFFYDEESK

*A.californica* 1 --------------MASVAAWLPFARASAIGWVPISQNPLPTPSVRPECR------RGDEKLKINVSGRRFETWRTTLEKFPDSLLGSNEREFFYDEELK

*L.stagnalis* 1 --------------MASVAAWLPFARASAIGWVPISEHPLPNPTVRPETR------RGDEKLKINVSGRRFETWRTTLEKFPDSLLGSNEREFFYDEESK

*D.melanogaster* 1 --------------MASVAAWLPFARAAAIGWVPIATHPLPPPPMPKDRR-----KTDDEKLLINVSGRRFETWRNTLEKYPDTLLGSNEREFFYDEDCK

*C.elegans* 1 --------------MASVAAWLPFARAAAIGWVPISRQPMPQAPVAIQAKDLAVDHVSDEKLAINISGRRFETWKNTLEKFPETLLGSNEKEFFYDEDTG

*H.sapiens Kv4.2* 1 -------------MAAGVAAWLPFARAAAIGWMPVASGPMPAPPRQERKR------TQDALIVLNVSGTRFQTWQDTLERYPDTLLGSSERDFFYHPETQ

*N.vectensis* 1 MYPSHTSNNLCSAMAADVVAWLPFARAAAIGWVPLATSPMPPP---PESS------KTDERVTINVSGRRFETWRNTLARFPETLLGSDEKDYFYDAETK

*T.diomedea* 81 EYFFDRDPDIFRHILNYYRTSRLHYPKHECISSYDEELAFFGILPDIIGDCCYEDYRDRKRENAERLID----DEKSENGD--FDPAISLREQMWRAFEN

*M.leonina* 81 EYFFDRDPDIFRHILNYYRTSRLHYPKHECISSYDEELAFFGILPDIIGDCCYEDYRDRKRENAERLID----DEKSENGD--FDPAISLREQMWRAFEN

*A.californica* 81 EYFFDRDPDIFRHILNYYRTSRLHYPKHECISAYDEELAFFGILPDIIGDCCYEDYRDRKRENSERLID----DEKSDNGD--FDPAVSLREKMWRAFEN

*L.stagnalis* 81 EYFFDRDPDIFRHILNYYRTSRLHYPKHECISSYDEELAFFGILPDIIGDCCYEDYRDRKRENSERLID----DEKSENGD--YDPAISLREKMWRGVEN

*D.melanogaster* 82 EYFFDRDPDIFRHILNYYRTGKLHYPKHECLTSYDEELAFFGIMPDVIGDCCYEDYRDRKRENAERLMD----DKLSENGDQNLQQLTNMRQKMWRAFEN

*C.elegans* 87 EYFFDRDPDIFRHILTFYRTGKLHYPRHECLVAYDEELSFFGIMPDLISDCCYEDYKDKKRENQERLQE-----ERVENAD-ISTLKLSLKEKMWAAFEN

*H.sapiens Kv4.2* 82 QYFFDRDPDIFRHILNFYRTGKLHYPRHECISAYDEELAFFGLIPEIIGDCCYEEYKDRRRENAERLQD----DADTDTAGESALPTMTARQRVWRAFEN

*N.vectensis* 92 EYFFDRDPDLFRHLLNYYRNGKLHYPRGECVSSFEDELEFFGISEDVVHDCCWEDFRERKKECMERIFEPDKLDDASQNGDEVTDH--SIREKLWTAFQN

*T.diomedea* 175 PHIGTVALVFYYVTGFFIAVSVMANVVETIKCGNVPGARVEPTCGERYNIAFFCLDTACVMIFTAEYLVRLYAAPDRFKFMRSVMSIIDVVAIIPYYIGL

*M.leonina* 175 PQIGTVALVFYYVTGFFIAVSVIANVVETIRCGPVPGARIEPTCGERYEIAFFCLDTACVMIFTAEYLLRLYAAPDRIRFMRSVMSIIDVVAIIPYYIGL

*A.californica* 175 PHIGTVALVFYYVTGFFIAVSVIANVVETVPCGKIPGALEELSCGERHQPAFFCLDTACVMIFTAEYLLRLYAAPDRCKFVRSVMSIIDVVAIIPYYIGL

*L.stagnalis* 175 PHIGTVALVFYYVTGFFIAVSVIANVIETVPCGKVPGARKELSCGEKFEIAFFCLDTACVMIFTAEYLLRLYAAPDRCKFMRSVMSIIDVVAIIPYYIGL

*D.melanogaster* 178 PHTSTSALVFYYVTGFFIAVSVMANVVETVPCGHRPGRAGTLPCGERYKIVFFCLDTACVMIFTAEYLLRLFAAPDRCKFVRSVMSIIDVVAIMPYYIGL

*C.elegans* 181 PHTTSIALVFYYVIGFFIAVSVMCNIVETIPCGY--EDNVSVTCGEAYEEQFFVIDTACVIIFTIEYFLRLISAPDRIKFMRSIMSVIDVIAIMPYYVSL

*H.sapiens Kv4.2 178 PHTSTMALVFYYVTGFFIAVSVIANVVETVPCGSSPGHIKELPCGERYAVAFFCLDTACVMIFTVEYLLRLAAAPSRYRFVRSVMSIIDVVAILPYYIGL*

*N.vectensis* 190 PQSSKVASVFYYITGLFIAISVISTVVETLPC------KGKTSCGEVHKTVFFSLEAACVVVFTVEYVARLYSAPDRVKFARDLLSIIDVVAILPFYVGL

*T.diomedea* 275 VISDNDDVSGAFVTLRVFRVFRIFKFSRHSQGLRILGYTLKSCASELSFLLFSLTMAIIIFATIMYYAEKNVENTTFTSIPSAFWYTIVTMTTLGYGDMT

*M.leonina* 275 VISDNDDVSGAFVTLRVFRVFRIFKFSRHSQGLRILGYTLKSCASELGFLLFSLSMAIIIFATIMYYAEKNEPNTTFTSIPPAFWYTIVTMTTLGYGDMT

*A.californica* 275 GISDNDDVSGAFVTLRVFRVFRIFKFSRHSQGLRILGYTLKSCASELGFLLFSLTMAIIIFATIMFYAEKNMTGTTFTSIPAAFWYTIVTMTTLGYGDMT

*L.stagnalis* 275 VISDNDDVSGAFVTLRVFRVFRIFKFSRHSQGLRILGYTLKSCASELGFLLFSLTMAIIIFATIMFYAERSVEGTTFTSIPAAFWYTIVTMTTLGYGDMT

*D.melanogaster* 278 GITDNDDVSGAFVTLRVFRVFRIFKFSRHSQGLRILGYTLKSCASELGFLVFSLAMAIIIFATVMFYAEKNVNGTNFTSIPAAFWYTIVTMTTLGYGDMV

*C.elegans* 279 VLTDNKDVSGLFVTLRVFRVFRIFKFSRHSQGLRILGYTLKSCASELGFLVFSLAMAIIIFATIMYYAEKKVDATRFTSIPSAFWYTIVTLTTLGYGDMV

*H.sapiens Kv4.2* 278 VMTDNEDVSGAFVTLRVFRVFRIFKFSRHSQGLRILGYTLKSCASELGFLLFSLTMAIIIFATVMFYAEKGSSASKFTSIPAAFWYTIVTMTTLGYGDMV

*N.vectensis* 284 IVPNN-SISGAFVTLRVFRIFRIFKFSRHSRGLRILGYTLKSCASELGFLLFSLSMAVIIFATVMYYVEKGEVDTKFISIPASFWYTIVTMTTLGYGDMV

*T.diomedea* 375 PDTITGKIVGGVCSLSGVLVIALPVPVIVSNFSRIYHQNQRADKRKAQKKAREARINMAKNASSAGFISAKKRAEEIILARETG-IELEASRHKGDIFEL

*M.leonina* 375 PDTISGKIVGGICSLSGVLVIALPVPVIVSNFSRIYHQNQRADKRKAQKKAREARVNMAKNASSAGFIHAKKRAEEVLLAQETG-MELEQN-HKGDIFEL

*A.californica* 375 PDTITGKIVGGVCSLSGVLVIALPVPVIVSNFSRIYHQNQRADKRKAQKKAREARINMAKNASSAGFISAKKRAEEMMMARDAG-MDVDEN-HKGDIFEM

*L.stagnalis* 375 PDTITGKIVGGVCSLSGVLVIALPVPVIVSNFSRIYHQNQRADKRKAQKKARETRIHMAKNASSAGFISAKKRAEEALLARESG-LDMEEY-NKGDIFEM

*D.melanogaster* 378 PETIAGKIVGGVCSLSGVLVIALPVPVIVSNFSRIYHQNQRADKRKAQRKARLARIRIAKASSGAAFVSKKKAAEARWAAQESG-IELDDNYRDEDIFEL

*C.elegans* 379 PSTIMGKIVGGVCSLSGVLVIALPVPVIVSNFSRIYHQNQRADKRKAQKKARLARIRIVKNASGLALFNKKRAHEARMLAFEQGHLSFDAL-RDEDIFEI

*H.sapiens Kv4.2* 378 PKTIAGKIFGSICSLSGVLVIALPVPVIVSNFSRIYHQNQRADKRRAQKKARLARIRAAKSGSANAYMQSKRSGLLSNQLQSSE-DEPAFVSKSGSSFET

*N.vectensis* 383 PTTVPGKIVGSLCSLSGVLVIALPVPVIVSNFSRIYLQNQRADKRKAHKKA---RVSISKTMAGTALVSPPDKKNVPLGAGLEL-TQIPSKDNESSYLEE

*T.diomedea* 474 QHHHLLTCLEKTTDREFVEMDMTFNGSPNKPSETP---PPSPDPSVISE-RN---TGCCAKRFSPHRSLMAKK---E--RSNQGIEREELNDVRLRPAPL

*M.leonina* 473 QHHHLLSCLEKTTDREFVEMDMTFNGSPNKPSETP---PPSPDPFLAPD-NT---TGCCAKRFFPPRSLMAKKDSEE--RSIQGIEREEVNDVRLRPAPL

*A.californica* 473 QHHHLLTCLEKTTDREFVEMDMTFNGAPNNPSETP---PPSPDPSLGSNRRK---SSCCARSFSPNRKLMSNKPK-RSLPTHHSIERQELNDVRMRTSP-

*L.stagnalis* 473 QHHHLLSCLEKTTDREFVEMDMTFNNAPNKPSETP---PPSPDPSIVSKGRK---SRCCGKKFSPKSKLMQEKKRSQSYHSKHSIEHQEMNDVRMRLSP-

*D.melanogaster* 477 QHHHLLRCLEKTTDREFVELEIPFNGQPKRPG-SP---SPMASPAHSTNSAAGLLQSCCGRCCSQRYQAC------------------------------

*C.elegans* 478 QHHHLLQCLEKATEREFVESEVMFEGGRN----TP---PPSETASLKGKTKR-------------KRRLCCVSKENE--------EMEELD---------

*H.sapiens Kv4.2* 477 QHHHLLHCLEKTTNHEFVDEQVFEESCMEVATVNR---PSSHSPSLSSQQGV--TSTCCSRRHKKTFRIPNANVSGS-----HRGSVQELSTIQIRCV--

*N.vectensis* 479 QHHHLLHCLEKATARQFVEMEYTYNGEPVAARRSPKLSSPICTPSGSPISSSVSLAAVCDNACYNRNSYQGKCMKRT--RSFSEPTHKHENSVLI-----

*T.diomedea* 562 SKREASMSSALN---LQGFSNTSYNKDGGGGNGIGGGGGRRDGGGRGD-EGGG-----------DKDLH--GKSNLGNNKANSTQNI-QRNYQPQMT-GS

*M.leonina* 564 GRKQESRSSNLN---LQGFSNTSYNKEGSGGGGVRGGGGGGGGGGRGDLDGGGGIGHDDGGDEEDGGVHHFGSYNLSNNKANSTQNIHHRNFQSAITGGS

*A.californica* 565 GSHHDSRSSDVNILDVQGFSNAGHTRSSSCG-------------------GSG------------------GNNGLGNNKANSTQNIHNLNSQPNIAALD

*L.stagnalis* 566 ASKTDSRSSGVNVLEIQGFSNTNHNRG--------------------------------------------GNNGLGNNKSNSSQDI-YANLHPNFTSGL

*D.melanogaster* 543 -----------------------------------------------------------------------GKYMPAASNAQNSQNNQ------------

*C.elegans* 541 ----------------------------------------------------------------------------RETRVTFNQNMDQICELPK-----

*H.sapiens Kv4.2* 565 -----------------------------------------------------------------------ERTPLSNSRSSLNAKMEECV---------

*N.vectensis* 572 -----------------------------------------------------------------------GDHNVNDFKTCNNTHGHSNNKRPA-----

*T.diomedea* 643 QQNMSIVSAG------MHTAGSQPNVVVPW---NSSGSQQNITTAVITN-PNSTGSTHSVPD----MTMPAAQVSSSPSSTSSTT-KGEVVRISTL

*M.leonina* 661 QQNLSITSGGSVGGGLQQSGGSQPNVVVPWSQASSPDSQQNVTTALISN-PNSTGSTHSVPDDT--TTPQTAQVSSSPSSTSSNAGKGDVVRISTL

*A.californica* 628 TS--RLINSGGIASASGGQHSSQPNIGS--LGKPLSSSQQNITTALVSSNPMSAGSTHSVPEAMPTTAPNTAHGNSTPSSSSSN--KGDVVRISTL

*L.stagnalis* 621 RNA-------------CVTSGSEPNMGSKL---TSSTSHHNVTTALVSS-PNSTGSSQSVPD------AVAAHSSFLPSSTN----KGDIVRISTL

*D.melanogaster* 560 --------------------------------------------------PM----------------------------------DGTYLVEASF

*C.elegans* 560 --------------------------------------------------PDEMELNHR---------------------------NNDKICVSQL

*H.sapiens Kv4.2* 585 ---------------------------------KLNCEQPYVTTAII---SIPTP-----PV----TTPEGDDRPESPEYS-----GGNIVRVSAL

*N.vectensis* 596 --------------------------------------NNNVTTSAVVTMPDF--ETVAVPN----NIHEPGRFPAVPPPSYENCNDRRSSSHTAI

**Figure S5. MUSCLE protein alignment of voltage-gated A-type K_v_ channel homologues from *Tritonia diomedea*, *Melibe leonina*, *Aplysia californica*, *Lymnaea stagnalis*, *Drosophila melanogaster*, *Caenorhabditis elegans*, *Homo sapiens* (K_v_4.2 isotype) and *Nematostella vectensis*.**
