# Supplementary material for: Deep mRNA Sequencing of the Tritonia diomedea Brain Transcriptome Provides Access to Gene Homologues for Neuronal Excitability, Synaptic Transmission and Peptidergic Signalling
Source: PLoS One. 2015 Feb 26;10(2):e0118321. doi: 10.1371/journal.pone.0118321 (PMC4342343; doi:10.1371/journal.pone.0118321)
Supplement: S6 Fig — (DOCX) [file pone.0118321.s007.docx]

*T.diomedea* 1 ------------------------------MESISRLRRA--------------------AANAVETTAKSVNEAVS--NGNTTSPTEGG
*M.leonina* 1 ------------------------------MESIVRLRRA--------------------AATVAETTAKSVSDAVSSANENTTASSEDG
*A.californica* 1 -----------------------------MDSLLARVKRA----------------ADADALNPAQEGVTGGPDAAGLPDVSTSSPGGGG
*L.stagnalis* 1 ------------------------MPALGAMEALVRLRRL--------------------AMDASVTGAPSSADETSTAGEP--------
*D.melanogaster* 1 MPPNAKSETDAKPEAEPAPASEPAADLESVDQKLEETHHSKFREVDRQEQEVLAEKAAEAASQRIAQVESTTRSATTEAQESTTTAVPVI
*C.elegans* 1 --------------------MVKLDFSSQDEENDEDLTKE--------------------FVRDEAPMEETTSEAVKQIATTTKETLKDV
*H.sapiens* 1 --------------------------------MVSESHHE------------------------ALAAPPVTTVATVLPSNATEPASPGE
*N.vectensis* 1 ----------------------------MGNPLLDKTHGG--------------VGGGQDLLNRKQEMPVSQNDPVAKIQGPTPTPSATP


*T.diomedea* 39 ---GLKDRVGEVKDKIINEFLKLPI--WAIILIIAGSLLFLVCCVYCMCKRCC-RKRKKKEGK-KGLKGAVDLKSVQLLGNSYKEKVQPD
*M.leonina* 41 ---GLKDRVGEVKDKIMKEFLKLPI--WAIILIIAGSLLFLVCCVYCTCKRCC-RKRKKKEGK-KGLKGAVDLKSVQLLGNSYKEK--PD
*A.californica* 46 AGDKLKEKMDEYKDKLINEIENLPI--WAIVLIIAGSLLFLVCCVYCVCRRCC-RKRKKKEGK-KGLKGAVDLKSVQLLGNSYKEKVQPD
*L.stagnalis* 39 ---EQKGKLDEIKDSIMNEINKLPI--WAIILIIAGALLFLSCCVYCVCRRCC-RKRKKKEGK-KGLKGAVDLKSVQLLGNSYKEKVQPD
*D.melanogaster* 91 ---KKIEHVGEVVTEVIAERTGLPT--WGVVAIIILVFLVVFGIIFFCVRRFL-KKRRTKDGK---GKKGVDMKSVQLLGSAYKEKVQPD
*C.elegans* 51 VVNKVIDVKDVVKEKVMQQ-TGMPE--WAFVFLGFVFILLVLACAFCLIRKLFGKKRHGEKNK-KGGLKGFFGKGQDVVDGKNIQGMAQD
*H.sapiens* 35 ---GKEDAFSKLKEKFMNELHKIPLPPWALIAIAIVAVLLVLTCCFCICKKCLFKKKNKKKGKEKGGKNAINMKDVKDLGKTMKDQALKD
*N.vectensis* 49 ----------VTNPSILGQ---IPI--WIIAVVGIGAAVIVIFCIYCCCCRKC-GKKKKKDDK-RGGKERVDFRAVQ-IGASYQEKVQPS


*T.diomedea* 122 LDELPVNMEDNE-DAESTKSEVKLGKLQFSLDYDFQKGELMVNVIQAADLPGMDMSGTSDPYVKVYLLPDKKKKYETKVHRKTLNPVFNE
*M.leonina* 122 LDELPVNMEDNE-DAESTKSEVKLGKLQFSLDYDFQKGELSVNVIQAADLPGMDMSGTSDPYVKVYLLPDKKKKHETKVHRKTLNPVFNE
*A.californica* 132 LDELPVNMEDNE-DAESTKSEVKLGKLQFSLDYDFQKGELSVNVIQAADLPGMDMSGTSDPYVKVYLLPDKKKKYETKVHRKTLNPVFNE
*L.stagnalis* 122 LDELPVNMEDNE-DAESTKSEVKLGKLQFSLDYDFQKGELSVNVIQAADLPGMDMSGTSDPYVKVYLLPDKKKKYETKVHRKTLNPVFNE
*D.melanogaster* 172 MEELTENAEEG--DEEDKQSEQKLGRLNFKLEYDFNSNSLAVTVIQAEELPALDMGGTSDPYVKVYLLPDKKKKFETKVHRKTLSPVFNE
*C.elegans* 137 LEELGDAMEQNEKEQAEEKEEVKLGRIQYKLDYDFQQGQLTVTVIQAEDLPGMDMSGTSDPYVKLYLLPEKKKKVETKVHRKTLNPVFNE
*H.sapiens* 122 -DDAETGLTDGE-EKEEPKEEEKLGKLQYSLDYDFQNNQLLVGIIQAAELPALDMGGTSDPYVKVFLLPDKKKKFETKVHRKTLNPVFNE
*N.vectensis* 121 MDELDYNSEDYH---SDLSSGVKIGRINFTLDYSFTDNTLTVGIIRAEDIPAKDFSGSSDPYVKIMLLPDKKKKYETKVHRKTLNPVFNE


*T.diomedea* 211 TFTFK-VPYAEVGSKILTFAVFDFDRFSKHDQIGQVQVPLNSIDLGRVVEEWKDLTAPDSESE---KENKLGDICFSLRYVPTAGKLTVV
*M.leonina* 211 SFTFK-VPYAEVGSKILTFAVFDFDRFSKHDQIGQVQVPLNSIDLGRVVEEWKDLVPPDSESE---KENKLGDICFSLRYVPTAGKLTVV
*A.californica* 221 SFTFK-VPYAEVGSKILTFAVYDFDRFSKHDQIGQVQVPLNSIDLGRVVEDWKDLQSPDTESE---KENKLGDICFSLRYVPTAGKLTVV
*L.stagnalis* 211 SFTFK-VPYAEVGSKILTFAVYDFDRFSKHDQIGQVQVPLNSIDLGRVVEEWRDLQSPDTESE---KENKLGDICFSLRYVPTAGKLTVV
*D.melanogaster* 260 TFTFKSLPYADAMNKTLVFAIFDFDRFSKHDQIGEVKVPLCTIDLAQTIEEWRDLVSVEGEGG---QE-KLGDICFSLRYVPTAGKLTVV
*C.elegans* 227 TFIFK-VAFNEITAKTLVFAIYDFDRFSKHDQIGQVLIPLGKIDLGAVIEEWKDIAPPPDDKE---AEKSLGDICFSLRYVPTAGKLTVV
*H.sapiens* 210 QFTFK-VPYSELGGKTLVMAVYDFDRFSKHDIIGEFKVPMNTVDFGHVTEEWRDLQS--AEKE---EQEKLGDICFSLRYVPTAGKLTVV
*N.vectensis 208 QFVFKNIPYSEITNRILLMELFDFDRFSRHDLIGEARLPLIDVDLASNINEWRVLTPPSGSGGAGHSKSDLGDICFSLRYVPSSGKLQIT*

*T.diomedea* 297 ILEAKNLKKMDVGGLSDPYVKIALLQGSKRLKKKKTTIKKNTLNPYFNESFGFEVPFEQIQKVTLIITVVDYDRIGTSEPIGRCLLGCNS
*M.leonina 297 ILEAKNLKKMDVGGLSDPYVKIALLQGSKRLKKKKTTIKKNTLNPYFNESFGFEVPFEQIQKVTLIITVVDYDRIGTSEPIGRCLLGCNS
A.californica* 307 ILEAKNLKKMDVGGLSDPYVKISLMLNGKRVKKKKTTIKKCTLNPYYNESFTFEVPFEQIQKVTLIITVVDYDRIGTSEPIGRCVLGCNS
*L.stagnalis* 297 ILEAKNLKKMDVGGLSDPYVKISLMLNGKRVKKKKTTIKKCTLNPYYNESFTFEVPFEQIQKVTLIVTVVDYDRIGTSEPIGRCVLGCNS
*D.melanogaster* 346 ILEAKNLKKMDVGGLSDPYVKIAIMQNGKRLKKKKTSIKKCTLNPYYNESFSFEVPFEQIQKICLVVTVVDYDRIGTSEPIGRCILGCMG
*C.elegans* 313 ILEAKNLKKMDVGGLSDPYVKIVLMQGGKRLKKKKTSIKKCTLNPYYNESFSFEVPFEQIQKVSLMITVMDYDKLGSNDAIGRCLLGCNG
*H.sapiens* 294 ILEAKNLKKMDVGGLSDPYVKIHLMQNGKRLKKKKTTIKKNTLNPYYNESFSFEVPFEQIQKVQVVVTVLDYDKIGKNDAIGKVFVGYNS
*N.vectensis* 298 IVEAKSLKSMDLTGYSDPYVKIALVQEGRKIKKKKTTVKKRTLNPYYNETFTFTVAFEKIEQTSLIISVLDYDRVGKSEMIGKCVVGELS


*T.diomedea* 387 SGTELRHWSDMLANPRRPIAQWHTLQEVPEKN-------
*M.leonina* 387 SGTELRHWSDMLANPRRPIAQWHTLQEVPEKN-------
*A.californica* 397 SGTELRHWSDMLANPRRPIAQWHTLQEVPEKN-------
*L.stagnalis* 387 SGTELRHWSDMLANPRRPIAQWHTLQEVPEKS-------
*D.melanogaster* 436 TGTELRHWSDMLASPRRPIAQWHTLKDPEETDEILKNMK
*C.elegans* 403 TGAELRHWMDMLASPRRPIAQWHTLGPVEEEGDKKDDKK
*H.sapiens* 384 TGAELRHWSDMLANPRRPIAQWHTLQVEEEVDAMLAVKK
*N.vectensis* 388 SGADLRHWADMLASPRRSVAQWHTLHN------------


**Figure S6. MUSCLE protein alignment of synaptotagmin homologues from *Tritonia diomedea*, *Melibe leonina*, *Aplysia californica*, *Lymnaea stagnalis*, *Drosophila melanogaster*, *Caenorhabditis elegans*, *Homo sapiens* and *Nematostella vectensis*.**
