# Supplementary material for: Deep mRNA Sequencing of the Tritonia diomedea Brain Transcriptome Provides Access to Gene Homologues for Neuronal Excitability, Synaptic Transmission and Peptidergic Signalling
Source: PLoS One. 2015 Feb 26;10(2):e0118321. doi: 10.1371/journal.pone.0118321 (PMC4342343; doi:10.1371/journal.pone.0118321)
Supplement: S7 Fig — (DOCX) [file pone.0118321.s008.docx]

*T.diomedea* 1 -----------MAAPQNPQAGPGGPPSAGVGGPQ--QGMQPPRE--QSKRLQQTQAQVDEVVDIMRVNVEKVLDRDQKISQLDDRAEALQ
*M.leonina* 1 -----------MAAPQNPQAGPGGPPSAGVGGPQGAGGMQPPRE--QSKRLQQTQAQVDEVVDIMRVNVEKVLDRDQKISQLDDRAEALQ
*A.californica* 1 -------------------------MSAGPGGPQ--GGMQPPRE--QSKRLQQTQAQVDEVVDIMRVNVEKVLDRDQKISQLDDRAEALQ
*L.stagnalis* 1 -----------MAASQNPQAGPGGPPSAGPGGP----GMQPPRE--QSKRLQQTQAQVDEVVDIMRVNVEKVLDRDQKISQLDDRAEALQ
*D.melanogaster* 1 MGKKDKNKEQADAAPAGDAPPNAGAPAGEGGDGEIVGGPHNPQQIAAQKRLQQTQAQVDEVVDIMRTNVEKVLERDSKLSELDDRADALQ
*C.elegans* 1 ----------------MDAQGDAGAQGGSQGGPR------P-----SNKRLQQTQAQVDEVVGIMKVNVEKVLERDQKLSQLDDRADALQ
*H.sapiens* 1 -----------MSAPAQPPAEGTEGTAPGGGPPG------PPPNMTSNRRLQQTQAQVEEVVDIIRVNVDKVLERDQKLSELDDRADALQ
*N.vectensis* 1 ---------------------------------------------MSSKRLQQTQAQVDEVVDIMKVNVDKVLERDAKLSELDNRADALQ


*T.diomedea* 76 AGASQFEASAGKLKRKYWWKNCKMMLILGAIIGIIVIIIIVWVVEGTDSKSSSSGAPKPVGTTSATPANGS-------------------
*M.leonina* 78 AGASQFEASAGKLKRKYWWKNCKMMLILGAIIGIIVIIIIVWAVQGSGGGGDSSSGPAPPAPAPLPTGAPPTTVKGN-------------
*A.californica* 62 AGASQFEASAGKLKRKYWWKNCKMMLILGAIIGVIVIIIIVWVVTSQDSGGDDSGSKTPATAGTSPKPVESGVQGGGGRQQRPHSQLVER
*L.stagnalis* 74 AGASQFEASAGKLKRKYWWKNCKMMLILGAIIGIICIIIIVWVVTSTKGGDDKPT-PQPAISSTTGTPSPKTT-----------------
*D.melanogaster* 91 QGASQFEQQAGKLKRKFWLQNLKMMIIMG-VIGLVVVGIIAKKDEE--------------------------------------------
*C.elegans* 64 EGASQFEKSAATLKRKYWWKNIKMMIIMCAIVVILIIIIVLWAGGK--------------------------------------------
*H.sapiens* 74 AGASQFESSAAKLKRKYWWKNCKMMIMLGAICAIIVVVIVIYFFT---------------------------------------------
*N.vectensis* 46 AGASQFETSASRLKRKMWWQNCKMWIILCIVVIVIIAVIVIWVVTSTGTGSSSSSSSTAAPATIKP------------------------


*T.diomedea* -----------------------------
*M.leonina* -----------------------------
*A.californica* 152 RNVLRRTEDHIGCRPHIHSFIHIFMICLV
*L.stagnalis* -----------------------------
*D.melanogaster* -----------------------------
*C.elegans* -----------------------------
*H.sapiens* -----------------------------
*N.vectensis* -----------------------------


**Figure S7. MUSCLE protein alignment of synaptobrevin homologues from *Tritonia diomedea*, *Melibe leonina*, *Aplysia californica*, *Lymnaea stagnalis*, *Drosophila melanogaster*, *Caenorhabditis elegans*, *Homo sapiens* and *Nematostella vectensis*.**
