# Supplementary material for: Deep mRNA Sequencing of the Tritonia diomedea Brain Transcriptome Provides Access to Gene Homologues for Neuronal Excitability, Synaptic Transmission and Peptidergic Signalling
Source: PLoS One. 2015 Feb 26;10(2):e0118321. doi: 10.1371/journal.pone.0118321 (PMC4342343; doi:10.1371/journal.pone.0118321)
Supplement: S8 Fig — (DOCX) [file pone.0118321.s009.docx]

*T.diomedea* 1 --MNGTEGEEVPP--EELTEDDLLRKQIEDGTNESLESTRRMLSMCEESKEAGIKTLVMLDEQGEQLDRIEEGMDQINQDMRDAEKNLEG
*M.leonina* 1 --MSGTAEEEPPPAPEDDTEEGRLKRQIEDGTNESLESTRRMLSMCEESKEAGIKTLVMLDEQGEQLDRINEGMDQINQDMRDAEKNLEG
*A.californica* 1 ---MAAPAEEGGGETEPLSEDAILRQQIEDGTNESLDSTRRMLTMCEESKEAGIKTLVMLDEQGEQLDRIEEGMDQINQDMRDAEKNLEG
*L.stagnalis* 1 MTTNGEILPVGEEEEEELGEDALLRKQIDCNTNESLESTRRMLSLCEESKEAGIKTLVMLDEQGEQLDRIEEGMDQINQDMRDAEKNLEG
*D.melanogaster* 1 --MPADPSEEVAPQ-VPKTELEELQINAQGVADESLESTRRMLALCEESKEAGIRTLVALDDQGEQLDRIEEGMDQINADMREAEKNLSG
*C.elegans* 1 ----------MSGDDDIPEGLEAINLKMNATTDDSLESTRRMLALCEESKEAGIKTLVMLDDQGEQLERCEGALDTINQDMKEAEDHLKG
*H.sapiens* 1 ----------MAEDADMRNELEEMQRRADQLADESLESTRRMLQLVEESKDAGVRTLVMLDEQGEQLERIEEGMDQINKDMKEAEKNLTD
*N.vectensis* 1 ----------MSEEDRMRNEIARMQMRGDEMTDESLESTRRMLRMAEESQDTGIKTMVMIDEQGEQLDRVEEGLDQINADMKEAERNLTG


*T.diomedea* 87 LEKCCGLCVLPWKRFKNFEKGSDYNKTWKA--SEDGKVNTNGPRVMVDSG---NGAGPSGGFITRITNDAREDEMEQNISEVSGMVGNLR
*M.leonina* 89 LEKCCGLCVLPWKRFKNFEKGSDYDKTWKA--SEDGKVNTNGPRVMVDSG---NGAGPSGGFITRITNDAREDEMEQNISEVSGMVGNLR
*A.californica* 88 LEKCCGLCVLPWKRSKNFEKGSDYNKTWKA--SEDGKVNTNGPRVMVDQG---NGGGPTGGYITRITNDAREDEMEQNMVEVSGMVGNLR
*L.stagnalis* 91 LEKCCGLCVLPWKRSKNFEKGSDYNKTWKA--SEDGKINTNGPRLVVDQG---NGSGPTGGYITRITNDAREDEMEQNIGEVAGMVSNLR
*D.melanogaster* 88 MEKCCGICVLPCNKSQSFKED---DGTWKG--NDDGKVVNNQPQRVMDDR---NGMMAQAGYIGRITNDAREDEMEENMGQVNTMIGNLR
*C.elegans* 81 MEKCCGLCVLPWNKTDDFEK-TEFAKAWKK--DDDGGVISDQPRITVGD----SSMGPQGGYITKITNDAREDEMDENVQQVSTMVGNLR
*H.sapiens* 81 LGKFCGLCVCPCNKLKS---SDAYKKAWGN--NQDGVVASQ-PARVVDER---EQMAISGGFIRRVTNDARENEMDENLEQVSGIIGNLR
*N.vectensis* 81 MEKCCGLCVCPWKKSKRYEKSDTYKKAFKNNHNEDGVVSSQ-P-VRYGQGSSTDGSGPSGGYIQRITNDDREDEMDENLGQVSNIIGNLK


*T.diomedea* 172 NMAVDMGNEIESQNRQLDRINQKGTSNESRITAANQRATKLLKEA-------
*M.leonina* 174 NMAVDMGNEIESQNRQIDRINQKGMSNETRITAANQRATKLLKEALKEA---
*A.californica* 173 NMAVDMGNEIESQNRQLDRINQKGMSNETRITAANQRATKLLKEALLKEA--
*L.stagnalis* 176 NMAVDMGNEIESQNKQLDRINQKGMSNEVRITAANQRATKLLKEATKLLKEA
*D.melanogaster* 170 NMALDMGSELENQNRQIDRINRKGESNEARIAVANQRAHQLLK--KEA----
*C.elegans* 164 NMAIDMSTEVSNQNRQLDRIHDKAQSNEVRVESANKRAKNLITK-LKEA---
*H.sapiens* 162 HMALDMGNEIDTQNRQIDRIMEKADSNKTRIDEANQRATKMLGSG-------
*N.vectensis* 169 SMAVDMGQELETQNRQLDRINAKAESNDTRIHVANKRARDILRNA-------


**Figure S8. MUSCLE protein alignment of SNAP-25 homologues from *Tritonia diomedea*, *Melibe leonina*, *Aplysia californica*, *Lymnaea stagnalis*, *Drosophila melanogaster*, *Caenorhabditis elegans*, *Homo sapiens* and *Nematostella vectensis*.**
