# Supplementary material for: Deep mRNA Sequencing of the Tritonia diomedea Brain Transcriptome Provides Access to Gene Homologues for Neuronal Excitability, Synaptic Transmission and Peptidergic Signalling
Source: PLoS One. 2015 Feb 26;10(2):e0118321. doi: 10.1371/journal.pone.0118321 (PMC4342343; doi:10.1371/journal.pone.0118321)
Supplement: S9 Fig — (DOCX) [file pone.0118321.s010.docx]

*T.diomedea* 1 ---------------------------------------MTKDRLAALKAAQSDDDD-----NDEVAVTVDSNGFNFMEEFFEQVDEIRE
*M.leonina* 1 ---------------------------------------MTKDRLAALKAAQSDDDD-----NDEVAVTVDSNGFNFMEEFFEQVDEIRE
*A.californica* 1 ---------------------------------------MTKDRLAALKAAQSDDDD-----NDDVAVTVDSSG--FMEEFFEQVDEIRE
*L.stagnalis* 1 ---------------------------------------MTKDRLAALKAAQSDDDE-----NDDVAVTVDSSG--FMEEFFEQVDEIRE
*D.melanogaster* 1 ---------------------------------------MTKDRLAALHAAQSDDEE-----ETEVAVNVDGHD-SYMDDFFAQVEEIRG
*C.elegans* 1 ---------------------------------------MTKDRLSALKAAQSEDEQ-----DDDMHMDTGNAQ--YMEEFFEQVEEIRG
*H.sapiens* 1 ---------------------------------------M-KDRTQELRTAK-DSDD-----DDDVAVTVDRDR--FMDEFFEQVEEIRG
*N.vectensis* 1 MPVTLRARESSNGEKGGGACDRVGTEKHTKHRLSRSFTSM-RDRLDFLRAEDNTRDDGPPEYEDSIAIPMGGE---FMDDFFQQTANIRE


*T.diomedea* 47 MIDKIASNVDEVKKKHSAILSAPQTDDKMKEDLEELMSEIKKNANKVRAKLKVIEQNIEQEEHTNKS----SADLRIRKTQHATLSRKFV
*M.leonina* 47 MIDKIASNVDEVKKKHSAILSAPNTDDRMKEELEELMSEIKKNANKVRAKLKVIEQNIEQEEHSNKS----SADLRIRKTQHATLSRKFV
*A.californica* 45 MIDKIASNVDEVKKKHSAILSAPQTDDKMKEELEELMSEIKKNANKVRAKLKVIEQNIEQEEHTNKS----SADLRIRKTQHATLSRKFV
*L.stagnalis* 45 MIDKIASNVDEVKKKHSAILSAPQTDDKMKEELEELMSEIKKNANKVRAKLKVIEQNIEQEEHTNKS----SADLRIRKTQHATLSRKFV
*D.melanogaster* 46 MIDKVQDNVEEVKKKHSAILSAPQTDEKTKQELEDLMADIKKNANRVRGKLKGIEQNIEQEEQQNKS----SADLRIRKTQHSTLSRKFV
*C.elegans* 45 SVDIIANNVEEVKKKHSAILSNPVNDQKTKEELDELMAVIKRAANKVRGKLKLIENAIDHDEQ-GAG----NADLRIRKTQHSTLSRRFV
*H.sapiens* 43 FIDKIAENVEEVKRKHSAILASPNPDEKTKEELEELMSDIKKTANKVRSKLKSIEQSIEQEEGLNRS----SADLRIRKTQHSTLSRKFV
*N.vectensis* 87 NIDKIAQDVERVKKAHSAVLSSAVPDQEVKDNLEICMSRIQKTANTVRSRIKAMEQQIKEDEKQGGSLHNNYAEARIKKCQHATLSRKFI


*T.diomedea* 133 EVMNDYNACQIDYRERCKGRIKRQLAITDRSITNEELEDMIESGNPAIFTQGIIMETQQAKQTLADIEARHNDIMKLETSIRDLHDMFMD
*M.leonina* 133 EVMNDYNACQIDYRERCKGRIKRQLAITDRSITNEELEDMIESGNPAIFTQGIIMETQQAKQTLADIEARHNDIMKLETSIRDLHDMFMD
*A.californica* 131 EVMNDYNACQIDYRERCKGRIKRQLAITGKTTTNEELEDMIESGNPAIFTQGIIMETQQANETLADIEARHNDIMKLETSIRDLHDMFMD
*L.stagnalis* 131 EVMNDYNACQIDYRERCKGRIKRQLAITGKTTTNEELEDMIESGNPAIFTQGIIMETQQAKQTLADIEARHNDIMKLETSIRDLHDMFMD
*D.melanogaster* 132 EVMTEYNRTQTDYRERCKGRIQRQLEITGRPTNDDELEKMLEEGNSSVFTQGIIMETQQAKQTLADIEARHQDIMKLETSIKELHDMFMD
*C.elegans* 130 EVMTDYNKTQTDYRERCKGRIQRQLDIAGKQVGDEDLEEMIESGNPGVFTQGIITDTQQAKQTLADIEARHNDIMKLESSIRELHDMFMD
*H.sapiens* 129 EVMSEYNATQSDYRERCKGRIQRQLEITGRTTTSEELEDMLESGNPAIFASGIIMDSSISKQALSEIETRHSEIIKLENSIRELHDMFMD
*N.vectensis* 177 EVMSEYNTTQTEYRELCKARICRQLEITGKSKTSEEVEDMLESGNPSIFTSDIVIQTQQAKQALGDIEARHRDIITLEKNIQELHEMFQD


*T.diomedea* 223 MAMLVESQGEMIDRIEYNVEQAVDYIETAKMDTKKAVKYQSKARRKKIMIIICLIILV--IIIVGIVGGVFA
*M.leonina* 223 MAMLVESQGEMIDRIEYNVEQAVDYIETAKMDTKKAVKYQSKARRKKIIIFICLIVLI--IIVVGIVGGVFA
*A.californica* 221 MAMLVESQGEMIDRIEYNVEQAVDYIETAKMDTKKAVKYQSKARRKKIMILVCLAILI--IILVGVIGGTLG
*L.stagnalis* 221 MAMLVESQGEMIDRIEYNVEQAVDYIETAKMDTKKAVKYQSKARRKKIMIIICVCVLI--IILVGILGGTFG
*D.melanogaster* 222 MAMLVESQGEMIDRIEYHVEHAMDYVQTATQDTKKALKYQSKARRKKIMILICLTVLG--ILAASYVSSYFM
*C.elegans* 220 MAMLVESQGEMVDRIEYNVEHAKEFVDRAVADTKKAVQYQSKARRKKICILVTGVILITGLIIFILFYAKVL
*H.sapiens* 219 MAMLVESQGEMIDRIEYNVEHAVDYVERAVSDTKKAVKYQSKARRKKIMIIICCVILG--IVIASTVGGIFA
*N.vectensis* 267 MYMLVESQGEMIDRIEFNVEQAVDYVQSAKTDTKKALTYQSKARRKKILIIICCLILLA-IIIGAIVGALNG


**Figure S9. MUSCLE protein alignment of syntaxin homologues from *Tritonia diomedea*, *Melibe leonina*, *Aplysia californica*, *Lymnaea stagnalis*, *Drosophila melanogaster*, *Caenorhabditis elegans*, *Homo sapiens* and *Nematostella vectensis*.**
