# Supplementary material for: Deep mRNA Sequencing of the Tritonia diomedea Brain Transcriptome Provides Access to Gene Homologues for Neuronal Excitability, Synaptic Transmission and Peptidergic Signalling
Source: PLoS One. 2015 Feb 26;10(2):e0118321. doi: 10.1371/journal.pone.0118321 (PMC4342343; doi:10.1371/journal.pone.0118321)
Supplement: S10 Fig — (DOCX) [file pone.0118321.s011.docx]

*T.diomedea* 1 MNIVYDASDEGEEDAMAGDDASARLRHKARNMIGATGSRHINQKRRSSLAAGTPLPLSLVSNLEQLSESDESRMANVP-RRRVVDTSDLR

*M.leonina* 1 MNIVYDASDEGEEDGIPSDEASARLRHKARNMIGATGSRHMNQKRRSSLATGTTLPLTLVPNLEQVSEMDEAGMAYVP-RRRVVDTSDLR

*L.stagnalis* 1 ------------------------------------------------------------------------------------------

*D.melanogaster* 1 ------------------------------------------------------------------------------------------

*C.elegans* 1 MCIRLKYLKSLFFLERDSTTFDQRSAARRASVLGRSAAESLSAQEAASSSERGEHTNSRSPSTSYSSCIDDERKFSSPHRRRVVDVSDHK

*H.sapiens*_Cav2.2 1 ------------------------MVRFGDELGGRYGGPGGGERARGGGAGGAGGP----------------------------------

*A.millepora* 1 ------------------------------------------------------------------------------------------

*T.diomedea* 90 TCAILQSRLKEL-KTYPEMATFQVSNSLQD--DDSGLYDGPFGTLSRKAALLGLPGMATQ----S---TRSLFIFSEENFIRKYAKIIIE

*M.leonina* 90 TCAILQSRLKEL-KTYPEMATFQASNSLQD--DDLGGFDGPFGTLSRKAALLGLPGMATQ----S---TRSLFIFSEENFIRKYAKIIIE

*L.stagnalis* 1 ------------------MATFQANNGQQDDGDNTTNQDGPFSHFSRKAALLGLPGMASQ----S---TRSLFIFSEENFIRKYAKIIIE

*D.melanogaster* 1 ---------------------------------------------------MGGPKKEENPPGGG---PTSLFILTEDNPIRKYTRFIIE

*C.elegans* 91 TCALLMTRMKEASRQLPSPSQLAAEEARREQKAESG-------TFVRKTTLSSNAPVKEK----G---PSSLFIFAEDNIIRRNAKAIIE

*H.sapiens*_Cav2.2 33 -----------------------GPGGLQP--GQRVLYKQSIAQRARTMALYNPIPVKQN----CFTVNRSLFVFSEDNVVRKYAKRITE

*A.millepora* 1 ---------MEQ-NGYPRANFTSATKSLWPNGTDLSQYKSRLNGHATKYTKPGASAKRQKKSGNAVRPKRALLCLSLGNPIRSAAINLVE

*T.diomedea* 170 WGPFEYMVLLTIIANCIVLALEQHLPELDKTPLALQLDDTEVYFLGIFCVEAFLKVVALGFVLHKGSYLRNVWNIMDFVVVVTGF-ITLF

*M.leonina* 170 WGPFEYMVLLTIIANCIVLALEQHLPEQDKTPLALQLDDTEVYFLGIFCVEAFLKVVALGFVLHKGSYLRNVWNIMDFVVVVTGF-ITLV

*L.stagnalis* 66 WGPFEYMVLLTIIANCIVLALEEHLPSQDKTPLALQLDDTEVYFLGIFCVEAFLKIVALGFCLHKRSYLRNIWNIMDFIVVVTGF-ITLF

*D.melanogaster* 37 WPPFEYAVLLTIIANCVVLALEEHLPGGDKTVLAQKLEKTEAYFLCIFCVEASLKILALGLVLHKHSYLRNIWNIMDFFVVVTGF-MTQY

*C.elegans* 167 WGPFEYFILLTIIGNCVVLSMEQHLPKNDKKALSEWLERTEPYFMGIFCLECVLKVIAFGFALHKGSYLRSGWNIMDFIVVVSGV-VTML

*H.sapiens*_Cav2.2 94 WPPFEYMILATIIANCIVLALEQHLPDGDKTPMSERLDDTEPYFIGIFCFEAGIKIIALGFVFHKGSYLRNGWNVMDFVVVLTGILAT--

*A.millepora* 81 WKPFDVMILITIFANCAALAAYEPLPGRDSSEVNEGLEIAEYVFLAIFTLEAILKIIAYGFFFHSGAYLRNGWNILDFVIVVVGL-ATIL

*T.diomedea* 259 A-----SKGTGSPGAFNLRTLRAVRVLRPLKLVSGIPSLQVVLKSIIRAMAPLLQVCLLVLFAIVIFAIIGLEFYVGAFHSACFKTGKHS

*M.leonina* 259 A-----SKGSGSPGAFNLRTLRAVRVLRPLKLVSGIPSLQVVLKSIIRAMAPLLQVCLLVLFAIVMFAIIGLEFYVGIFHSACFKKDAQS

*L.stagnalis* 155 A-----Q---GSSTTFDLRTLRAVRVLRPLKLVSGIPSLQVVLKSIIRAMAPLLQVCLLVLFAIVIFAIIGLEFYVGVFHNACYKKGSHT

*D.melanogaster* 126 P-----Q----IGPEVDLRTLRAIRVLRPLKLVSGIPSLQVVLKSIIKAMAPLLQIGLLVLFAIVIFAIIGLEFYSGALHKTCY-----S

*C.elegans* 256 PFSPATQTANQPVDSVDLRTLRAVRVLRPLKLVSGIPSLQVVLKSILCAMAPLLQIGLLVLFAIIIFAIIGLEFYSGAFHSACY------

*H.sapiens*_Cav2.2 182 -----------AGTDFDLRTLRAVRVLRPLKLVSGIPSLQVVLKSIMKAMVPLLQIGLLLFFAILMFAIIGLEFYMGKFHKACF------

*A.millepora* 170 V------KALMSSGAFNVKALRAFRVLRPLRLVSGVPSLQVVLNSIIKALIPLFHIALLVVFVVIIYAIIGVELFMGRLHKTCY------

*T.diomedea* 344 FTEDDIDLGDE-EGIRPCSEEE---GFFSTFRCQNNVST-CQAEWRGPNYGITSFDHIGYAMLTVFQCITMEGWTDVLYYAIDSSGVVHI

*M.leonina* 344 YTEDDIDLGDE-EDIRPCSAEN---SFFSTFKCQKNVSE-CLPEWRGPNYGITSFDHIGYAMLTVFQCITMEGWTDVLYYTNDAYGEVNI

*L.stagnalis* 237 RSEDDIDTGDE-DDIRPCLPSS---ESQGAFQCQVNISN-CKAGWRGPNAGITSFDNIGYAMLTVFQCITMEGWTNVLYYTNDALGNQFN

*D.melanogaster* 202 LEDPNKLVKEG-ESETPCNTDNILEKATGSFVCNNTTSM-CLEKWEGPNSGITSFDNIGFAMLTVFQCITMEGWTAILYWTNDALGSAFN

*C.elegans* 340 -NERGEIENVS-ERPMPCTNKT---SPMGVYNCDVKGTT-CLQKWIGPNYGITSFDNIGFAMITVFQCITMEGWTTVMYYTNDSLGSTYN

*H.sapiens*_Cav2.2 255 ---PNSTDAEP-VGDFPCGKEA------PARLCEGD-TE-CREYWPGPNFGITNFDNILFAILTVFQCITMEGWTDILYNTNDAAGNTWN

*A.millepora* 248 ---NNITGAEAMENPHPCSSGG------SGFHCNASEAQVCEAGWKGPNYGITNFDNIALACMTVFQCITLEGWTDVLYMINDAVGNSWP

*T.diomedea* 429 F-YFMPLIILGSFFMLNLVLGVLSGEFAKERERVENRRAFFKLRRQQQIERELNGYLEWICKAEEVILSEERTTDEEKLKIIEARRQAAA

*M.leonina* 429 F-YFMPLIILGSFFMLNLVLGVLSGEFAKERERVENRRAFFKLRRQQQIERELNGYLEWICKAEEVILSEERTTDEEKLKIIEARRQAAA

*L.stagnalis* 322 FLYFIPLIILGSFFMLNLVLGVLSGEFAKERERVENRRAFFKLRRQQQIERELNGYLEWICKAEEVILSEERTTDEEKLKIIEARRQAAA

*D.melanogaster* 290 WIYFVPLIVIGSFFMLNLVLGVLSGEFSNERNRVERRMEFQKCRFRAMFQTAMVSYLDWITQAEEVILAEERTTEEEKMHIMEARRRNAA

*C.elegans* 424 WAYFIPLIVLGSFFMLNLVLGVLSGEFAKERERVENRREFLKLRRQQQIERELNGYLEWILTAEEVILKEDRTTEEEKAAIMEARRRAAN

*H.sapiens*_Cav2.2 333 WLYFIPLIIIGSFFMLNLVLGVLSGEFAKERERVENRRAFLKLRRQQQIERELNGYLEWIFKAEEVMLAEEDRNAEEKSPLDVLKRAAT-

*A.millepora* 329 WIYFVTLIIWGSFFVLNLVLGVLSGEFAKEKARAQKSGEFQKFREKQQVEDAYNGYLDWITQAEDIEGDSESETGDESKSSRRASRHS--

*T.diomedea* 518 RKMKQL---KEEDTDNDNEQNDNDLLAEITP-GNTFSKNLKSRRTNGKCANFWRAEKRFRYSCRR----LVKSQAFYWIVIVLVFLNTIS

*M.leonina* 518 RKMKQL---KEEDTDIDNEHNDNDLLAEITP-GNTFTKNLKSSRTNGKFANFWRAEKRFRYSCRR----LVKSQTFYWIVIVLVLLNTVS

*L.stagnalis* 412 RKMKQL---KAEDNENDSEQNDNDLLAAMAP-GNSF-KSMKKRRTTGKCASFWRAEKRFRYSIRR----LVKSQLFYWIVIVLVFLNTAS

*D.melanogaster* 380 KRKKLKSLGKSKSTDTEEEEAEEDYGDDGYL--------KTRSKPQGSCTGFWRAEKRFRFWIRH----TVKTQWFYWFVIVLVFLNTVC

*C.elegans* 514 KKLKQASKQQSTETEEDFEEDEDEMEEEYVDEGGTVEDEFAERKKRGCCHSVGKFIKQLRIQIRI----MVKTQIFYWSVITLVFLNTCC

*H.sapiens*_Cav2.2 422 ---------KKSRNDLIHAEEGEDRFADLCAVGSPFARASLKSGKTESSSYFRRKEKMFRFFIRR----MVKAQSFYWVVLCVVALNTLC

*A.millepora* 417 ---------RIDDIEMIDKNERQEITVQEAH--------------HGWCHNEKKVLKRWHHRTRRELRKAVKTQAFYWIVIVVVFLNSLT

*T.diomedea* 600 VASEHYNQPDWHTQFLYITEYAFLGLFLFEMSFKMYALGARIYFQSSFNIFDCVVIVGSIVEVIWSEFKKGSSFGISVLRALRLLRIFKV

*M.leonina* 600 VASEHYNQPDWHTQFLYITEYAFLGLFLFEMSLKMYALGARIYFQSLFNIFDCVVIVGSIFEVIWSEFKKGSSFGFSVLRALRLLRIFKV

*L.stagnalis* 493 VASEHYNQPEWHVQFLYITEYAFLGLFIFEMSIKMYALGVRMYFQSSFNIFDCVVIVGSIVEVIWSEFKRGSSFGISVLRALRLLRIFKV

*D.melanogaster* 458 VAVEHYGQPSFLTEFLYYAEFIFLGLFMSEMFIKMYALGPRIYFESSFNRFDCVVISGSIFEVIWSEVK-GGSFGLSVLRALRLLRIFKV

*C.elegans* 600 VASEHYGQPQWFTDFLKYAEFVFLGIFVVEMLLKLFAMGSRTYFASKFNRFDCVVIVGSAAEVIWAEVY-GGSFGISVMRALRLLRIFKL

*H.sapiens*_Cav2.2 499 VAMVHYNQPRRLTTTLYFAEFVFLGLFLTEMSLKMYGLGPRSYFRSSFNCFDFGVIVGSVFEVVWAAIKPGSSFGISVLRALRLLRIFKV

*A.millepora* 484 LALEHYGQPHFLTIFLDIANKLFLGIFTVEMLIKMYCLGIHGYFASLFNRFDCLVVVSSLLELAIVEAMSQRPIGISVLRCIRLLRIFKV

*T.diomedea* 690 TRYWASLRNLVISLLSSMRSILSLLFLLFLFILIFALLGMQLFGGEMNFE-EGRPSANFDTFPIALLTVFQILTGEDWNAVMYNGIRAHG

*M.leonina* 690 TRYWASLRNLVISLLSSMRSILSLLFLLFLFILICALLGMQLFGGEMNFE-EGKPSANFDTFPIALLTVFQILTGEDWNAVMYHGIRAHG

*L.stagnalis* 583 TRYWSSLRNLVISLLSSMRSILSLLFLLFLFIIVFALLGMQLFGGEMNFE-EGRPSAHFDTFPIALLTVFQILTGEDWNEVMYNGIKSHG

*D.melanogaster* 547 TKYWSSLRNLVISLLNSMRSIISLLFLLFLFILIFALLGMQLFGGQFNLP-GGTPETNFNTFPIALLTVFQILTGEDWNEVMYQGIISQG

*C.elegans* 689 TSYWVSLRNLVRSLMNSMRSIISLLFLLFLFILIFALLGMQLFGGRFNFP-TMHPYTHFDTFPVALITVFQILTGEDWNEVMYLAIESQG

*H.sapiens*_Cav2.2 589 TKYWSSLRNLVVSLLNSMKSIISLLFLLFLFIVVFALLGMQLFGGQFNFQ-DETPTTNFDTFPAAILTVFQILTGEDWNAVMYHGIESQG

*A.millepora* 574 TRYWSSLSNLVASLLNSMRSIAGLLLLLSLFMLICSLLGMQIFGGRFSMDGEDVPRSNFDSFWKALITVFQILTGEDWNAVMYDGIRSWG

*T.diomedea* 779 GIDDGGMFFCV-YFIVLVLIGNYTLLNVFLAIAVDNLANAQELTAAEEEQVEDEAMRR-EELEKEMAE----------------------

*M.leonina* 779 GIEGQGMVFSL-YFIVLVLIGNYTLLNVFLAIAVDNLANAQELTAAEEEQVEEEALRR-EELEKEMAE----------------------

*L.stagnalis* 672 GIENQGMFYSS-YFIVLVLFGNYTLLNVFLAIAVDNLANAQELTAAEEEQEEEEAVRR-EEIEKEMAE----------------------

*D.melanogaster* 636 GAQK-GMIYSI-YFIVLVLFGNYTLLNVFLAIAVDNLANAQELTAAEEEQVEEDKEKQLQELEKEMEA----------------------

*C.elegans* 778 GIYSGGWPYSI-YFIVLVLFGNYTLLNVFLAIAVDNLANAQELTAAEEADEKANEI---EEESEELDE----------------------

*H.sapiens*_Cav2.2 678 GVSK-GMFSSF-YFIVLTLFGNYTLLNVFLAIAVDNLANAQELTKDEEEMEEAANQKLALQKAKEVAEVSPMSAANISIAARQQNSAKAR

*A.millepora* 664 GIGEGGAILAILYFIFLVVVGNYILLNVFLAIAVDNLADAENLTEMEEEKKKKKEKAK-EKLRASTES----------------------

*T.diomedea* 845 --------------------------------QFSAQQQGRPPLVN---------------------------------ICPPSPQNNED

*M.leonina* 845 --------------------------------QFSAAQQGRPPMVN---------------------------------ICPPSPQNNEE

*L.stagnalis* 738 --------------------------------QFAA--QGRPPLVN---------------------------------ICPPSPQNNEE

*D.melanogaster* 702 ------------------------------------------------------------------------------------------

*C.elegans* 842 --------------------------------QYQ-------------------------------------------------------

*H.sapiens*_Cav2.2 766 SVWEQRASQLRLQNLRASCEALYSEMDPEERLRFATTRHLRPDMKTHLDRPLVVELGRDGARGPVGGKARPEAAEAPEGVDPPRRHHRHR

*A.millepora* 731 --------------------------------QTKIGQDGA--------------------------------------IVPHHSSATHS

*T.diomedea* 870 C-KTANFNYAGNRVDINLSQN---------------------------------------------------------------------

*M.leonina* 870 CNKTANFNYAGNRIDINMSQN---------------------------------------------------------------------

*L.stagnalis* 761 N-KTANFNYAGNRVDINLSQN---------------------------------------------------------------------

*D.melanogaster* 702 ------------------------------------------------------------------------------------------

*C.elegans* 845 ------------------------------------------------------------------------------------------

*H.sapiens*_Cav2.2 856 DKDKTPAAGDQDRAEAPKAESGEPGAREERPRPHRSHSKEAAGPPEARSERGRGPGPEGGRRHHRRGSPEEAAEREPRRHRAHRHQDPSK

*A.millepora* 751 N-----------------------------------------------------------------------------------------

*T.diomedea* 890 ----------------------------------------------------------------------NLKDTRDKKL--DNMATT--

*M.leonina* 891 ----------------------------------------------------------------------NLKDTRDKKL--DNIST---

*L.stagnalis* 781 ----------------------------------------------------------------------NLKDNRDKKISVDNVLETAA

*D.melanogaster* 702 -------------------------------------------------------------------------------LQADGVHVE--

*C.elegans* 845 ------------------------------------------------------------------------------------------

*H.sapiens*_Cav2.2 946 ECAGAKGERRARHRGGPRAGPREAESGEEPARRHRARHKAQPAHEAVEKETTEKEATEKEAEIVEADKEKELRNHQPREPHCDLETSGTV

*A.millepora* 752 ----------------------------------------------------------------------MTLDKSNQELHSAGNLNGNA

*T.diomedea* 906 -KGSSVTTPLA--RNNDDDMSDT--------------------------------ASTTSNATVDLQPRTNSQNE---GGGF-QGPKPML

*M.leonina* 906 -KGPSVMTPLA--RNNDDDMSDT--------------------------------ASTTSNATVDLQPRTNTQNE---GGGFQQGPKPML

*L.stagnalis* 801 -KTSSVTMPLA--NNTEEDMSDT--------------------------------ASTTSSGTADVQPRASSQND---NGGF-HGPKPML

*D.melanogaster* 711 -NGDGAVAPSK--SK--------------------------------------------------GKKKEEEKKE---EEEVTEGPKPML

*C.elegans* 845 -EGDHCTIDME--GKTAGDMC----------------------------------AVARAMDDLD----EECEEE---ESPF-GGPKPMV

*H.sapiens*_Cav2.2 1036 TVGPMHTLPSTCLQKVEEQPEDADNQRNVTRMGSQPPDPNTIVHIPVMLTGPLGEATVVPSGNVDLESQAEGKKEVEADDVMRSGPRPIV

*A.millepora* 772 VAQTASHSDIE--AQSVEQLEP---------------------------------EDSKSAVNNNEESAAVGSTE---DIDY----TPMP

*T.diomedea* 957 PYSSMFIFGPTNPIRRFCHFVVNLRYFDLFIMIVISASSVALAAEDPVVEESYRNRILHYFDYVFTGVFTIELILKVIDLGIILHPGSYI

*M.leonina* 958 PYSSMFIFGPSNPIRRFCHFVVNLRYFDLFIMIVILASSLALAAEDPVVEESYRNKILHFFDYVFTGVFTIELILKVIDLGIILHPGSYT

*L.stagnalis* 852 PYSSMFIFGPTNPIRRFCHFVVNLRYFDLFIMIVICASSVALAAEDPVIENSRRNEILNYFDFVFTGVFTIELVLKVIDLGVLLHPGSYI

*D.melanogaster* 745 PYSSMFILSPTNPIRRGAHWVVNLPYFDFFIMVVISMSSIALAAEDPVRENSRRNKILNYFDYAFTGVFTIEMLLKIVDLGVILHPGSYL

*C.elegans* 890 PYSSMFFLSPTNPFRVLIHSIVCTKYFEMMVMTVICLSSVSLAAEDPVDEENPRNKVLQYMDYCFTGVFACEMLLKLIDQGILLHPGSYC

*H.sapiens*_Cav2.2 1126 PYSSMFCLSPTNLLRRFCHYIVTMRYFEVVILVVIALSSIALAAEDPVRTDSPRNNALKYLDYIFTGVFTFEMVIKMIDLGLLLHPGAYF

*A.millepora* 820 PESALFIFSSTNIIRIICYRIATNKYFVNFVLVLIIVSSILLAVEDPLNASAERNQVLNYFDYFFTSVFTLEILIKFVAYGLILHKGSFC

*T.diomedea* 1047 RDLWNILDATVVFCALVAFAFKSINSDSAGKNLNTIKSLRVLRVLRPLKTINRVPKLKAVFDCVLNSLKNVSNILIVYLLFQFIFAVIAV

*M.leonina* 1048 RDLWNILDATVVFCALVAFAFNAIDSDSAGKNLNTIKSLRVLRVLRPLKTIKRVPKLKAVFDCVLNSVKNVSNILIVYFIFQFIFAVIAV

*L.stagnalis* 942 RDLWNILDATVVICALVAFVFKD-KSDSAGKNLNTIKSLRVLRVLRPLKTINRVPKLKAVFDCVVNSLKNVSNILIVYILFQFIFAVIAV

*D.melanogaster* 835 REFWNIMDAVVVICAAVSFGF-DMSGSSAGQNLSTIKSLRVLRVLRPLKTIKRVPKLKAVFDCVVNSLKNVVNILIVYILFQFIFSVIGV

*C.elegans* 980 RDFWNILDGIVVTCALFAFGFAG-TEGSAGKNLNTIKSLRVLRVLRPLKTIKRIPKLKAVFDCVVNSLKNVFNILIVYFLFQFIFAVIAV

*H.sapiens*_Cav2.2 1216 RDLWNILDFIVVSGALVAFAFSG----SKGKDINTIKSLRVLRVLRPLKTIKRLPKLKAVFDCVVNSLKNVLNILIVYMLFMFIFAVIAV

*A.millepora* 910 RSAFNLLDLLVVSVSVISISLKN-------SQFSVVRILRVLRVLRPLRAINRAKGLKHVVQCVFVAVKTIWNIMLVTMLFNFLFAVIGV

*T.diomedea* 1137 QLFKGRFFYCTDESKSTREECQGQFFEYDGSS-DQPTVKDREWLRQSFHYDDIINAMLTLFTVTTGEGWPSVLKHSMDSTYENMGPKPGS

*M.leonina* 1138 QLFKGRFFFCTDESKSTREECQGQFFEYDGSS-DQPTIRDRQWLRQDFHYDDIVNAILTLFTVTTGEGWPSVLKHSMDSTYEDMGPKPGS

*L.stagnalis* 1031 QLFKGRFFYCTDESKSTRDECQGQFFEYDGHS-NDPTVRDREWLRQDFHYDNIMMAMLTLFTVTTGEGWPSVLKHSMDSTYEDRGPKPVY

*D.melanogaster* 924 QLFNGKFFYCTDESKHTSAECQGSYFKYEEDE-LLPKQELRVWKPRAFHYDNVAAAMLTLFAVQTGEGWPQVLQHSMAATYEDRGPIQNF

*C.elegans* 1069 QLFNGKFFFCTDKNRKFANTCHGQFFVYDNQN-DPPRVEQREWRLRPFNYDNTINAMLTLFVVTTGEGWPGIRQNSMDTTFEDQGPSPFF

*H.sapiens*_Cav2.2 1302 QLFKGKFFYCTDESKELERDCRGQYLDYEKEE-VEA--QPRQWKKYDFHYDNVLWALLTLFTVSTGEGWPMVLKHSVDATYEEQGPSPGY

*A.millepora* 993 QLWKGTFFYCTDQKKRFEDECKGEYFEYNGAGLSNPVAKKREWKRRDFNFDNVGNAMLTLFTVMTFEGWPGILYNSIDSTEVDEGPLQNN

*T.diomedea* 1226 RMEMAIFYVVFFIVFPFFFVNIFVALIIITFQEQGENELMDLEIDKNQKQCADFAINAKPLCRFMPKNKNSIKYKIWKLVQSPKFEYFIM

*M.leonina* 1227 RMYMATFYVVFFIVFPFFFVNIFVALIIITFQEQGENELMDLEIDKNQKQCVDFAINAKPLCRFMPKNKNSTKYKIWKLVQSPKFEYVIM

*L.stagnalis* 1120 RMEMSLFYVVFFIVFPFFFVNIFVALIIITFQEQGENELMDQEMDKNQKQCIDFAINAKPHCRFIPKNKNSIKYKIWRLVQSSKFEYFVM

*D.melanogaster* 1013 RIEMSIFYIVYFIVFPFFFVNIFVALIIITFQEQGEAELQDGEIDKNQKSCIDFTIGARPLERYMPKNRNTFKYKVWRIVVSTPFEYFIM

*C.elegans* 1158 RVEVALFYVMFFIVFPFFFVNIFVALIIITFQEQGEAELSEGDLDKNQKQCIDFALNARPRSLFMPEDKNSTKYRIWRLVTSPPFEYFIM

*H.sapiens*_Cav2.2 1389 RMELSIFYVVYFVVFPFFFVNIFVALIIITFQEQGDKVMSECSLEKNERACIDFAISAKPLTRYMPQNRQSFQYKTWTFVVSPPFEYFIM

*A.millepora* 1083 RPWVAVYYIIYIIIIAFFMVNIFVGFVIVTFQSEGEEEFKDCELDKNQRQCIEFALKAKPFRRYIPENR--LQFHIWRVVTSQPFEYLIF

*T.diomedea* 1316 TLIALNTIVLMMKFDPRLVSHIDEGETFGDHLNYINIAFTVLYTLEFLLKVTAFG-KNYFHDAWNAFDFVTVLGSIVDAFITEISD--AR

*M.leonina* 1317 TLIALNTIVLMMKFDSKQYTMKSKGTSFSDHLNYVNIGFTVLYTTEFLLKVTAYG-KNYFHDAWNVFDIITVLGSIVDAFITGISD--SR

*L.stagnalis* 1210 TLITLNTIVLMMKYD-------GMSDNYKDVLAKLNEGFTVLFTLECLLKIIGLGPRNYFHDPWNVFDFTTVVGSIIDVLITEFSK--RQ

*D.melanogaster* 1103 MLIVFNTLLLMMKYH-------NQGDMYEKSLKYINMGFTGMFSVETVLKIIGFGVKNFFKDPWNIFDLITVLGSIVDALWMEFGHDSNS

*C.elegans* 1248 TMICCNTLILMMKYY-------NNPLFYEEILRLFNTALTAVFTVESILKILAFGVRNYFRDGWNRFDFVTVVGSITDALVTEFGG--HF

*H.sapiens*_Cav2.2 1479 AMIALNTVVLMMKFY-------DAPYEYELMLKCLNIVFTSMFSMECVLKIIAFGVLNYFRDAWNVFDFVTVLGSITDILVTEIAETNNF

*A.millepora* 1171 AFIVCNTVVLMMQYY-------QEPRLYTRVLDGFNIGFTAVFLLECILKLVAFKPKNYFIDRWNLFDFIIVVGSIIDITMNEVSSE-QM

*T.diomedea* 1403 FSFGFFRLFRAARLVKLLRQGYTIRLLLWTFFQSFKALPYVCLLILIIFFIFAIIGMQVFGSIKLDS-RTAINRHNNFRNFFQALVLLFR

*M.leonina* 1404 FSFGFFRLFRAARLVKLLRQGYTIRLLLWTFFQSFKALPYVCLLILIMFFIFAIIGMQVFGSIKLDS-RTAINRHNNFRTFFQALVLLFR

*L.stagnalis* 1291 VSFGFFRLFRAARLVKLLRQGYTIRLLLWTFFQSFKALPYVCLLILMLFFIYAIIGMQVFGSIKLDS-KTSINRHNNFRTFFSALTLLFR

*D.melanogaster* 1186 INVGFLRLFRAARLIKLLRQGYTIRILLWTFVQSFKALPYVCLLIAMLFFIYAIIGMQVFGNIKLGTVENSITRHNNFQSFIQGVMLLFR

*C.elegans* 1329 VSLGFLRLFRAARLIRLLQQGYTIRILLWTFVQSFKALPYVCLLIGMLFFIYAIVGMQVFGNIWLNA-ATEINRHNNFQSFFNAVILLFR

*H.sapiens*_Cav2.2 1562 INLSFLRLFRAARLIKLLRQGYTIRILLWTFVQSFKALPYVCLLIAMLFFIYAIIGMQVFGNIALDD-DTSINRHNNFRTFLQALMLLFR

*A.millepora* 1253 FAFGFFRLFRALRLVKLLNQGSGIKTLLWTFIKSFQALPYVALLIVMMFFIYAVIGMQMFGRIALHP-ETAINRNNNFQTFPHSLMVLFR

*T.diomedea* 1492 CATGESWQLIMLSC--RDGRPCDPESLRPD---DPADMGESGCGSNIAYVYFVSFIFLMSFLMLNLFVAVIMDNFDYLTRDSSILGPHHL

*M.leonina* 1493 CATGESWQLIMLSC--RAGRPCDPGSIREDGDEDPSETAEYGCGSDFAYVYFLAFIFLMSFLLLNLFVAVIMDNFDYLTRDSSILGPHHL

*L.stagnalis* 1380 CATGEAWQQIMQSC--LAGQPCDPESIRDD---DPPDMAESGCGTNIAYMYFVSFIFLCSFLMLNLFVAVIMDNFDYLTRDSSILGPHHL

*D.melanogaster* 1276 CATGEAWPNIMLAC--LKGKACDD---------DAEKAPGEYCGSTLAYAYFVSFIFFCSFLMLNLFVAVIMDNFDYLTRDSSILGAHHL

*C.elegans* 1418 CATGEGWQDIMMAA--VQGKDCARAGSA-----EINFEKGQTCGSNVSYAYFTSFVFLSSFLMLNLFVAVIMDNFDYLTRDSSILGPHHL

*H.sapiens*_Cav2.2 1651 SATGEAWHEIMLSC--LSNQACDE------------QANATECGSDFAYFYFVSFIFLCSFLMLNLFVAVIMDNFEYLTRDSSILGPHHL

*A.millepora* 1342 SATGENWQEIMLSCTNREDVKCDPNA-------DPKDPSGL-CGSDFAYFYFVSFYSICSFLIINLFVAVIMDNFDYLTRDWSILGPHHL

*T.diomedea* 1577 DEYVRVWSNYDPGATGRIHYTDMYEMLRNMEPPVGFGKKCPYKLAYRKLIRMNMPVA-EDGTVHFTTTLFALVRECLMIKMGPA-ENMDK

*M.leonina* 1581 DEYVRVWSNYDPGATGRIHYTDMYEMLRNMEPPVGFGKKCPYKLAYRKLIRMNMPVA-EDGTVHFTTTLFALVRECLMIKMGPA-ETMDK

*L.stagnalis* 1465 DEYVRVWSMYDPKATGRIHYTDMYEMLRNMEPPVGFGKKCPYKLAYRKLIRMNMPVA-EDGTVHFTTTLFALIRECLIIKMGPA-EIMDR

*D.melanogaster* 1355 DEFVRIWAEYDPNATGKIHYTEMYDMLKNMDPPLGFGNKCPNRLAYKKLIRMNMPLD-DELRVQFTTTLFALIRENLSIKMRAP-EEMDQ

*C.elegans* 1501 DEFIRVWADYDPAATGRIHYSEMYEMLRIMAPPVGFGKKCPYRLAYKHLIRMNMPVA-EDGTVHFTTTLFALIRESLSIKMRPV-EEMDE

*H.sapiens*_Cav2.2 1727 DEFIRVWAEYDPAACGRISYNDMFEMLKHMSPPLGLGKKCPARVAYKRLVRMNMPISNEDMTVHFTSTLMALIRTALEIKLAPAGTKQHQ

*A.millepora* 1424 DEYVRVWSEYDPEARGCIKHVDIVTLLKRIAPPLGFGKFCPHREACKRLVTMNMPLT-KDGMVDFNATLFGLVRSSLNIKKPEGKGSIDK

*T.diomedea* 1665 KDEEMRESIRKLWPVQGKK-MMDMLMPPVDELDEGKMSVGKIYAGLLVSENWKAYKASQNA-----SNNFKMRP-SLF------------

*M.leonina* 1669 RDDEMRDSIRKLWPVQGKK-MMDMLMPPVDELDEGKMSVGKIYAGLLVSENWKAYKASQNA-----SNNFKMRP-SLF------------

*L.stagnalis* 1553 RDEEMRETIRKLWPVQGKK-MMDMLMPPSDELDEGKMSVGKIYAGLLISENWKAYKASQNA-----SNNFKMRP-SLF------------

*D.melanogaster* 1443 ADMELRETITNIWPLQAKK-MLNLLVPPSDQLNKGKLSVGKIYAGFLILESWRSTRFGQLD-----SG--MPKQ-SFF------------

*C.elegans* 1589 ADEELRLTLKKIWPLKAKKNMVDLVVPPNHELCFQKLTVGKIYAGLLILENYRARKSGTEV-----GGQ------GLF------------

*H.sapiens*_Cav2.2 1817 CDAELRKEISVVWANLPQK-TLDLLVPPHKP---DEMTVGKVYAALMIFDFYKQNKTTRDQMQQAPGGLSQMGPVSLFHPLKATLEQTQP

*A.millepora* 1513 ANGEVRNIILRIWPKTSMQ-LLDKVVQPPGVHDD--VTVGKFYATYLIQEYFRRFKARQKA-----QDQAEVPENNTM------------

*T.diomedea* 1736 ------RRLMGGGGGGGGGGGGGGGNRNSSARSSQSLESEHSDENEEE--------------------G----------------GHSF-

*M.leonina* 1740 ------RKLM---------GGGGKSMRNSSARSSQSFDSEHSNDNEEG--------------------G---QSIDKGDDWNPRATPTFS

*L.stagnalis* 1624 ------RRLM-------------GGMRTSSARSSQSLDSEQSDDNDGG--------------------GGGGGSANAGGGGGSSAGHSF-

*D.melanogaster* 1512 ------NCLL----------DMAALDKGGSRQGSISFEPNGEGAANSQ--------------------THLLASTHHHHANGDAEHNSLA

*C.elegans* 1656 --------------------GGGLRSLVAAAKAAESQHSSHTPQPPEETTPIIPQHAQQFSAAPTMSAQGSLQQMQGTSSGGGQRPYSLF

*H.sapiens*_Cav2.2 1903 AVLRGARVFLRQKSSTSLSNGGAIQNQESGIKESVSWGTQRTQDAPHE-------------------ARPPLERGHSTEIPVGRSGALAV

*A.millepora* 1583 -------------------------ALQAGLRTLHGLGPQ--------------------------------------------------

*T.diomedea* 1783 -LRRNSSKRRRDNGGNR-GDNSNVQPGQDFSKGLRPEHAGNLTNRDQTTRSGAHSPSLPPTPLSPRSPMGGQSPFGS-PRA-SPVPGRRS

*M.leonina* 1792 FLRRNSSKRRKDQGNNR-GDNSNVQPGQDFSRGLRPEHAGSLNARD-LARSGAHSPSLPPTPLSPRSPMGGQSPFGS-PRA-SPVPGRRS

*L.stagnalis* 1674 -LRRNSSKRRK--GGDH-GDNSNVQPGTDFSGGLRPEHASSLTSRG--DRAGARSPSLPPTPLSPRSPMGAQSPFGS-PRA-SPIPSRRS

*D.melanogaster* 1566 TLARRSTIRKRSVRNKK-MLELQDASRHPSQESLTGADAGHLHPGHSYMNGHRRSPSLRHN--------------GS-PLARSPSPRRRG

*C.elegans* 1726 NSFVDTIKSGKQDGDVT-DVQYQSVDQQHEKMNSTGRRLSDMFSKIRRGTSADHNPHQTEHLLAQDNRSPSSPRYRSMARASPPSPAERY

*H.sapiens*_Cav2.2 1974 DVQMQSITRRGPDGEPQPGLESQGRAASMPRLAAETQPVTDASPMKRSISTLAQRP--RGTHLCSTTPDRPPPSQASSHHHHHRCHRRRD

*A.millepora* 1598 -LRRAISGQLGED-----DDELFLK----EDASQKAQHKGFWESLKNAVSVSPRHSFRRPNSFRLSTFLGKN----------ESATEKKK

*T.diomedea* 1869 VSPRRFDVG------FAAAVTNLCEQAH--------SIADQDRQKKFGVKNEESVSAS--SP----------TYRGRSRHRSRPPLQSQS

*M.leonina* 1878 MSPRRFDVG------FAAAVTNLCEQAH--------TIADQDRHRKYGVKNEESASAS--SP----------TYRGRSRHRSRPPLQSQS

*L.stagnalis* 1756 PSPRRFDVG------FASAVANLCEQAH--------TIADQDRQKRYGVK-EDSITSS--SP----------TFRGRSRQRSRPPLQAQS

*D.melanogaster* 1640 HQYIHHDIG------FSDTVSNVVE------------MVKETRHPRHG--------NS--HP----------RYPRGSWSASTSPARSPS

*C.elegans* 1815 GHPPRYRTE--------SPPSSRSEYQM--------SIRDPIIRRNRYNTMEHSRSSH--DP----------QYHQDQQQQQQPHHQQHS

*H.sapiens*_Cav2.2 2062 RKQRSLEKGPSLSADMDGAPSSAVGPGLPPGEGPTGCRRERERRQERGRSQERRQPSS--SSSEKQRFYSCDRFGGREPPKPKPSLSSHP

*A.millepora* 1668 KSSSMSNLG-------ENRNANLVNNER--------FLAPSTSADENGNENESEAETSLMEP----------PPKGPEKEASELFKRDHA

*T.diomedea* 1933 PV-LGSPLPSPGYSRIRSSGNTGFYRSTSLETRSRSPSP---------------------NVAQSPPPYSSVRDV-RGSLTPSP--VAAS

*M.leonina* 1942 PI-LGSPLPSPGYSRMRSGGNTGFYRSTSLETRSRSPSP---------------------NIAPSPPPYTSVRDL-QGSRSPSP--IAAS

*L.stagnalis* 1819 PV-LGSPLPSPAHPRVRGGGDSGFYRSTSLETRSRSPSP---------------------NLTASPPPRSGSTSLVQRSRSPSPSLVAAS

*D.melanogaster* 1692 PSRYGGHLSRSKRTQLPYPT----YGTTSLCQRSRSPSPARLQEMRERDRLGYGIDMGVTHVQHSYPTLASRRAGIGRRLPPTPSKPSTL

*C.elegans* 1877 QH--------LQHSHHKTYQNHNQYSRSPIYSDDSSVAE----------------------------SYRREREF-RRYQDSTPQDVSED

*H.sapiens*_Cav2.2 2150 TS------PTAGQEPGPHPQGSGSVNGSPLLSTSGASTP----------------------------GRGGRRQLPQTPLTPRPSITYKT

*A.millepora* 1733 TIKAASSLPLTGDNRKIFGP------ITYLRQRSRSETL---------------------------PNRSSSEDEIHRLSHEDRSESARS

*T.diomedea* 1998 PP-MPK-RGARKLP-AAPSPSPGGGGQHQQP--HHQLQQHPQHLPPQNMPPPSSSSSSSPAKP--------ASLNLAEPRYREN--PKDA

*M.leonina* 2007 PPSMPKQRGARKLP-SAPSSSPGGSQHQPSPPQHHRDRHH--------------------------------------------------

*L.stagnalis* 1887 PP-MTS-SAHRRLP-VAPSSTSSSGSGVTTM---------------------------SPAKP--------VSLNLSEPRYRDNIAIKDT

*D.melanogaster* 1778 QL-KPTNINFPKLN-ASPTHTHHSTPHSVHSLPHHRDLLR------------------DPRDM--------YYSSRERERDRERLRDRDR

*C.elegans* 1930 DDPMPTAVRARRLPLISTMPTHYESAYQPSSYNQHLNDSYGLGTGYQRDYHTSHSHSHHPTSQQQQHQPMYSTSPLISPRSSHSYYTPRS

*H.sapiens*_Cav2.2 2206 ANSSPIHFAGAQTSLPAFSPGRLSRGLSEHNALLQRDPLSQPLAPGSR----------IGSDP--------YLGQRLDSEASVHALPEDT

*A.millepora* 1790 LIEEAMLDEGISITLDDP-LLRIAEQEIAEAFDVSEDDLHS---------------------------------------------AAER

*T.diomedea* 2073 LPVKAALSPSGGRGSNINFPRLSASPTRVPKLNIPVSASSSSAAASASAA----------------------------------------

*M.leonina* 2046 -------------------------HQRHPHS----------------------------------------------------------

*L.stagnalis* 1939 LPVRGAPSPPG--RGNINFPRLNASPTRVPKLNIPVSSTSGIAAASPLPPHHHRHAPPPGRLGRPEPYSPTERNNLNKTSDPSSSSARSS

*D.melanogaster* 1840 DRDRDRLHEYDLRYEYRDRERELYERERDREREVERERLEYIAPLSFEQA----------------------------------------

*C.elegans* 2020 SQYYEIPSPSPDIYPSY---RGSASPRRYPTSTVVVAPDREGSSARVIQA----------------------------------------

*H.sapiens*_Cav2.2 2278 LTFEEAVATNSGRSSRTSY-VSSLTSQSHPLRRVPNGYHCTLGLSSGGRA----------------------------------------

*A.millepora* 1834 MLADEDDRDSADVDSARHSPSVPFRLSGGPDSELVITDL---------------------------------------------------

*T.diomedea* ------------------------------------------------------------------------------------------

*M.leonina* ------------------------------------------------------------------------------------------

*L.stagnalis* 2027 TLPIAHRTSGYPRDDSNWGARELPGEGGRDSRSLPRPSPRASSRSPDPRGGSRHDDRFMAASQQEGSPARGTRSRGGILPNGFKPKGRKP

*D.melanogaster* 1890 -----------------------------------------------------------------LAMGRTGRVLPSPVLNGFKPKSGLN

*C.elegans* 2067 ------------------------------------------------------------------------------------------

*H.sapiens*_Cav2.2 2327 ------------------------------------------------------------------------------------------

*A.millepora* ------------------------------------------------------------------------------------------

*T.diomedea* ---------------------------

*M.leonina* ---------------------------

*L.stagnalis* 2117 EKYEMRSDSHTALQEDSDEDDDDWC--

*D.melanogaster* 1915 PRH-------------SDSDEEDWC--

*C.elegans* 2067 ------QPGSIPLSDSETEDDPRWAIV

*H.sapiens*_Cav2.2 2327 ------------RHSYHHPDQDHWC--

*A.millepora* ---------------------------

**Figure S10. MUSCLE protein alignment of voltage-gated Ca_v_2 channel homologues from *Tritonia diomedea*, *Melibe leonina*, *Aplysia californica*, *Lymnaea stagnalis*, *Drosophila melanogaster*, *Caenorhabditis elegans*, *Homo sapiens* (Ca_v_2.2 isotype) and *Nematostella vectensis*.**
