# Supplementary material for: Deep mRNA Sequencing of the Tritonia diomedea Brain Transcriptome Provides Access to Gene Homologues for Neuronal Excitability, Synaptic Transmission and Peptidergic Signalling
Source: PLoS One. 2015 Feb 26;10(2):e0118321. doi: 10.1371/journal.pone.0118321 (PMC4342343; doi:10.1371/journal.pone.0118321)
Supplement: S11 Fig — (DOCX) [file pone.0118321.s012.docx]

*T.diomedea* 1 --------------------------------------------------------------------------------------SAQSPDWSKLEGVFFSDLVNSPGK

*M.leonina* 1 -------------------------------------MSSNKEIASTLGHNPLAKTEGSLKNLTIEPRAVHSGKQPDSNGKNQESTSALSPDWSALEGVFYSDLNKAGEK

*L.stagnalis* 1 -----------------------MFLNCGVFQKSKVPRDITSEARLSSATSSPKKSPIKVGGMADKSFDVSSNQLSNDEYLNYKERDVTTNDWSDLEGVFAKDLDGPNGH

*D.melanogaster* 1 --------------------------------------------------------------------------------MSLNPNGYKLSERTGKLTAYDLMPTTVTAG

*C.elegans* 1 ----------------------------------------------------------------------------------MSSAAADESDAVLENLIAKEILPQTGNW

*H.sapiens* 1 MASSTPSSSATSSNAGADPNTTNLRPTTYDTWCGVAHGCTRKLGLKICGFLQRTNSLEEKSRLVSAFRERQSSKNLLSCENSDRDARFRRTETDFSNLFARDLLPAKNGE

*H.vulgaris* 1 -----------------------------------------------------------MSAISSMKKNRYVDKIEAMLTLQKPKIAIDHREHVVTKKEKLVRKKIHLKD

*T.diomedea* 25 SKTPEFLQSLCDMLTSYITIENDRSTKVLDFHHPHQLTEMMS-HCLDIHPDPRDLEQILSDCKETLKYCVKTGHPHFLNQLSTGVDVVGVAATWLTGTANTNLFTYEVAP

*M.leonina* 74 GKTVEFLSSMCQMLSNYLLSEKDRATKVLDFHHPHQLKEMMS-HCLDIHPEPRDLEQILSDCKETLKYCVRTGHPHFLNQLSTGVDFVGVAATWLTGTANTNLFTYEVAP

*L.stagnalis* 88 EIIRKFLTSLTEVLTTYVISEHDRTTKVLDFHHPHQLKEMMS-HCLDVHPEPRDLEQVLSDCKETLKYCVKTGHAHFFNQLSTGIDVVGMAGAWSSASANTNLFTYEAAP

*D.melanogaster* 31 PETREFLLKVIDVLLDFVKATNDRNEKVLDFHHPEDMKRLLD---LDVPDRALPLQQLIEDCATTLKYQVKTGHPHFFNQLSNGLDLISMAGEWLTATANTNMFTYEIAP

*C.elegans* 29 EGTEEFLNRIVQVLLKYIKDQNDRDQKILEFHHPDKMQMLMD---LSIPEKPESLLKLVKSCEDVLRLGVRTGHPRFFNQISCGLDLVSMAGEWLTATANTNMFTYEIAP

*H.sapiens* 111 EQTVQFLLEVVDILLNYVRKTFDRSTKVLDFHHPHQLLEGMEGFNLELSDHPESLEQILVDCRDTLKYGVRTGHPRFFNQLSTGLDIIGLAGEWLTSTANTNMFTYEIAP

*H.vulgaris* 52 NTYKGVVSKLCD----FIKSSNDRTSKVIHYIDPSQLQKEID---FNFKNEGENLQEILCITDKVLKYAVKTGHPRFFNQLFSGLDITCLMGQWISTTTNTLMFTYEVGP

*T.diomedea* 134 VFTLMEEVVLRRMRTLVGWTD--GNGIFAPGGAISNLYGMLLARHHMFPGAKENGFCEVT-RVSVFTSDHSHYSIKRAAAILGIGTNRVISVRCLENGKMDVTDLRHQME

*M.leonina* 183 VFTLMEEVVLRRMRSLVGWAE--GDGIFAPGGAISNLYGMLLARHRHFPDVKERGFLEAS-TVYVFTSDQCHYSIRRAAAILGIGLNHVISVRCLDSGKMDVCELRQQMG

*L.stagnalis* 197 VFTLMEEVILTRMRKMVGWDD--GEAIFAPGGAISNLYGVLLARHHTLPDVKQNGIHQGT-KPVVFTSEQSHFSIKRAAAILGIGTNNVVFIRCHPNGKMDVNDLRDKMM

*D.melanogaster* 138 VFILMENVVLTKMREIIGWSG--GDSILAPGGSISNLYAFLAARHKMFPNYKEHGSVGLPGTLVMFTSDQCHYSIKSCAAVCGLGTDHCIVVPSDEHGKMITSELERLIL

*C.elegans* 136 VFILMEKSVMARMWEAVGWDPEKADGIFAPGGAIANLYAMNAARHQLWPRSKHLGMKDIP-TLCCFTSEDSHYSIKSASAVLGIGADYCFNIPTDKNGKMIPEALEAKII

*H.sapiens* 221 VFVLMEQITLKKMREIVGWSSKDGDGIFSPGGAISNMYSIMAARYKYFPEVKTKGMAAVP-KLVLFTSEQSHYSIKKAGAALGFGTDNVILIKCNERGKIIPADFEAKIL

*H.vulgaris* 155 VYIMMEKYLLDKMKSIIGYSN--GDAQMFPGGSISNMEAMSIAKYHFHPNLKEEGLYGGK-QLVAFVSEEAHYSSDKAAATLGIGTNNLKKIKSDEKGKMIVKDLVEQIE

*T.diomedea* 241 ACQAQGGVPMFVNATCGTTVLGAFDPINDIADLCEEMGAWLHIDGAWGGAVLLSREFKH-LVNGIERADSMTWNPHKMMGVPLQCSAFLTKHKGLMRECNSMKAS---YL

*M.leonina* 290 ACRAKGGVPMFVNATCGTTVLGAFDPVADIADVCEEMGAWLHVDGAWGGAVLLSTEYRH-LAQGIERADSMTWNPHKMMGVPLQCSAFLTRHKGLMRDCNSLRAS---YL

*L.stagnalis* 304 TSQRSGHTILMVNATCGTTVLGAFDPISDIADLCEQHRVWLHLDAAWGGGAFLSRDHRY-LFTGAHRADSITWNPHKMMGAPLQCSAFITKHKGLLKNCNGMGAT---YL

*D.melanogaster* 246 ERKAKGDIPFFVNATAGTTVLGAFDDINTIADICQKYNCWMHIDAAWGGGLLMSRKHRHPRFTGVERADSVTWNPHKLMGALLQCSTIHFKEDGLLISCNQMSAE---YL

*C.elegans* 245 ECKKEGLTPFFACCTAGSTVYGAFDPLERVANICERHKLWFHVDAAWGGGMLLSPEHRY-KLAGIERANSVTWNPHKLMGALLQCSACLFRQDGLLFQCNQMSAD---YL

*H.sapiens* 330 EAKQKGYVPFYVNATAGTTVYGAFDPIQEIADICEKYNLWLHVDAAWGGGLLMSRKHRH-KLNGIERANSVTWNPHKMMGVLLQCSAILVKEKGILQGCNQMCAG---YL

*H.vulgaris* 262 ASLSRGEEPFFVCATAGTTVLGAFDPINDIADICKKYGLWLHVDGAWGGGSLLSRKYKH-LMAGVERADSVTWNPHKLMGCLFQCSILFTKKKDILASCNRESVDGASYL

*T.diomedea* 347 FQQDKN-YDV-SYDTGDITIQCGRHNDIFNLWLMWRAKGDIGFEQQVNKNFALAAYLRDSLIKRNG-FHLILS-KFEGPNICFWYLPLAWRSRAISDIKKSHLHMIAPTL

*M.leonina* 396 FQQDKN-YDV-TYDTGDVTIQCGRHNDIFNLWLMWRAKGDTGFETQVNRNFALAAYLRDMLVHKPG-FHLILT-KFEAPNICFWYLPLAWRSRQVKDIERAHLHTIAPTI

*L.stagnalis* 410 FQKDKV-YDT-SYDTGDMSIQCGRNNDIFKLWLMWRAKGDIGFEEQVKKNFQLAAYLRDKIKGRAG-FHLVLE-EIEAPNVCFWYLPIVWRPTPLNLVKPEHLAKIAPII

*D.melanogaster* 353 FMTDKQ-YDI-SYDTGDKVIQCGRHNDIFKLWLQWRAKGTEGFEQQQDRLMELVQYQLKRIREQSDRFHLIL--EPECVNVSFWYVPKRLRGVPHDAKKEVELGKICPII

*C.elegans* 351 FQQDKP-YDV-SFDTGDKAIQCGRHNDVFKLWLMWKSKGMEGYRQQINKLMDLANYFTRRIKETEG-FELIIE-NPEFLNICFWYVPSKIRNLEPAEMR-ARLEKIAPKI

*H.sapiens* 436 FQPDKQ-YDV-SYDTGDKAIQCGRHVDIFKFWLMWKAKGTVGFENQINKCLELAEYLYAKIKNREE-FEMVFNGEPEHTNVCFWYIPQSLRGVPDSPQRREKLHKVAPKI

*H.vulgaris* 371 FQKDKRLYNAKEWDQGDKTIQCGRNVDVLKLWLMWKAKGDKGMEEQIDRVFNLSRYLADVIKKREN-FKLIM--EPQCTNVCFYYYPPSIKKMNDGPEKNAKLHSIAPII

*T.diomedea* 453 KARMMEAGTLMVQYQPLCDMPNFFRVAISNPTLTTADLDFMADEIDTLGQDIPTPEDW--

*M.leonina* 502 KARMMEAGTLLVQYQPLCDMPNFFRVAISNPALTAADLDYMVEEIDALGKDIPTPHDW--

*L.stagnalis* 516 KAKMMEAGSLMVQYQPLGDMPNLFRVAVSNPILTTRDFDFLLEEIDQLGKDIPLPVDWND

*D.melanogaster* 459 KGRMMQKGTLMVGYQPDDRRPNFFRSIISSAAVNEADVDFMLDEIHRLGDDL--------

*C.elegans* 456 KAGMMQRGTTMVGYQPDKQRPNFFRMIISNQAITREDLDFLIKEIVDIGESLE-------

*H.sapiens* 543 KALMMESGTTMVGYQPQGDKANFFRMVISNPAATQSDIDFLIEEIERLGQDL--------

*H.vulgaris* 478 KSRMTLEGTMLCGYQPLKEHVNFWRMTVINPAVTYDDMDFVVNEIERLGRDL--------

**Figure S11. MUSCLE protein alignment of glutamate decarboxylase homologues from *Tritonia diomedea*, *Melibe leonina*, *Aplysia californica*, *Lymnaea stagnalis*, *Drosophila melanogaster*, *Caenorhabditis elegans*, *Homo sapiens* and *Hydra vulgaris*.**
