# Supplementary material for: Deep mRNA Sequencing of the Tritonia diomedea Brain Transcriptome Provides Access to Gene Homologues for Neuronal Excitability, Synaptic Transmission and Peptidergic Signalling
Source: PLoS One. 2015 Feb 26;10(2):e0118321. doi: 10.1371/journal.pone.0118321 (PMC4342343; doi:10.1371/journal.pone.0118321)
Supplement: S12 Fig — (DOCX) [file pone.0118321.s013.docx]

*T.diomedea* 1 ----------------------------------GRPPLWWFRTNYHDDEKIGFAKHMVQDTDYECHAMKNGHFSSK---PMENGLHENGLHH---S------G--HSDS

*M.leonina* 1 ---------------------MSWRERLAFFSANGRLPLWWFRTNSHDEEKIGFAKHMAQEREPEFHAMKNGHFSRSKM-PVENGYHQESGHQSPAS------G--GGDG

*L.stagnalis* 1 MQGGSTKYTDCPKAFPSHKRNMSWRERLAYLALTAKLT---WRSRGNDEEKLGFAKCPSQDQGFESHPMKNGKSCHS---TNEAEMSEDYLKASLDN------G--W---

*D.melanogaster* 1 ------------------------MSFIAKLKATPLPPLRNILNVAVQTARQQIPERKDYEQPPGSTAQQHHHSQQAQHKAMEAGMDGGDTTEMSSNPFRNAGS--WTND

*C.elegans* 1 -------------------------MASNRFQNLQNWTNKHVFSNSLDYWNQELNEVPSYQNQPQTGESGSNPPPHDRLEPIQESVVSEQPQKDDIN-------------

*H.sapiens* 1 ---------MATLLRSKLSNVATSVSNKSQAKMSGMFARMGFQA-ATDEEAVGFAHCDDLD-------FEHRQGLQMDILKAEGEPCGDEGAEAPVE------GDIHYQR

*N.vectensis* 1 -------------------------MTSDYASLSEAWGRRG---NFLDPFRRMSTEINSL--------LRKKVFTAYSFLSDESENEEDEESEKSGN-------------

*T.diomedea* 63 G----TSRSSLQDDF-----------------------------FLEEAELEGDPRDKITEWQAGWNVTNAIQGMFIVSFPYTVLQGGYWAIVAMVAVAYICCHTGNILV

*M.leonina* 81 GGGGDSDVSSLHSDF-----------------------------FLEEAELDGDPRDKITEWQAGWNVTNAIQGMFIVSFPYTVLQGGYWAIVAMILVAYICCHTGNILV

*L.stagnalis* 94 --------SQLQDDI-----------------------------FVEEAEVGGDPRDKITEWQAGWNVTNAIQGMFIVSFPYTVLQGGYWAVIAMVLVAYICCHTGNILV

*D.melanogaster* 85 GEGGGDGDGEYRNEYQSTSFNEYDGRYQQTDGFRQGSIASEGSSFVCEGEGGGGC--KIDEFQAAWNVTNAIQGMFIVSLPFAVLHGGYWAIVAMVGIAHICCYTGKVLV

*C.elegans* 73 ----------KQEEA--------------------------------KDDGHGEASEPISALQAAWNVTNAIQGMFIVGLPIAVKVGGWWSIGAMVGVAYVCYWTGVLLI

*H.sapiens* 88 GSGAPLPPSGSKDQV------------------------------GGGGEFGGHDKPKITAWEAGWNVTNAIQGMFVLGLPYAILHGGYLGLFLIIFAAVVCCYTGKILI

*N.vectensis* 62 -----LAFGEVEPE---------------------------------EDEG------KTSNLQTFWNIFNANQGVAILAMPYVIKNGGYASIVSIIATAFISNFTNKRLV

*T.diomedea* 140 DCLYDLDP-MGHRVRVRSSYVDIAAAVWGPKY---GARIVHCAQLIELLMTCILYVLLCGDLIQGSFPNTPLSLTSWILVCTTPLLACAFLTSLRHVSTLSMWCTIAHML

*M.leonina* 162 DCLYDLDP-MGHRVRVRSSYVDIAAAVWGQKY---GARIVHLAQLIELLMTCILYVLLCGDLIVGSFPNTPLSLTSWIIVCTTPLLACAFLTSLRHVSTLSMWCTVAHML

*L.stagnalis* 167 DCLYDLDP-MGHRVRVRSSYVDIATAVWGARY---GARIVHCAQLIELLMTCILYVLLCGDLIQGSFPDTPFSLTSWILVCTTPLLACAFLTSLRRVSTLSMWCTVAHML

*D.melanogaster* 193 QCLYEPDPATGQMVRVRDSYVAIAKVCFGPKL---GARAVSIAQLIELLMTCILYVVVCGDLLAGTYPQGSFDSRSWMLFVGIFLLPMGFLKSLKMVSTLSFWCTMSHIV

*C.elegans* 141 ECLYE----NG--VKKRKTYREIADF-YKPGF----GKWVLAAQLTELLSTCIIYLVLAADLLQSCFP--SVDKAGWMMITSASLLTCSFLDDLQIVSRLSFFNAISHLI

*H.sapiens* 168 ACLYEENE-DGEVVRVRDSYVAIANACCAPRFPTLGGRVVNVAQIIELVMTCILYVVVSGNLMYNSFPGLPVSQKSWSIIATAVLLPCAFLKNLKAVSKFSLLCTLAHFV

*N.vectensis* 128 QCLYEQAS-DGSIYRARNSYVEIGEA-FSPRF---GHHLVNAAQIFEQVSYCTLLLILCGSILQSTFPDTPLSQSDWTALAAIMLLPNVLLKSLADVSWVSFLTVLIGEV

*T.diomedea* 246 INAIILIYCFTKAQHWHWADVRLRIDIWTFPISLGIIVFSYTSQIFLPSLECKLRDRSKFKCMMTWTHVTAALFKAIFSYVGFLTWGSGTLEVITNNLQSTSLKLVVNLI

*M.leonina* 268 VNAIIFIYCFTQAGSWHWSSVRLRIDIWTFPISLGIIVFSYTSQIFLPSLEGKLRDRGRFRCMMTWTHIAAAVFKAAFSYIGFLTWGLGTLEVITNNLQSDGLKLVVNLI

*L.stagnalis* 273 INAIILIYCFTKAGQWRWKDVQIRIDIWTFPISLGIIVFSYTSQIFLPSLEGKLRDRSKFRCMMTWTHLAAAVFKALFSYVGFLTWGWDTLEVVTNNLPSTSLKLIVNLI

*D.melanogaster* 300 INAVILGYCLLQIGDWGWSKVRFSIDMENFPISLGVIVFSYTSQIFLPTLEGNMIDRSKFNWMLDWSHIAAAVFKAGFGYICFLTFQNDTQQVITNNLHSQGFKGMVNFF

*C.elegans* 238 VNLIMVLYCLSFVSQWSFSTITFSLNINTLPTIVGMVVFGYTSHIFLPNLEGNMKNPAQFNVMLKWSHIAAAVFKVVFGMLGFLTFGELTQEEISNSLPNQSFKILVNLI

*H.sapiens* 277 INILVIAYCLSRARDWAWEKVKFYIDVKKFPISIGIIVFSYTSQIFLPSLEGNMQQPSEFHCMMNWTHIAACVLKGLFALVAYLTWADETKEVITDNLP-GSIRAVVNIF

*N.vectensis* 233 IYITVFLYSCYHHERWDVASLP-PFAIKKFGAAVGIVVVSYSSQPYMPAIEGSMQRPHNFNNVMNVTYIAVTFVKVFFGFIGYLTFTRDTDQVITNNLPEGVLHMVVNLL

*T.diomedea* 356 LVCKALLSYPLPYFASVELLQTAFFKGASKSLFPSCMDDSRHLKWW-SLALRLGLVLLTCAMAITVPHFALLMGLIGSFTGTMLSFVWPCYFHIRLRWHVMSRLTRIFDI

*M.leonina* 378 LVCKALLSYPLPYFASVELLETAFFKGSGKTLFPSCLDGSMHLKWW-GLALRLGLVLLTCAMAVTVPHFALLMGLIGSFTGTMLSFVWPCYFHIRLRWHLMSRAARALDV

*L.stagnalis* 383 LVCKALLSYPLPYYASVELLETAFFKGKPATCFPPCLDETRRLKWW-GLSLRMCLVLFTCAMAIIVPHFALLMGLIGSFTGTMLSFVWPCYFHLRLRWYVMARTTRVLNI

*D.melanogaster* 410 LVIKALLSYPLPYYAACELLERNFFRGPPKTKFPTIWNLDGELKVW-GLGFRVGVIVSTILMAIFIPHFSILMGFIGSFTGTMLSFIWPCYFHIKIKGHLLDQKEIAKDY

*C.elegans* 348 LVVKALLSYPLPFYAAVQLLKNNLFLGYPQTPFTSCYSPDKSLREW-AVTLRIILVLFTLFVALSVPYLVELMGLVGNITGTMLSFIWPALFHLYIKEKTLNNFEKRFDQ

*H.sapiens* 386 LVAKALLSYPLPFFAAVEVLEKSLFQEGSRAFFPACYSGDGRLKSW-GLTLRCALVVFTLLMAIYVPHFALLMGLTGSLTGAGLCFLLPSLFHLRLLWRKLLWHQVFFDV

*N.vectensis* 342 VLFLAATSYTIPVYTVFDILENISFP-CGRMEHPSSAKGKDKLSYFQALTARLCVISFTLLVGVLVPHFGLYMALVGSFTGMCLAFIFPAFFHMKICYQRMQWYGFFIDS

*T.diomedea* 465 VIIILGALCGSIGIYYSAHALSRAFR-GLPPEPTHTLKMPIKN

*M.leonina* 487 VIIILGALCGAVGIYYSAHALLRAFR-GLPPEPTHTLKMPIKN

*L.stagnalis* 492 FIIVLGLACGGIGIYYSAHALTRAFQ-GLPPSS----------

*D.melanogaster* 519 LIIGLGVLFGVIGIYDSGNALINAFEIGLPF------------

*C.elegans* 457 GIIIMGCSVCISGVYFSSMELLRAIN-SADS------------

*H.sapiens* 495 AIFVIGGICSVSGFVHSLEGLIEAYRTNAED------------

*N.vectensis* 451 FVAIFGIVGAIIGCHFSVLALITTYQ-KHEL------------

**Figure S12. MUSCLE protein alignment of vesicular GABA transporter homologues from *Tritonia diomedea*, *Melibe leonina*, *Lymnaea stagnalis*, *Drosophila melanogaster*, *Caenorhabditis elegans*, *Homo sapiens* and *Nematostella vectensis*.**
