# Supplementary material for: Deep mRNA Sequencing of the Tritonia diomedea Brain Transcriptome Provides Access to Gene Homologues for Neuronal Excitability, Synaptic Transmission and Peptidergic Signalling
Source: PLoS One. 2015 Feb 26;10(2):e0118321. doi: 10.1371/journal.pone.0118321 (PMC4342343; doi:10.1371/journal.pone.0118321)
Supplement: S13 Fig — (DOCX) [file pone.0118321.s014.docx]

*T.diomedea* 1 -MSSTSGKPRA-----EKEYIEM-G--------QSCHV-TTDPQGNGR-SPEVKTGDGTIEREHWTGKIEFVLACVGQCIGFGNVWRFPYLCYKNGGGAFLIPFFLTAIF

*M.leonina* 1 MSSSTSGKGRD-----EKEYIEMGG--------QSCHVATSDPQGNGRTTAEVKAEDGPPEREHWTGKIEFVLACVGQCIGFGNVWRFPYLCYKNGGGAFLIPFFMTAIF

*L.stagnalis* 1 ---MTMSTAKH-----NSEYIEM-G--------QGPAP-HQEASNNGR-AIEAKLVE-TPERELWTGKVEFVLACVGQCIGFGNVWRFPYLCYKNGGGAFLIPFVATIVF

*D.melanogaster* 1 MYTNSASDGDGGGDVCKHESIEMSKELGHTTSLQSSSPTTTDTSNKQQ----LVKIEQLPDRGSWSSKMDFILSVVGLAIGLGNVWRFPYLCYKNGGGAFILPYIITLFL

*C.elegans* 1 ----------------------------------------------------MVEATSVAEREQWSSWADFIMSCIGYAIGLGNVWRFPYLCYQNGGGAFLIPYCISLVF

*H.sapiens* 1 MATNGSKVADG------QISTEV-----------SEAPVANDKPKTLVVKVQKKAAD-LPDRDTWKGRFDFLMSCVGYAIGLGNVWRFPYLCGKNGGGAFLIPYFLTLIF

*N.vectensis* 1 ---MRILNEKY-----AHYFLQ------------ECSLLSAKNETQFC----REKGFEVVERETWVGKLDFILALIGFSVGLGNIWRFPYLCYKNGGGCFLIPYLICLLL

*T.diomedea* 94 AGIPMYFMELALGQWLSVGGLGIWKIAPIWKGVGYAAAIMAFWLNTYYIVILAWTVFYLFHSFA-STVPWASCGNSWNTPRCRSAYETDSLPFNCTNGTYSFEVNVTKSH

*M.leonina* 98 AGIPMYFMELALGQWLSVGGLGIWKIAPIWKGVGYASAIMAFWLNTYYIVILSWTMFYLIHSLA-ATVPWATCGNDWNTPRCLSAYATDTLPFNCSNATLSFEVNVTKSL

*L.stagnalis* 91 AGIPMYFMELALGQWLSVGGIGIWKITPIFKGVGYASAIMAFWLNTYYIVILAWTLFYLFHSFT-SVLPWGRCGQDWNTNKCMSAYERYKIPFNCTNGTSWFEVNVTSEY

*D.melanogaster* 107 AGIPMFFMELALGQMLTIGGLGVFKIAPIFKGIGYAAAVMSCWMNVYYIVILAWAVFYFFMSMR-ADVPWRTCNNWWNTVNCVSQYER-------KNLHCWDKIINGTTQ

*C.elegans* 59 CGAPLFILETSWGQLMSVGGLGMFKICPIFKGVGIAAAVMAFWLNIYYIVVLSWAATYLYNSFTMSDVPWKNCDHAWNTPNCRSEYVK----IPCDSNRTIAEFFNVKVL

*H.sapiens* 93 AGVPLFLLECSLGQYTSIGGLGVWKLAPMFKGVGLAAAVLSFWLNIYYIVIISWAIYYLYNSFT-TTLPWKQCDNPWNTDRCFSNYSM----------------------

*N.vectensis* 87 AGVPLLILEVCLGQFMSQGGITAWKICPLFQGIGYASVVIVQYLNIYYIVILGWAFYYMFHSFQ-AVLPWSHCDNDWNTEKC-------------HVGRKMITTFVNATY

*T.diomedea* 203 NSSVIPGLYPEFNCSQDYD-------ESKYTAPVKEYWLRKALKITESIEDPGVLQWQLALCLLLVWVMCYFCIWKGVKGTGKVVYVTAIFPYILLFILLIRGVTLPGAS

*M.leonina* 207 NASVIPTLYPDFNCTQDYD-------ESKYTNPVKEYWLRKALQISKGIDDPGALQWQLALCLLLVWVMCYFCIWKGVKGTGKVVYVTAIFPYILLSVLLVRGITLPGAM

*L.stagnalis* 200 NSSQIPSIYPDYNCSQDYD-------AYRFVSPVKEFWLKKALKLTDGIGDAGQLQWELSLCLLGVWVLCYFCIWKGVKWTGKVVYVTALFPYILLTILLIRGVTLPGAA

*D.melanogaster* 209 KVCSVSAL--------NIT-------SLELTGPVKEFWERRALQISHGIEEIGNIRWELAGTLLLVWILCYFCIWKGVKWTGKVVYFTALFPYVLLTILLVRGITLPGAL

*C.elegans* 165 THDHIHEYKKQFFVGEKMNWTVCSAADLSVVSPVKEFWNHRVLGISSGLENPGGIRWDLALFLLLVWIICYLCIFKGVKWTGKVVYITASFPYMMLFCLLIRGLTLEGAG

*H.sapiens* 180 -----------------VN-------TTNMTSAVVEFWERNMHQMTDGLDKPGQIRWPLAITLAIAWILVYFCIWKGVGWTGKVVYFSATYPYIMLIILFFRGVTLPGAK

*N.vectensis* 183 NATTNATITNATTV--TVE-------ANGTVSPIVEFWERKVLNISGGLDQIGTINWQMALCLLFAWIVCYLCVFKGVKSTGRVVYFTATFPYVLLTIICVRAVTLPGAR

*T.diomedea* 306 DGIRFYITPRLEELGKSTVWVDAASQILFSYGIGLGTGVALGSYNKYHNNVYKDAVLISCLNSSTSVFAGFVTFSIIGFMAHEQKRSIELVAATGPGLAFLAYPSAVTQL

*M.leonina* 310 DGVRFYITPRLEELGNSTVWVDAASQILFSYGIGLGTGVALGSYNKYHNNVYKDAVLISCLNSTTSVFAGFVTFSIIGFMAHEQKRSIKLVAASGPGLAFLAYPSAVTQL

*L.stagnalis* 303 DGIRFYITPNMKKLAESTVWVDAASQILFSYGVGLGTCTALGSYNKYHNNVYKDAVLISCLNSSTSVFAGFVIFSVIGFMAHEQKRSVHLVAESGPGLAFLVYPSAVTQL

*D.melanogaster* 304 EGIKFYIIPNFSKLTNSEVWIDAVTQIFFSYGLGLGTLVALGSYNKFTNNVYKDALIVCTVNSSTSMFAGFVIFSVIGFMAHEQQRPVADVAASGPGLAFLVYPSAVLQL

*C.elegans* 275 VGLEFYLKPDFSKLLESKVWVDAVTQVFFSYGLGLGALVALGSYNKFNNNVYKQALTVCFVNSGTSVFAGFVIFSFIGFMATQQEKSVAEVAQAGPGLLFLAYPSGILQL

*H.sapiens* 266 EGILFYITPNFRKLSDSEVWLDAATQIFFSYGLGLGSLIALGSYNSFHNNVYRDSIIVCCINSCTSMFAGFVIFSIVGFMAHVTKRSIADVAASGPGLAFLAYPEAVTQL

*N.vectensis* 284 EGIRFYLEPDWSRLQDGSVWLQAATQVFFSYSIGLGTLIALGSYNKFKNNCYRDCIIFACVNSGTSFYGGFVIFSVLGFMAEKQGVPIQEVAESGPGLAFIAYPAAVAEM

*T.diomedea* 416 PISPLWAIMFFLMLLMLGMDSQFCTMEGFFTALIDEFPNTLRRH--REIFIACVCFVSYLIGLSMVTEGGMYVFQIFDFYSASGIAVLLLIFFECIAISWSYGINRFYDN

*M.leonina* 420 PISPLWAILFFVMLLMLGMDSQFCTMEGFFTALIDEFPNTLRRH--RELFIAGVCFVSYLIGLSMVTEGGMYVFQIFDFYSASGIAVLLLIFFECIAISWSYGVNRFYDN

*L.stagnalis* 413 PISPLWAILFFLMLLTLGMDSQFCTMEGFFTALIDEFPRHLRRH--REIFIALVCLASYLIGLSMVTEGGMYVFQLFDFYSASGITVLLLIFFECIAISWSYGVNRFYED

*D.melanogaster* 414 PGSPMWSCLFFFMLLLIGLDSQFCTMEGFITAIIDEWPQLLRKR--KEIFIAIVCALSYLVGLTCITQGGMYIFQILDSYAVSGFCLLWLIFFECVSISWCYGVDRFYDG

*C.elegans* 385 PYTQFWSCLFFLMVLFLGVDSQFCTMEGFFTAIIDEFPQIRRKKYGREIFVGVICVISYLIGLTTVTEGGFYVFQLFDFYAASGWALLWLLFFECIAISWSLGIDRWYEH

*H.sapiens* 376 PISPLWAILFFSMLLMLGIDSQFCTVEGFITALVDEYPRLLRNR--RELFIAAVCIISYLIGLSNITQGGIYVFKLFDYYSASGMSLLFLVFFECVSISWFYGVNRFYDN

*N.vectensis* 394 PISPLWSILFFFMVILLGLDSEFVGVEGFVTAIVDMFPGYLRRGYRKEIFIAACSTFWFFIGLVMVTEGGMYVFQLFDNYSASGICLLWISLFESIGIGWVYGAERFYQN

*T.diomedea* 524 LRDMFGFYPNVFWKFCWTISTPAITLGVVLFSLSQFEPMTY-VGYTF--PPWAHAVGGVLGMSSISCIPLYMIFKFAATPGSLKHRVKVLFRPQTDMDTM--TRPPPYSA

*M.leonina* 528 LRDMFGFYPNVFWKFCWTVSTPAITLGVVLFSITQFEPVTY-VGYKF--PRWAHAVGGVLGMSSISCIPLYMVFKFSTTPGTIRHRVKVLFRPHTDVDTV--IRPPPYSA

*L.stagnalis* 521 LKDMFGFYPCFFWKICWCVTTPAICLGVVLFSVFTFQPVTY-VGYQF--PTWAHAVGGVVGASSIICIPVYMVYKFLITSGSLSHRVKILFRPDLNFRRGS-SDPPPYSA

*D.melanogaster* 522 IKDMIGYYPTVWWKFCWCVTTPAICLGVFFFNIVQWTPVKY-LDYSY--PWWAHAFGWFTALSSMLYIPLYMFWLWKRTPGELSEKIRALVRIDEDVTRL--REKMLREA

*C.elegans* 495 MKSMIGYYPSAWWKFCWVFATPSVCFGVLLFGLIKYQPLRI-DAYNYDYPVWGHIFGWFLSLSSMLCIPGYAIWIWFKTPGTVQEKIKLLCRPDIEIKGA--MENAENLE

*H.sapiens* 484 IQEMVGSRPCIWWKLCWSFFTPIIVAGVFIFSAVQMTPLTM-GNYVF--PKWGQGVGWLMALSSMVLIPGYMAYMFLALKGSLKQRIQVMVQPSEDTVRP--ENGPEHAQ

*N.vectensis* 504 MTDMIGFRINPYLKVCWIFCTPIFCLAVFIFSLVNYEPLTYNLTYKY--PVWGDAIGWLLALSSIVCIPLLAVINLMRAEGDFIDRLRSVTTPCLPPGRGCYTKQETDAM

*T.diomedea* 629 IPRTVGDGAITL-------

*M.leonina* 633 IPRTVGEGAITL-------

*L.stagnalis* 627 IPRTVGGGVVRL-------

*D.melanogaster* 627 YAKEIEFNSL---------

*C.elegans* 602 LVEDFQNAI----------

*H.sapiens* 589 AGSSTSKEAYI--------

*N.vectensis* 612 NGKAMGNGREHNFEVDTKM

**Figure S13. MUSCLE protein alignment of membrane GABA transporter homologues from *Tritonia diomedea*, *Melibe leonina*, *Lymnaea stagnalis*, *Drosophila melanogaster*, *Caenorhabditis elegans*, *Homo sapiens* and *Nematostella vectensis*.**
