# Supplementary material for: Deep mRNA Sequencing of the Tritonia diomedea Brain Transcriptome Provides Access to Gene Homologues for Neuronal Excitability, Synaptic Transmission and Peptidergic Signalling
Source: PLoS One. 2015 Feb 26;10(2):e0118321. doi: 10.1371/journal.pone.0118321 (PMC4342343; doi:10.1371/journal.pone.0118321)
Supplement: S14 Fig — (DOCX) [file pone.0118321.s015.docx]

*T.diomedea* 1 ---MVSKMDS---LHKRVFNSALVVLILASH----------IHPVFMLKDKPLTIGGIFPM-S--GSWAGGVSCLPAVEMALEDVNNRTDILPDYKLEMRFGDSECKPGL

*M.leonina* 1 ---MVYLKRC---STSQVL---LVLFILTLN----------IRPVQVLKEKSLTIGGIFPM-S--GSWAGGVSCRPAVEMALEDVNNRTDILPDYKLEMQVGDSECKPGL

*A.californica* 1 -----MLQDV---VRYSFL---KSNYLWTYN----------IQLTVSFKKKPITF------------------------------------------------LQCKPGL

*L.stagnalis* 1 -----MFGSK---LTSLLI---LLFYIRVQN------------GQTLYKQKTLTIGGIFPM-S--GSWAGGVGCLPAVKMALDDVNNRTDILPDYKLEMQSDDSQCKPGL

*D.melanogaster* 1 MRKDMTSDGA---VTFWIF---LLCLIASPH----------LQGGVAGRPDELHIGGIFPI-AGKGGWQGGQACMPATRLALDDVNKQPNLLPGFKLILHSNDSECEPGL

*C.elegans* 1 -----MFVRS----SWLLL---WGTIVWASA-----------------EPVTLHIGGTFPMESGSGGWAGGEACLPAVEMALKDVNSRLDILPGYVLNMTNHNSQCQPGL

*H.sapiens Β1b* 1 MGPGAPFARVGWPLPLLVVMAAGVAPVWASHSPHLPRPHSRVPPHPSSERRAVYIGALFPM-S--GGWPGGQACQPAVEMALEDVNSRRDILPDYELKLIHHDSKCDPGQ

*N.vectensis* 1 ------MRRMNWHKPSLLI---VCILILTNA--------------GFTAKKELYIGGFFPL-SGVPVASSGRDILPACKMALEMLNNRSDILPDYRLNLLARDTKCDVGH

*T.diomedea* 92 GTKVLYKLLY-EKPTKLLVLTGCSIVSTFVAQAAKMWKLVVLSYGGSSPALSNRERFPTLFRTHPSATLHNPIRVKVFKKFNWNRISTIQEIQELFTSTIDDLEKRVKEA

*M.leonina* 89 GAKVLYNLLY-EKPTKLLMLTGCSIVSTFVAQAAKMWKLVVLSYGASSPALSNRERFPTLFRTHPSATLHNPIRVKVFKKFNWNRISTIQEIRELFTSTIDDLEKRVKDN

*A.californica* 42 GTKVLYKLLY-EKPTKLLVLTGCSIVSTFVAQAAKMWKLVVLSYGGSSPALSNRERFPTFFRTHPSATLHNPIRVKVFEKFKWTRISTIQETQELFTSTIEDLEQRVKEA

*L.stagnalis* 85 GTKVLYKLLY-DKPTKLLVLTGCSIVSTFVAQAAKMWKLVVLSYGGSSPALSNRERFPTFFRTHPSATLHNPIRVKVFKKFNWHRISTIQETQELFTSTIEDLEQRVKEA

*D.melanogaster* 94 GASVMYNLLY-NKPQKLMLLAGCSTVCTTVAEAAKMWNLIVLCYGASSPALSDRKRFPTLFRTHPSATVHNPTRIKLMKKFGWSRVAILQQAEEVFISTVEDLENRCMEA

*C.elegans* 82 AMQQLYDFLY-KPPTKLMLLTGCSPVTTVIAEAAPVWKLVVLSYGGSSPALSNRNRFPTLFRTHPSANMQNPTRIHIMEKFKWKRFTILMSVEEVFVTTAKDLEAIARKK

*H.sapiens Β1b* 108 ATKYLYELLY-NDPIKIILMPGCSSVSTLVAEAARMWNLIVLSYGSSSPALSNRQRFPTFFRTHPSATLHNPTRVKLFEKWGWKKIATIQQTTEVFTSTLDDLEERVKEA

*N.vectensis* 87 GIKVLYEYLSRSTPVITLLGPACSAVTKVVAEVAWHWSLLQVSYASTASDLSNKEKYPLLYRTVQPDSAFNSARVSVLEFYGWRRVGTLRQDDFVFSSVMSELHQSLEDH

*T.diomedea* 201 GINIAVRQSFLTDPLNAVRNLKRQDARIIVGVFYEDMARRVFCQAYKERLFGKKYVWFIIGWYPDN-WYRK--KDDRHNCTVEQLEEALEGHFTTEAVVLHQENTMTDVG

*M.leonina* 198 GIDIAVRQSFLTDPMNAVRNLKRQDARIIVGVFYENMARQVFCQAYKEKLYGKKYVWFIIGWYPDN-WYKK--KDDHHNCTVEQLEEALEGHFTTEAVILHQEPTMTDVG

*A.californica* 151 NIDIAVRQSFLTDPTNAVRNLKRQDARIIVGVFYEDMARRVFCQAYKERLYGKKYVWFIIGWYPDN-WYKM--KDDRHNCTVEQLEEALEGHFTTEAMMIHQEPTMTDVG

*L.stagnalis* 194 GIDIAVRQSFLTDPTNAVRNLKRQDARIIVGVFYEDMARRVFCQAYKERLFGKKYVWFIIGWYPDN-WYKV--KDDRHNCTVEQLEEALEGHFTTEAVMIHQEATMTDVG

*D.melanogaster* 203 GVEIVTRQSFLSDPTDAVRNLRRQDARIIVGLFYVVAARRVLCEMYKQQLYGRAHVWFFIGWYEDN-WYEVNLKAEGITCTVEQMRIAAEGHLTTEALMWNQNNQTTISG

*C.elegans* 191 GIKVD-RQSFYGDPTDAMKTLQRQDARIIVGLFYVTEARKVLCQAYHHGLYGRRYVWFFIGWYADT-WYIPP-PEEHLNCTAEQMTEAAEYHFTTESVMLSRDNIPAISE

*H.sapiens Β1b* 217 GIEITFRQSFFSDPAVPVKNLKRQDARIIVGLFYETEARKVFCEVYKERLFGKKYVWFLIGWYADN-WFKI--YDPSINCTVDEMTEAVEGHITTEIVMLNPANTRSISN

*N.vectensis* 197 NISVVSPETFSKEPRRHLEAIKDQDARIIIGMFYEDAARRVFCEAYKLGMYGSQYVWILLDWADNHQWWLT--PDDDVDCTADQMASAVTGYFSIDSVNLERTDKPGISG

*T.diomedea* 308 MTASRFTERLDAIL-----NVSDTSLMNGYPEAPLAYDAVWALAFAFNKTINRLAEKGMKLEEFDYYNEEIHNAIYSAMNSTKFLGISGNVAFSSKGERIAWTQIEQFIN

*M.leonina* 305 MTAQHFTERLDTML-----NVSDTSLMNGYPEAPLAYDAVWALAFAFNKTINKLSEKGLKLEDFDYYNEEIHSAIYTAMNSTRFLGISGNVAFSSKGERIAWTQIEQFIN

*A.californica* 258 MTAQNFTSRLNEIL-----NTSDTSLITGYPEAPLAYDAVWAVALAFNKTAAKLAERNMKLEDFDYYNEEITDAIYSAMNSTKFLGISGNVAFSSKGDRIAWTQIEQFIN

*L.stagnalis* 301 MTAQRFTQRLNKIL-----NTTDTSLITGYPEAPLAYDAVWAVAHAFNKTATQLAERGMKLEDFDYYNEDITNAIYSAMNSTKFLGISGNVAFSAKGDRIAWTQIEQFIN

*D.melanogaster* 312 MTAEEFRHRLNQALIEEGYDINHDRYPEGYQEAPLAYDAVWSVALAFNKTMERLTTGKKSLRDFTYTDKEIADEIYAAMNSTQFLGVSGVVAFSSQGDRIALTQIEQMID

*C.elegans* 298 MTGMQFQQRLTQYF------QKDTANVGGFPEAPLAYDAVWALALAFNCTRNNLPSH-IRLENFTYDNKVIADTLFQCVKNTSFRGVSGKVMFSDSGDRIARTQIEQMQG

*H.sapiens Β1b* 324 MTSQEFVEKLTKRL------KRHPEETGGFQEAPLAYDAIWALALALNKTSGGGGRSGVRLEDFNYNNQTITDQIYRAMNSSSFEGVSGHVVFDASGSRMAWTLIEQLQG

*N.vectensis* 305 LTSKEFLKEFSRYH-----PNGTANLYV-----PYAFDSMWMIALALNNTAGQLHRQNKSLDQFCYGDHEMARLLQRSTESLVFRGVTGMIEFSKKGERKQPVWINQLQE

*T.diomedea* 413 GSYVKLGVYDTIADNLTWYNK-EKWAGGR-PPNDH-TRIIDHLRVVSHSLYFSMCGLAGLGMLAGLFCLAFTYVNRDRQCIAFSQPLINCLTVVGCIICLGCIFLLGL--

*M.leonina* 410 GSYVKLGVYDTIADNLTWYNK-EKWAGGR-PPNDH-TRIIDHLRVVSHSLYFSMCGLAALGMMAGVFCLVFTYLHRHRQCIALSQPLINSLTVVGCILCLGCIFLLGL--

*A.californica* 363 GSYVKLGIYDAVADSLTWYNK-EKWLGGR-PPPDH-TRVIDHLRVVSRTLYFSMCGLAGVGILAGLFCLVFNWLHFNRQCVAFSQPAINNLTVLGCIICLGCIVLLGL--

*L.stagnalis* 406 GSYVKLGVYDAVADNLTWYRK-EKWLGGR-PPPDH-TQVIDDLRVVSHTLYFSMCGLAGLGILAGILCLIFNHINRNRQCVAFSQPAINNLTVVGCMICLGCIVLLGL--

*D.melanogaster* 422 GKYEKLGYYDTQLDNLSWLNT-EQWIGGK-VPQDR-TIVTHVLRTVSLPLFVCMCTISSCGIFVAFALIIFNIWNKHRRVIQSSHPVCNTIMLFGVIICLISVILLGI--

*C.elegans* 401 GKYKIMGYYDTTSGDLEWYNK-EQWLNGKGPPPDS-TVIKKHAMTVSNEFYYPTILFAVLGIAACVFIYLFTQKHHERLIIFQSQPECNNILLIGCSLCLFSLFLIGLPS

*H.sapiens Β1b* 428 GSYKKIGYYDSTKDDLSWSKT-DKWIGGS-PPADQ-TLVIKTFRFLSQKLFISVSVLSSLGIVLAVVCLSFNIYNSHVRYIQNSQPNLNNLTAVGCSLALAAVFPLGL--

*N.vectensis* 405 WRPVHVGLYDPANNKIMQNTTLPIWQGDG-PPSDGLVHYLSRVEHVTMSLLWVTIVLGMIGILLASGFLCFNVRYRNHSYIKLSSPNLNNVIIVGAILIYMSIILVAM—

*T.diomedea* 518 DGKFVAEDVYPKVCQARAWLLSLGFTLSFGSMFSKIWTVHQL-------TTSR--KKE-R-GVQTWELFTVLGILVLLDVAILSAWQVMDPLQRRLEFFAREQPTDTEDD

*M.leonina* 515 DGKYVSENVYPLVCQVRTWLLSLGFTLSFGSMFSKIWTVHQL-------TTSR--KKD-R-GVHTWELFVVLGILIVLDVCVLSAWQVMDPLQRRLEFFAREQPTDTEDD

*A.californica* 468 DGKFVSEQTYPVICQARAWLLSLGFTLSYGSMFSKIWTVHQM-------TTTR--KKE-RTGVQIWELFTVLAILMLLDVGVLTAWQVLDPLQRQLETFARVQPTNTEED

*L.stagnalis* 511 DGKFVPDYRYPLVCQIRAWLLSLGFTLSYGSMFSKIWTVHQM-------TTTR--KKD-RTGVQIWELFTVLAILVLLDVGVLTAWQVLDPLQRKLETFARIQPTNTEDD

*D.melanogaster* 527 DGRFVSPEEYPKICQARAWLLSTGFTLAYGAMFSKVWRVHRF-------TTKA--KTDPKKKVEPWKLYTMVSGLLSIDLVILLSWQIFDPLQRYLETFPLEDPVSTTDD

*C.elegans* 509 DDISISESLFPLLCHARVTILLFGFTFAYGSMFAKVWIVHRMGATENQQLASR--QKD--EPISSSKFYVIVAALTAVDVFVCFVWVLIDPLHLTEQKFPLFTPADSEED

*H.sapiens Β1b* 533 DGYHIGRNQFPFVCQARLWLLGLGFSLGYGSMFTKIWWVHTV-------FTKKEEKKEWRKTLEPWKLYATVGLLVGMDVLTLAIWQIVDPLHRTIETFAKEEPKE-DID

*N.vectensis* 512 DGRTVSPASLTHICTATSYLLGIGYSLAVGAMFSKTWRVHQI-------FNKV--KPK-KKVFQDSELIGLVVLMIAVDLVVFSLWAGVDPLTYRLQ---QSGEAIREGD

*T.diomedea* 617 IELQPQLEHCHSDNLTVWLGVLFGYKGILLIFGIFLAYETRSVKLKQVNDSRFVGMSIYNVVVLCVITAP-ISLIIGNQEDATFAFVAQAIVLCSFLSMGLIFVPKIKEI

*M.leonina* 614 IELQPQLEHCHSDNLTVWLGVLFGYKGILLIFGIFLAYETRSVKLKQVNDSRFVGMSIYNVVVLCVITAP-SSLIIGNKEDANFAFVAQAIVLCSFLSMGLIFVPKIKEI

*A.californica* 568 IELRPQLEHCHSENLNVWLGVLFGYKGILLIFGIFLAYETRSVKLKQVNDSRFVGMSIYNVVVLCVITAP-ISLIIGNQEDATFAFVSLAIILCSFLSMGLIFVPKIMEI

*L.stagnalis* 611 IELRPQLEHCHSDNLNIWLGVLFGYKGILLIFGIFLAYETRSVKLKQVNDSRFVGMSIYNVVVLCVITAP-ISLIIGNQEDATFAFVSLAIILCSFLSMGLIFVPKVMEI

*D.melanogaster* 628 IKIRPELEHCESQRNSMWLGLVYGFKGLILVFGLFLAYETRSIKVKQINDSRYVGMSIYNVVVLCLITAP-VGMVIASQQDASFAFVALAVIFCCFLSMLLIFVPKVIEV

*C.elegans* 615 EMIMPVLQQCQSNQQEVWIGIIMGFKCLLLVFGTFLSYETRNLKLRFINDSRFVGLAIYNVAVMTLVTAPVVTLLIHGKVDANFAFISLTVLICTYISVGLIYGPKIRHI

*H.sapiens Β1b* 635 VSILPQLEHCSSRKMNTWLGIFYGYKGLLLLLGIFLAYETKSVSTEKINDHRAVGMAIYNVAVLCLITAP-VTMILSSQQDAAFAFASLAIVFSSYITLVVLFVPKMRRL

*N.vectensis* 609 VMVTPFYESCHSKHSTTWLYIIMGYKGLILLVGCFISWETRKVKVRALNDSRFVGMSVYNIVLAVVIGGP-LTLLIGGDRDSFVALYSFFVFFPTSISLCLLFVPKIRKV

*T.diomedea* 726 ----------------------------------------------I-RNPGRDGKEAKGLTDSL-VSR-----------------------------------------

*M.leonina* 723 ----------------------------------------------I-RNPGGDGNESKGLTDSL-ISR-----------------------------------------

*A.californica* 677 ----------------------------------------------I-KHPQRDGQEVKSLTDSL-VSR-----------------------------------------

*L.stagnalis* 720 ----------------------------------------------L-QNPQRDGQEVKSLTDSL-VSR-----------------------------------------

*D.melanogaster* 737 ----------------------------------------------I-RHP-KDKAESKYNPDSA-ISK-----------------------------------------

*C.elegans* 725 ----------------------------------------------IKVPPSADEIQLNGNVGPGVMSK-----------------------------------------

*H.sapiens Β1b* 744 ----------------------------------------------ITRGEWQSEAQDTMKTGSS-TNN-----------------------------------------

*N.vectensis* 718 RKHAEEFLRFQSLSAMRMSSEYRDNSYSSSSTDHVTILRLQTELAQVKRELEHHKATTKTSEDPLPVNRKLSFDQDVRTDHRTTSDENTSRPTRSETCQDLSQFQSTPMG

*T.diomedea* 747 ----------------------------------------------------------------------------EEEERHQKMLAENEQLKKQIADME----------

*M.leonina* 744 ----------------------------------------------------------------------------EEEERHQKMLAENEQLKKQIAEME----------

*A.californica* 698 ----------------------------------------------------------------------------EEEERHQKMLGENEQLKKQIAEME----------

*L.stagnalis* 741 ----------------------------------------------------------------------------EEEERHQKMLGENEQLKKQIAEME----------

*D.melanogaster* 757 ----------------------------------------------------------------------------EDEERYQKLVTENEQLQRLITQKE----------

*C.elegans* 748 ----------------------------------------------------------------------------VDQKRYDMLKKENETLQIQIEEKE----------

*H.sapiens Β1b* 766 ----------------------------------------------------------------------------NEEEKSRLLEKENRELEKIIAEKE----------

*N.vectensis* 828 VQRRCNTFSVPDHNVTGSDDGSATLVNLKLENAGLRRELKESKISEAGKMCKILYQNAELNRKLEELSLKQRAGSTDEEEVVSRLMRENTELKRQLGEVSILNSVWCDVT

*T.diomedea* 771 ---------------DRVKDLNQ-------------------KLQDRSRHRVSIGNK------SHSK--------VVVVDPLDTNINTNG--------------------

*M.leonina* 768 ---------------DRIKDLNQ-------------------KLQDRGRHRVTVGSKQP----PHTK--------VIVVDPLETTPNSHG--------------------

*A.californica* 722 ---------------DRIRELNQ-------------------KLQDRTRQRHSATSTTTTVTSTAPT------------NTVSMIVPAPG--------------------

*L.stagnalis* 765 ---------------DRIRELNQ-------------------KLQERARQRVTSNNMTSRPNNTNNK--------VIIVDHLSSDTCPQN--------------------

*D.melanogaster* 781 ---------------EKIRVLRQ-------------------RLVERGDAKGT---------------------------------------------------------

*C.elegans* 772 ---------------RKIHECKE-------------------RLEELTKNSET--------------------------EDMNAQLLCEN--------------------

*H.sapiens Β1b* 790 ---------------ERVSELRH-------------------QLQSRQQLRSRR--------------------------------------------------------

*N.vectensis* 938 PRLSRKARHDSNTGRDDIKELNKLLTVDLGGAGRSCSFTRLNRLSPIGKSQSSLDQLEPKDSLERPEHEKGEVEVTIMIDDTTSLIDTQGSDSMKEYTKNGHGDGKSGTT

*T.diomedea* 813 ----------------------------KGKNMAINSCKLSPNLNSARNNLSPSLHHRNKPQASSTL-----------------PSPNFP--NPSLLPGYDDSWKSKDSN

*M.leonina* 812 --------------------------------------KHVPNPHPNADKANPS-SPLLSPTSSSTLA----------------PNSSLP---GDKEVGCDGEAHHRDSN

*A.californica* 766 -----------------GVPVGICRPINSGHVLVVDPMITEHPPSDKAKHLIFA-DTADTVTSARQFAAMTSSHQNLTD-----CSPVVA--DANSDSGLASETYGKNSY

*L.stagnalis* 813 ----------------QLSHDSICHPQNQ---ILHDSICH---PQNQILHDSIC-HPQNQMSAESMLAINSSPHKADVMMMMTQSSPVVA--DANSDSGLAEESFGKNSS

*D.melanogaster* 800 -----------------------------------------------------------ELNGATGV-----------------ASAAVA--TTSQPASLINSSAH----

*C.elegans* 802 --------------------------------------------DKQIADENLTYSTATTLTTTIPLIDL--------------QNGNHP-----------GQIYENDND

*H.sapiens Β1b* 810 -------------------------------------------------------HPPTPPEPSGGL-----------------PRGPPE---PPDRLSCDGSRVH----

*N.vectensis* 1048 SNENISASVNGGFIFDEGPSVTSDGKKKVSRTVNDHSNNTDHNDRSDRGDRNIR-SDQDELKNFDELLCEKTRLATQRLLKKPSNNDVTSHGIPPIRRGQIESTASDSGM

*T.diomedea* 876 QN-PTTSVDSLHTNRNVLIQ----------------------------------

*M.leonina* 864 QNQTATVVDGLLTNRKVLTQ----------------------------------

*A.californica* 851 GKKVSYPDSSDIIETMG-------------------------------------

*L.stagnalis* 898 DKKGSSPEAVEIVTLNPLNI----------------------------------

*D.melanogaster* 828 ----ATPAATLAITQGE-------------------------------------

*C.elegans* 843 DDGSSTSSDEILL-----------------------------------------

*H.sapiens Β1b* 841 ----------LLYK----------------------------------------

*N.vectensis* 1157 ESAPVSPQHPFVLAYNERMPSTTEAADQTFSKRRKKSKQDRKTHIEKVEKTFFV

**Figure S14. MUSCLE protein alignment of metabotropic GABA_B_ type Β1 homologues from *Tritonia diomedea*, *Melibe leonina*, *Aplysia californica*, *Lymnaea stagnalis*, *Drosophila melanogaster*, *Caenorhabditis elegans*, *Homo sapiens* (β1b subunit) and *Nematostella vectensis*.**
