# Supplementary material for: Deep mRNA Sequencing of the Tritonia diomedea Brain Transcriptome Provides Access to Gene Homologues for Neuronal Excitability, Synaptic Transmission and Peptidergic Signalling
Source: PLoS One. 2015 Feb 26;10(2):e0118321. doi: 10.1371/journal.pone.0118321 (PMC4342343; doi:10.1371/journal.pone.0118321)
Supplement: S15 Fig — (DOCX) [file pone.0118321.s016.docx]

*T.diomedea* β 1 --------------------------------MERVSVGPCLIFFTFI------------VLVRC---DKSWDNFRNVTE------------------IIESLLNGYDIR

*M.leonina* β 1 MCILPLMKKLTSCKKRLSTFPKTMPKKRTQFNMDYFAAIPFLIAFTVS-----------FTCVSC---NTSAESLANVTE------------------MIETLLEGYDIR

*A.californica* β 1 -----------------------------------------------------------------------------------------------------------MAH

*L.stagnalis* β 1 --------MIHCALATAPARPWTKTRHQHTAHGVRSGAMWGIIVPFFS----ASLMCSLVAVVRC---QQDTDHFANVTN------------------TIDSLLKGYDIR

*C.elegans* β 1 ------------------------MRRSKTRRIFHVSITSLLVSTIFCQNGTKPHNNSTSDQMSSSWSNRSQTMYSNASS------------------LLSDLLLDYDIR

*D.melanogaster* β 1 -----------------------------MTCFTRVGVSCGLFFFLLG---------AQLQLIRCIRKDVLAGRLENVTQ------------------TISNILQGYDIR

*H.sapiens* β1 1 ------------------------------MWTVQNRESLGLLSFPVM-----------ITMVCCAHSTNEPSNMSYVKE------------------TVDRLLKGYDIR

*H.sapiens* α1 1 --------------------------MRKSPGLSDCLWAWILLLSTLT----------GRSYGQPSLQDELKDNTTVFTR------------------ILDRLLDGYDNR

*H.sapiens* γ1 1 -------------MGPLKAFLFSPFLLRSQSRGVRLVFLLLTLHLGNCVDKADDEDDEDLTVNKT-WVLAPKIHEGDITQ------------------ILNSLLQGYDNK

*N.vectensis* 1 -MLKALLYLVLLSTDLVLASSNENSTSVDFRRSADDELIPTTSSFVSTTERLNSTTSGDVYFSSVSPGYSPNGSHHRPTGPYTGPTAKSNSTEELLPYILNMIKTKSDSR

*T.diomedea* β 46 LRPGFGGPALEIGIELILASFDSISEVDMDYTLTMYLNQYWRDERLQF--LANDTV--IDNDTLQVMTLTGAFAEKIWVPDTFLANDKSSFLHDITEKNKMVKLYGNGSL

*M.leonina* β 79 LRPGFGGPPLKIGIELILASFDSISEVDMDYTLTMYLNQYWRDERLQF--IPNGTE--SLNNSLNVMTLTGAFAEKIWVPDTFLANDKSSFLHDITEKNKMVKLYGNGSL

*A.californica* β 4 HKKELKGEPLEIGIEVILASFDSISEVDMDYTITMYLNQYWRDERLQF--LDNDTLDNLDNNTIKAMTLTGAFAEKIWVPDTFLANDKNSFLHDITEKNKMVRLYGNGSL

*L.stagnalis* β 78 LRPSFGGAPLEIGIEVILASFDSISEVDMDYTITMYLNQYWRDERLQF--IFNESLDLGENRSVTTMTLTGAFAEKIWVPDTFLANDKNSFLHDITEKNKMVRLYGNGSL

*C.elegans* β 69 LRPGFGGDALLLTMDIIIASFDSISEVDMDYTLTMYLHQYWTDERLRW----------SNEIPIDEMTLSGEFSQNIWVPDTFLANDKHSYLHEVTERNKMLRINVDGKV

*D.melanogaster* β 55 LRPNFGGEPLHVGMDLTIASFDAISEVNMDYTITMYLNQYWRDERLAFNIFGQYFDDENDDGISDVLTLSGDFAEKIWVPDTFFANDKNSFLHDVTERNKLVRLGGDGAV

*H.sapiens* β1 52 LRPDFGGPPVDVGMRIDVASIDMVSEVNMDYTLTMYFQQSWKDKRLSY------------SGIPLNLTLDNRVADQLWVPDTYFLNDKKSFVHGVTVKNRMIRLHPDGTV

*H.sapiens* α1 57 LRPGLGERVTEVKTDIFVTSFGPVSDHDMEYTIDVFFRQSWKDERLKF------------KGPMTVLRLNNLMASKIWTPDTFFHNGKKSVAHNMTMPNKLLRITEDGTL

*H.sapiens* γ1 79 LRPDIGVRPTVIETDVYVNSIGPVDPINMEYTIDIIFAQTWFDSRLKF------------NSTMKVLMLNSNMVGKIWIPDTFFRNSRKSDAHWITTPNRLLRIWNDGRV

*N.vectensis* 110 LRPKIGRDPVIVYTDMFVLDIGDISEADMEFRVMFFYRQYWQDTRLAY----------DYTGYNKRLALNARMVDYMWIPDIYFVNEKSGIKHDLTKQNEVVRVWPDGRV

*T.diomedea* β 152 VYGMRFTTTLACMMDLHNYPLDNQECTVEVESYGYPMDDIMLYWLNDRDAVTGV---EDVSLPQFSITNYNTINKIETLLTGEYQRLSLVFQLQRNIGYFIFQTYLPSIL

*M.leonina* β 185 VYGMRFTTTLACMMDLHNYPLDKQECTVEIESYGYPLEDIMLYWLNDRKAVIGV---EDVSLPQFSITDYETINKKEELLTGDYQRLSLVFQLQRNIGYFIFQTYLPSIL

*A.californica* β 112 VYGMRFTTTLACMMDLHNYPLDHQQCTVEIESYGYTMDDIVLYWLNDRGAVTGV---EDVSLPQFSIDTYETINKIEELLTGDYQRLSLIFQLQRNIGYFIFQTYLPSIL

*L.stagnalis* β 186 VYGMRFTTTLACMMDLHNYPLDHQECTVEIESYGYTMDDIVLYWLNDRGAVTGV---EDVSLPQFSITNYATINKIEELLTGDYQRLSLIFQLQRNIGYFIFQTYLPSIL

*C.elegans* β 169 AYGMRLTSTLSCSMNLRNFSLDSQNCTVEIESYGYTTSEVLMKW-NYPLAVHGV---EQADVPQFTITGFHTEDSIVSTATGSYQRLSLVFQLRRSVGYFIFETYLPCDL

*D.melanogaster* β 165 TYGMRFTTTLACMMDLHYYPLDSQNCTVEIESYGYTVSDVVMYW--KPTPVRGV---EDAELPQFTIIGYETNDRKERLATGVYQRLSLSFKLQRNIGYFVFQTYLPSIL

*H.sapiens* β1 150 LYGLRITTTAACMMDLRRYPLDEQNCTLEIESYGYTTDDIEFYWNGGEGAVTGV---NKIELPQFSIVDYKMVSKKVEFTTGAYPRLSLSFRLKRNIGYFILQTYMPSTL

*H.sapiens* α1 155 LYTMRLTVRAECPMHLEDFPMDAHACPLKFGSYAYTRAEVVYEWTREPARSVVV-AEDGSRLNQYDLLGQTVDSGIVQSSTGEYVVMTTHFHLKRKIGYFVIQTYLPCIM

*H.sapiens* γ1 177 LYTLRLTINAECYLQLHNFPMDEHSCPLEFSSYGYPKNEIEYKWKKPSVEVADP---KYWRLYQFAFVGLRNSTEITHTISGDYVIMTIFFDLSRRMGYFTIQTYIPCIL

*N.vectensis* 210 FHSIRLSMTASCPMKLHSYPMDKQTCKLSFESFSYPSTELIFKWNKNRGN-KEVQVSDELQIPQFILTEYKTTSEFANFTTGSYSRLTVLFRFERSIGFFLIQTYIPAYL

*T.diomedea* β 259 IVMLSWVSFWINHEATSARVALGITTVLTMTTISNGVRSSLPRISYVKAIDIYLVMCFVFVFAALLEYATVNYTYWGARAKRKAKKLKERERSASLRPRKEDGET---VT

*M.leonina* β 292 IVMLSWVSFWINHEATSARVALGITTVLTMTTISNGVRSSLPRISYVKAIDIYLVMCFVFVFVALLEYATVNYTYWGARAKRKAKRLKEKERSASARRRKEDGET---VT

*A.californica* β 219 IVMLSWVSFWINHEATSARVALGITTVLTMTTISNGVRSSLPRISYVKAIDIYLVMCFVFVFAALLEYAAVNYTYWGARAKRKAKRLRERATSVRRTRVEEPPEPMVNLN

*L.stagnalis* β 293 IVMLSWVSFWINHEATSARVALGITTVLTMTTISNGVRSSLPRISYVKAIDIYLVMCFVFVFAALLEYAAVNYTYWGARAKRKAKRLRERATSV--RKRVDDGDQ---MN

*C.elegans* β 275 IVMLSWVSFWINHEATSARVALGITTVLTMTTISTGVRQSLPRISYVKSIDIYLVMCFVFVFAALLEYAAVNYSYWGRERGKGGGGNEWPVNGANKEDRESAVNVQKWVP

*D.melanogaster* β 270 IVMLSWVSFWINHEATSARVALGITTVLTMTTISTGVRSSLPRISYVKAIDIYLVMCFVFVFAALLEYAAVNYTYWGKRAKKKIKKVKECCPGKIGKSERSETCS-----

*H.sapiens* β1 257 ITILSWVSFWINYDASAARVALGITTVLTMTTISTHLRETLPKIPYVKAIDIYLMGCFVFVFLALLEYAFVNYIFFGKGPQKKGASKQDQSANEKNKLEMNKVQV-----

*H.sapiens* α1 264 TVILSQVSFWLNRESVPARTVFGVTTVLTMTTLSISARNSLPKVAYATAMDWFIAVCYAFVFSALIEFATVNY-FTKRGYAWDGKSVVPEKPKKVKDPLIKKNNT-----

*H.sapiens* γ1 284 TVVLSWVSFWINKDAVPARTSLGITTVLTMTTLSTIARKSLPKVSYVTAMDLFVSVCFIFVFAALMEYGTLHY-FTSNQKGKTATKDRKLKNKASMTPGLHPGST-----

*N.vectensis* 319 IVMLSWIAFWINHTSTPARIGLGITTVLTMTTLTNSARASLPKVSYVKSIEWFLIMCFLYVFMSLVEYGCVSYEV--NRKLKKGVRLPVEAEQEQQTKEPEKQQN-----

*T.diomedea* β 366 FAPMETAVELKE------VGRAPSNNIQSFNLETEDGMNSETQTYRMVPPA--MPRA------------YAHTHVTTTHGYIPTTVVRRRTN---ANSSAPRKRKF----

*M.leonina* β 399 FAPVETAVELKD------VGRAPSNNVQSFNMENEDGLNSEPQTYRMQPSG--MPRS------------YAHTHVTTTHGYIPTTVIRRRTS---NNSTVPRKRRL----

*A.californica* β 329 NSNLEA-VELKEVHRMA-PAPMGITNSQSFNLELDDG-SIETSGFRMAPT---IPRP------------YTSHAPTTAHGYIPSTVVRRRSS----SHVPPRRRRL----

*L.stagnalis* β 398 NTNMDT-MELKEVHMV--PTSVGVTNSQSFNLDLDDG-SGDATGFRVVPP---IPRS------------FTHSHATT-HGYIPTNVVRRRSS----SHVPPRRRRL----

*C.elegans* β 385 SGLMDGVPQPQDRRVEALEEAMSTSNTAAQNNNFESTSKPKKRSSSPIPP---LCRAGNTISEESESPDYPRYSTTSLKGARPHASLNHKTHHLKGRSSARAKRRMTLAR

*D.melanogaster* β 375 --TTEDIIELQDVR----MSPIPSLRRGTYNATLDSI-GTETMNLGKFPPSFRITRN------------YGTGH----------SQLRRRAQ---R--GISTRPRM----

*H.sapiens* β1 362 -----------DAHGNILLSTLEIRNETSGSEVLTSVSDPKATMYSYDSASIQYRKP------------------------LSSREAYGRAL---DRHEVPSKGRI----

*H.sapiens* α1 368 ------------------YAPTATSYTPNLARG-DPGLATIAKSATIEPK---EVKP---------------------------------------ETKPPEPKKT----

*H.sapiens* γ1 388 LIPMNNISVPQEDDYG--YQCLEGKDCASFFCCFED------------------CRT-----------------------------------------GSWREGRI----

*N.vectensis* 422 ---------------------------VMYVMDKEQSANGNSKLRPVFSF---RSRQ------------------------KATQLIKQRSF---SQKEIEGMEPL----

*T.diomedea* β 449 --------LSNFRLKARSIKVKIPRVQDVNTIDKYARLLFPLLFIIFNASYWAV-YLLT-----------

*M.leonina* β 482 --------LSNFRQKAKTMKVKIPRVQDVNTIDKYARLLFPLLFIIFNATYWAA-YTLA-----------

*A.californica* β 413 --------LSNFRQKAKSIKVKIPRVQDVNTIDKYARLMFPLLFIIFNASYWAV-YLLT-----------

*L.stagnalis* β 480 --------LSHFRQKAKSIKVKIPRVQDVNTIDKYARLMFPLLFIIFNTSYWSV-YLLT-----------

*C.elegans* β 492 MNVSMKQSISGIGRRARKVIPTI-RVRDVNLIDKYSRVVFPVCFIVFNLFYWSY-YMMVPS---------

*D.melanogaster* β 447 --------LHALKRGASAIKATIPKIKDVNIIDKYSRMIFPISFLAFNLGYWLF-YILE-----------

*H.sapiens* β1 430 ------------RRRASQLKVKIPDLTDVNSIDKWSRMFFPITFSLFNVVYWLY-YVH------------

*H.sapiens* α1 413 -------------------------FNSVSKIDRLSRIAFPLLFGIFNLVYWAT-YLNREPQLKAPTPHQ

*H.sapiens* γ1 433 ------------------------HIR-IAKIDSYSRIFFPTAFALFNLVYWVG-YLYL-----------

*N.vectensis* 471 -------------------------------TDRYCRVLFPVSFLVFNIIYWILSYNGVSP---------

**Figure S15. MUSCLE protein alignment of ionotropic GABA_A_ receptor homologues from *Tritonia diomedea*, *Melibe leonina*, *Aplysia californica*, *Lymnaea stagnalis*, *Drosophila melanogaster*, *Caenorhabditis elegans*, *Homo sapiens* (β1, α1 and γ1 subunits) and *Nematostella vectensis*.**
